# Hepatitis C Diagnosis: Simplified Solutions, Predictive Barriers, and Future Promises

**DOI:** 10.3390/diagnostics11071253

**Published:** 2021-07-13

**Authors:** Imran Shahid, Abdullah R. Alzahrani, Saeed S. Al-Ghamdi, Ibrahim M. Alanazi, Sidra Rehman, Sajida Hassan

**Affiliations:** 1 Department of Pharmacology and Toxicology, Faculty of Medicine, Umm Al-Qura University, Al-Abidiyah, P.O. Box 13578, Makkah 21955, Saudi Arabia; aralzahrani@uqu.edu.sa (A.R.A.); sskghamdi@uqu.edu.sa (S.S.A.-G.); imanzi@uqu.edu.sa (I.M.A.); 2 Functional Genomics Laboratory, Department of Biosciences, COMSATS University Islamabad (CUI), Islamabad 45550, Pakistan; sidrarehman@comsats.edu.pk; 3 Viral Hepatitis Program, Laboratory of Medicine, University of Washington, Seattle, WA 98195, USA; sajihassan2004@yahoo.com

**Keywords:** hepatitis C diagnosis, point-of-care test, rapid diagnostic tests, HCV core antigen, nucleic acid amplification technologies, one-step screening, reflex RNA testing, dried blood spot, hepatitis C-self testing, HCV elimination

## Abstract

The simplification of current hepatitis C diagnostic algorithms and the emergence of digital diagnostic devices will be very crucial to achieving the WHO’s set goals of hepatitis C diagnosis (i.e., 90%) by 2030. From the last decade, hepatitis C diagnosis has been revolutionized by the advent and approval of state-of-the-art HCV diagnostic platforms which have been efficiently implemented in high-risk HCV populations in developed nations as well as in some low-to-middle income countries (LMICs) to identify millions of undiagnosed hepatitis C-infected individuals. Point-of-care (POC) rapid diagnostic tests (RDTs; POC-RDTs), RNA reflex testing, hepatitis C self-test assays, and dried blood spot (DBS) sample analysis have been proven their diagnostic worth in real-world clinical experiences both at centralized and decentralized diagnostic settings, in mass hepatitis C screening campaigns, and hard-to-reach aboriginal hepatitis C populations in remote areas. The present review article overviews the significance of current and emerging hepatitis C diagnostic packages to subvert the public health care burden of this ‘silent epidemic’ worldwide. We also highlight the challenges that remain to be met about the affordability, accessibility, and health system-related barriers to overcome while modulating the hepatitis C care cascade to adopt a ‘test and treat’ strategy for every hepatitis C-affected individual. We also elaborate some key measures and strategies in terms of policy and progress to be part of hepatitis C care plans to effectively link diagnosis to care cascade for rapid treatment uptake and, consequently, hepatitis C cure.

## 1. Introduction

The burden of hepatitis C infection is still significant, affecting around 71 million people worldwide and accounting for 9.7 million disability-adjusted life years annually [1,2]. Despite the advent and approval of all oral interferon-free direct-acting antivirals (IFN-free DAAs) to treat hepatitis C-infected patients, globally, 400,000 people die annually due to chronic hepatitis C (CHC) associated complications, predominantly as a result of decompensated hepatic cirrhosis or hepatocellular carcinoma (HCC) [3]. These antiviral therapies are highly effective, well tolerable, and produce fewer severe adverse events (SAEs) in treated subjects, but are still expensive and not easily accessible to many hepatitis C-infected patients in LMICs. Interestingly, after the recommendations of pan-genotypic DAA regimens to cure hepatitis C-affected populations, only 5 million hepatitis C patients have been treated with DAAs worldwide and many millions are waiting for active treatment [4]. Furthermore, the typical time course of acute hepatitis C infection can result in divergent outcomes of either self-limited infection (i.e., in 30% of hepatitis C infected individuals, the infection is cleared and resolves without any outcome of infection) or progression to CHC (i.e., approximately 70% acute hepatitis C infected patients progress to CHC), which is often asymptomatic until advanced hepatic disease develops in affected individuals [5]. Consequently, the majority of CHC-infected patients are unaware of their current infection status. In addition to that, vulnerable populations with an identifiable risk factor of hepatitis C infection transmission (e.g., patients who inject drugs (PWIDs), patients in drug harm reduction centers, prisoners, migrant injection drug users (IDUs), sex workers, men who have sex with men (MSM), persons having unprotected sex with multiple partners, persons with a previous history of incarceration, and homeless individuals) and at-risk population groups (e.g., hemodialysis patients, health care professionals after occupational exposure to potentially infected blood, and persons living with a hepatitis C-positive individual) are also largely unaware of their ongoing infection status [6,7]. Surprisingly, similar to human immunodeficiency virus (HIV) screening, no general systemic approach for hepatitis C virus (HCV) testing and screening has been developed, adopted, or implemented worldwide, albeit national initiatives have been introduced by some countries (e.g., Australia, Canada, Egypt, France, Georgia, Iceland, Isreal, Japan, Portugal, Spain, and Qatar); those are on track to achieve the WHO’s elimination goals of hepatitis C by 2030 [8]. Even the hepatitis C elimination strategies are ambiguous both in respect of infection diagnosis and treatment uptake in some resource-rich nations as their national hepatitis care plan exposes serious flaws in their health care systems [9]. Overall, the HCV diagnostic rates are low in general populations even in developed countries that have adopted a routine, population-based HCV screening [10]. Although taking into account the WHO goals of HCV elimination and prevention guidelines in their national hepatitis plan by WHO member nations, millions are missing in hot-spots and highly prevalent areas of HCV transmission in resource-constrained nations and in many LMICs where testing and treatment costs are sky-high, less accessible due to poor health care infrastructure, and have poor-quality management systems for relaying results in laboratory testing services [9].

Molecular diagnostics could not be so sophisticated and advanced if HCV had not been discovered in 1989 by gene cloning method instead of conventional molecular biology and classical virological techniques. Since then, the world has always been seen paradigms in HCV diagnostics where rapid advancement in stat-of-the-art diagnostic tools helped clinicians and physicians to screen and diagnosis HCV in public health care sectors as well as in the premises with poor health care facilities and HCV populations with identifiable risk factors and at-risk patient groups. The traditional and current methods of anti-HCV antibody screening, qualitative/quantitative detection of viral RNA and HCV genotypes identification assays are still the validated, sophisticated, and robust procedures of HCV screening/diagnosis before treatment decision-making and prognosis. However, this set of complex diagnostic tests is not reachable and is time-consuming to the aboriginal populations of resource-rich nations who have limited access to health care systems in remote areas, nor is it accessbile for drug addicts, prisoners, sex workers, and migrants [8]. Similarly, the testing procedures are expensive with centralized lab requirements to process in high prevalent and hot-spot areas of HCV transmission in LMICs with poor health infrastructure of testing and diagnosis. Consequently, undiagnosed HCV cases worldwide represent an important obstacle to achieve the WHO goal of HCV elimination by 2030 [8]. Conventionally, HCV RDTs algorithms included the use of immunoassays that detected HCV antibodies (Abs) or antigens (Ags) and provided results within 30 min by using alternative sampling methods [11]. Recent validation studies of rapid HCV RNA assays based on both standard (i.e., venous blood plasma and serum) and alternative sampling methods (e.g., finger-stick (FS) capillary whole blood, crevicular fluid, and dried blood spot (DBS) sampling) confirmed the acceptable sensitivity and specificity of these assays [11]. However, from the last decade, a paradigm shift has been observed in HCV diagnostic algorithms with an update of the existing methods and the advent and approvals of the new ones in the form of RNA point-of-care (RNA POC), HCV RNA reflex testing, nanoparticle-containing diagnostic agents, HCV self-test assays, and dried blood spot (DBS) sample analyses, which are likely to improve and simplify access to HCV testing and to promote linkage to care and, ultimately, cure [12]. The unique characteristics of these HCV diagnostic algorithms are better screening/diagnosis of HCV-infected individuals, high specificity and sensitivity, availability for multiplexing and polyvalency, short turnaround time, and requirement of small lab infrastructure for usability [11]. The ideal contribution of a rapid HCV molecular diagnostic assay should be the possibility to accurately diagnose active hepatitis C infection in a single visit enabling real-time treatment decisions which can potentially facilitate cure and, subsequently, elimination [13]. Consequently, the implementation of universal HCV screening by simplified HCV diagnostic algorithms in highly endemic areas and high- risks populations worldwide would be pivotal to reduce HCV transmission at the population levels as well as to prevent virus-associated hepatic comorbidities and mortality at an individual level which would ultimately lead to infection cure with enhanced treatment uptake and care management [8].

In this review, we decipher the potential roles of current and emerging simplified HCV diagnostic algorithms to achieve the global goal of HCV diagnosis, treatment, and reduction in HCV-associated mortalities according to WHO set goals by 2030. We will also narrate the unique characteristics of innovative diagnostic concepts and novel ‘test-and-treat’ strategies successfully implemented by the countries as a nationwide policy to test their HCV-infected countrymen. Such policies and recommendations for HCV diagnosis and treatment will be a role model for all those nations who are off track or still behind their targets to achieve HCV elimination by the WHO deadline of 2030. We will also elaborate on the gaps to fill and challenges to meet while linking scaled-up HCV diagnosis to preventing new HCV incidences by reducing existing patient pools and enhancing HCV treatment uptake and, ultimately, cure. Lastly, we will allude to what capacity the current and emerging simplified, user-friendly, and robust HCV diagnostic platforms/devices, pragmatic testing approaches, and innovative diagnostic concepts could be applicable worldwide to test/diagnose every HCV-affected individual and to make rapid progress towards the elimination goals for hepatitis C.

## 2. Why Are We in Need of Simplified HCV Diagnostic Algorithms?

### 2.1. To Achieve WHO Elimination Goals for Hepatitis C by 2030

Even before the emergence of the COVID-19 pandemic in 2019, worldwide, 400,000 annual deaths were reported from hepatic diseases and cancer (i.e., hepatocellular carcinoma; HCC) related to HCV [14]. Worldwide, an estimated 71 million people are living with active HCV infection, and until the fifth unreserved celebration of sofosbuvir (SOF) approval to treat CHC infected populations in 2018, less than 5 million people (3% of 71 million) have been treated or received SOF-based treatment [14]. Despite the COVID-19 outbreak, HCV is still in the limelight now where new HCV infections continue to outpace annual cures. The world health organization (WHO) in 2016 set the global goal of HCV elimination by 2030, considering it a major public health threat [15]. However, achieving this ambitious goal requires a 90% decrease in the new incidence of HCV infection, 90% of HCV-infected patients to be diagnosed, and 80% of those to be treated. Furthermore, a 65% reduction in HCV-associated mortalities needs to be achieved [8]. It predicts that, worldwide, 5 million HCV people need to be treated annually to achieve this HCV elimination target [8]. However, the staggering rates of new HCV infections, low treatment uptake, and high mortality due to HCV-associated cirrhosis and HCC derail the efforts to meet the WHO’s 2030 targets [8]. Furthermore, HCV diagnosis and treatment responses have been profoundly impacted by the ongoing global COVID-19 pandemic due to which the WHO’s elimination targets may seem to be distant abstractions [8]. A study that modeled countries’ trends toward HCV elimination illustrates very interesting data about the size of the HCV epidemic, the sustained virologic response rates (SVR) data of treated populations, HCV-related deaths, and new infections across 91 countries out of 210 [9]. The study estimated the projections to achieve HCV elimination by 2030 or before based on the ratios of patients cured to new HCV incidences [9]. According to this study, 54 out of 91 countries had more new HCV infections than cures in 2016 [9]. The study also demonstrates that countries that can sustain a 5:1 ratio of treatment per new infection will be able to meet elimination by 2030 [9]. Surprisingly, until now, 11 out of 91 countries fall in this category to achieve this goal [9]. In contrast, 47 out of 91 countries can miss this target because one or less than one patient was treated per five new infections in 2017 [9]. Furthermore, the overall decline in epidemic size was less than 1% in 2016, going from 57.3 million to 56.9 million in 2017, and the net cure was 0.43% worldwide in 2016 [9]. It should be noted that the global estimated burden of HCV is 71 million, which is 13 million more than this study analysis [9]. After the approvals and recommendations to prescribe pan-genotypic DAAs to treat HCV from 2016, treatment uptake and the net cure have been significantly increased; however, HCV-associated mortalities and new infection incidences are unfortunately consistent, which makes it an uphill task to achieve HCV elimination by 2030 at this ratio [8]. Diagnostic burnout (i.e., the higher rate of treatment uptake and cure to diagnosed HCV patients than failure to diagnose new incidences) is another problem that could be faced by the countries which are on track to eliminate HCV before or by 2030 [9]. Modeling forecasts for some countries such as Brazil, Spain, and Portugal projects that these countries would soon be in a position to treat all diagnosed HCV patients; however, the undiagnosed HCV people with no symptoms living in semi-urban or rural areas with less access to diagnostic services will remain [9]. If such populations miss HCV screening then, ultimately, the HCV prevalence rate and HCV-associated co-morbidities (e.g., cirrhosis and HCC) will rise, and it may not be possible to treat new infections and prevent HCV-associated comorbidities and mortalities [9]. All this makes HCV diagnosis a vital part of HCV elimination strategies and eagerly demands the implementation of simplified HCV diagnostic algorithms with a focus on areas where treatment uptake levels are higher but a significant number of HCV-affected populations are living with undiagnosed HCV infection.

### 2.2. To Find the Missing Millions Living with Undiagnosed HCV Infection

From 71 million people living with HCV worldwide, it has been estimated that two-thirds of them are living in LMICs with poor access to HCV testing facilities and treatment uptake [10]. Furthermore, worldwide, 19% of people living with HCV have been diagnosed and less than 5% diagnosed with HCV live in LMICs [10]. Surprisingly, only 20% of CHC-infected patients know about their active infection status and only 15% had been treated with DAA regimens by the end of 2017 [9]. Sub-Saharan African (SSA) countries reported 34.4 times more new infections and Central and Eastern Europe had 12.4 times more new infections than cure in 2016 [9]. China has the largest epidemic size with 9.8 million active HCV infections, followed by Pakistan (7.1 million) and India (6.2 million) [9,10]. In the US, HCV is still considered the most common blood-borne infection affecting 2 million people, and more than half of them being reported unaware of their infection [10]. Geographical distribution of new HCV incidences demonstrates that more than half of them live in the Asian Pacific region (29.6 million) and a substantial number within the Western European region (2.4 million) [16]. Overall, 5.6 million people are living with HCV in the European Union (EU) and European Economic Area (EEA) countries [16].

#### 2.2.1. Vulnerable HCV Populations—A Major Threat for New HCV Outbreaks

The vulnerable populations responsible for new HCV incidences belong to people who inject drugs (PWIDs), sex workers, incarcerated people, migrants who inject drugs, and homeless individuals. These key risk populations are hardly accessible to various HCV diagnostic platforms and are often lost during follow-up visits to either HCV diagnosis or treatment uptake. It has been estimated that, worldwide, 67% of PWIDs are infected with HCV and represent a primary source of HCV transmission in developed countries [10]. Among that 67%, approximately 40% represent recent injection drug use (IDUs) and are infected with HCV, and 9% of those recently injected drugs [10]. In Europe, recent data from 11 out of 16 countries estimates 40% of HCV prevalence in current and former PWIDs [16]. Their likely interactions with adult homeless individuals and people already infected with HIV/AIDS transmit HCV infection in those peoples which emerge to two dominant and hardly reached populations to approach and identify in any traditional or current HCV diagnostic settings [17]. Harm reduction interventions including opioid agonist therapy (OAT) for opioid use disorders (OUDs), combining needle and syringe exchange programs (NSEPs), and recently, the administration of pan-genotypic DAAs has raised the hopes to modestly reduce HCV transmission in many countries [10]. Despite this, the prevalence rate of HCV infection has been found to be drastically higher (>50%) among PWIDs in most EU/EEA countries, including young and new injectors [18]. Similarly, among PWIDs, the proportion of undiagnosed HCV infection is likely to be higher, ranging from 24% to 76% in some European countries [19]. It demands an urgent need to initiate mass HCV screening campaigns in identifiable high-risk HCV populations with simplified diagnostic algorithms and extend the existing HCV testing programs.

#### 2.2.2. Correctional Populations—A Potential Cause of New HCV Incidence in Inmates and Infection Transmission to the General Population

In correctional or marginalized populations’, HCV’s seroprevalence has been estimated to be 16.1% in US jail prisoners, from which 10.7% have a confirmed HCV infection by using HCV RNA testing [20]. This disproportionally affected segment of the HCV-infected population in every country could be a prime target for rapid HCV screening and linkage to care to prevent the proliferation of CHC associated co-morbidities in infected individuals and to reduce HCV transmission in other inmates [21]. Although, a tailored approach is needed and seems feasible to test and treat incarcerated people, ‘opt-out’ HCV testing for all inmates has been recommended in the US jails [22]. Despite this, only 13 states have currently started routine HCV screening, and only 40% of inmates were routinely screened according to a survey in 2012 [23]. Several factors contribute to low screening rates in this population; however, the scaling-up of HCV diagnosis and linkage to care is very vital to reduce HCV transmission rates in prisoners.

Men who have sex with men (MSM) are also a key risk group for current HCV transmission in most European countries [17]. An increased number of reports demonstrate acute HCV infection incidences among HIV-infected MSM. Similarly, HCV infections among HIV-negative MSM have also raised concern about the rapid spread of the HCV epidemic among MSM. Although HCV transmission through sexual contact is marginally connected, recent evidence of HCV outbreaks in the US, Europe, and Australia among MSM who denied injection drug use demonstrated HCV as an emerging sexually transmissible agent. [24] Furthermore, high rates of HCV reinfection among MSM who spontaneously cleared HCV infection or who were successfully treated with DAAs have also raised concerns and highlight the significance of sexual behavior in HCV transmission [24]. Migrants have also been contributed to expanding the HCV prevalence pool in EU/EEA countries as a recent analysis reports that HCV infections among migrants account for 14% of all chronic infections [25].

Worldwide, the prevalence of anti-HCV antibodies in pregnant females is thought to be 0.1 to 3.6% which could be an increased risk for adverse neonatal outcomes [26]. At this ratio, vertical transmission of HCV from mother to neonates may occur up to 5% of HCV mono-infection cases and is assumed to be a key transmission source of HCV infection in children [27]. In the US, the number of HCV cases in women of reproductive age (WORA) was reported to double in a period from 2006 to 2014 [28]. According to the estimation in 2014, the HCV prevalence in the US pregnant population was noted to be 0.73%, indicating that 29,000 HCV-infected females gave birth [28]. Routine HCV screening in this population could be more advantageous than risk-based screening, which is sometimes poorly implemented by the physicians and is inadequately sensitive [29,30,31]. The American Association for the Studies of Liver Diseases (AASLD) and the Infectious Diseases Society of America (IDSA) updated guidelines in 2018 that recommend universal HCV screening with each pregnancy, except in settings where HCV prevalence is less than 0.1% [32,33]. It could also be cost-effective while linkage to care in the DAAs era of HCV treatment although DAAs dosage algorithms are not well defined for this HCV-affected population to treat [33]. In the US, HCV infection diagnosis in pregnancy is considered a marker of potential injection drug use (IDUs) in WORA in 2020 [34]. Pregnant females in this population group often do not want HCV testing for reasons including social stigma, discrimination, and criminalization of substance use in pregnancy [35]. A report to identify recent, probable, and/or confirmed HCV cases in WORA revealed that 30% of females were pregnant at the time of diagnosis of HCV exposure, and 28% of them received post-test counseling for confirmatory HCV testing [36]. Knowing one’s HCV infection status in pregnancy could also be useful in reducing the risk of prenatal transmission. Worldwide, it is estimated that 3.5 million children are infected with HCV [27]. It also represents an important pool of unidentified HCV cases, where around 95% of HCV-infected children in the US remained undiagnosed [37]. One study conducted in 119 perinatally infected HCV patients demonstrated that 38% of those aged more than 33 years had developed cirrhosis. The study predicts the importance of early HCV infection identification in children to reduce the proliferation of CHC into advanced hepatic disease in elderly ages [38].

**Figure 1:**
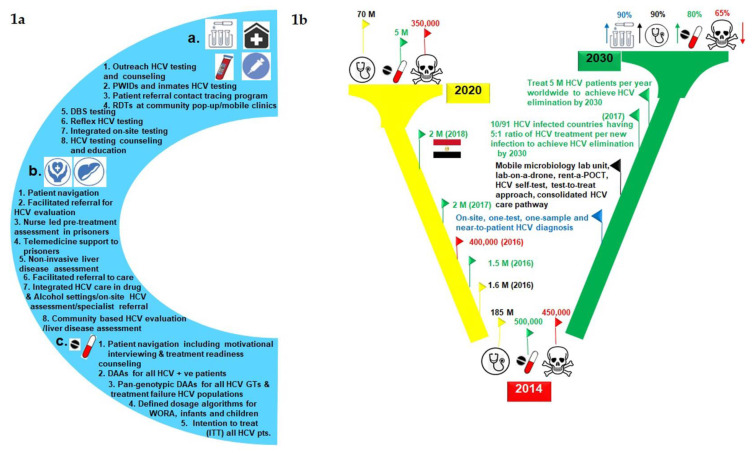
HCV care cascade strategies and WHO goals of HCV elimination by 2030. (**1a**) represents HCV cure pathway strategies, policies, and procedures at diagnostic (depicted in (**1a**) as ‘**a**’), linkage to care (depicted in (**1a**) as ‘**b**’), and treatment uptake levels (depicted in (**1a**) as ‘**c**’). The diagnostic approaches, treatment choices, and measures to be taken, which one seems feasible to make rapid progress toward elimination goals for hepatitis C by 2030, and what should be the key focus are mentioned under each category. (**1b**) represents the comparison of HCV diagnosis, treatment uptake, and mortality before the DAAs era, HCV elimination goals achieved until 2020, and the targets for the next 10 years regarding HCV elimination efforts. The base of the symbolic letter, ‘V’, demonstrates how many people were infected, diagnosed, and died by HCV infection and associated hepatic co-morbidities in 2014 before the inclusion of all oral interferon-free DAAs to be administered for HCV treatment. The yellow pedestal of the letter ‘V’ indicates the achieved goals in respect to hepatitis C elimination by 2020 compared to the intermediary set goals of WHO for this period in 2016 (i.e., 30% decrease in new HCV incidences and 10% reduction in mortality). Green flags indicate the treatment uptake and cure of HCV patients with DAAs onward from 2014. Red flags indicate the decrease in mortality rate after treatment recommendation with DAAs onward from 2014. Yellow flags indicate an escalation of HCV diagnosis after advancing the screening and diagnostic procedures as well as initiating mass HCV screening campaigns. The green pedestal of the letter ‘V’ indicates the priorities to set, policies to design, strategies to implement, and considerable work to do enabling the world to eliminate hepatitis C as a major public health threat before, by, or beyond 2030. The blue flag indicates an urgent need for one-test, one-step, and one visit HCV diagnostic to achieve WHO goals of HCV testing and infection diagnosis (i.e., 90%) by 2030. The black flag indicates novel testing approaches and treatment models to implement while escalating HCV diagnosis and linkage to care in remote areas as well as in vulnerable HCV populations. The green flags depict that how many countries are on track to achieve the set goal of HCV elimination by 2030 and how many patients to be treated with pan-genotypic DAAs to enhance HCV treatment by 80% and 65% reduction in mortality. 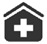 = central lab, 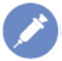 = phlebotomy, 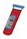 = sample, 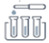 = testing, 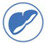 = hepatic disease assessment, 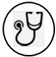 = diagnosis, 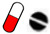 = treatment, 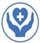 = linkage to care, 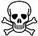 = mortality, 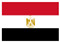 = Egypt. PWIDs = Patients who inject drugs, RDT = rapid diagnostic test, DBS = dried blood spot, DAAs = direct-acting antivirals, GT = genotyping, WORA = women of reproductive age, POCT = point-of-care testing.

As mentioned earlier, despite the expanded availability of generic DAAs with low prices, only 20% of HCV-diagnosed individuals had accessed treatment by the end of 2017 [9]. This reflects the differences in HCV treatment uptake and modifications in HCV care policies in various parts of the world. In some countries, HCV treatment uptake is higher with significant cure rates and, consequently, annual reductions in total epidemic size. In contrast, some others have sparse access to treatment and compromised HCV diagnostic platforms where new infection incidences are escalating the HCV patient pools and epidemic rates [13]. Even in resource-replete nations with access to affordable HCV treatment, the cost and intricacy of current HCV diagnostic algorithms have posed hurdles to people knowing their infection status [13]. All this points out to ideally adopt a ‘one-step’, ‘one visit’, and ‘on-site’ HCV diagnostic approach for high risks HCV populations (Figure 1(1b) [12]).

### 2.3. To Minimize the Steps to HCV Diagnosis

Although in terms of mortality, HCV infections are on a par with HIV, malaria, and tuberculosis, among the top four global infectious diseases, a generalized systemic approach to HCV identification worldwide has not been developed and adopted [8]. Instead of that, the national plans for HCV elimination have been introduced by following the guidelines of the WHO, centers for disease control (CDC), European Centre for Disease Prevention and Control (ECDC), AASLD, and IDSA [8]. For example, In the US, initially, the CDC recommended serology testing in patients with an identifiable risk factor for HCV infection; however, now guidelines extend the anti-HCV Abs screening to all people born between 1945 and 1965 (i.e., baby boomers) [39]. The ECDC in their national screening policies to be implemented across Europe recommends the guidance to reduce the transmission of HCV among vulnerable populations, at-risk, and on-risk population groups [6]. In most LMICs, the development and implementation of national HCV screening policies are compromised due to poor access to HCV diagnostic platforms, the high costs of HCV screening/diagnostic tests, and the number of multiple visits to follow-ups or getting the results [10]. The overall HCV diagnostic rates are still low in the general reported population, even in resource-rich nations that followed and adopted recommended routine ‘population-based screening’ and ‘targeted risk exposure testing’ guidelines of HCV [10]. A study conducted in Europe assessing the HCV prevalence estimated the number of viremic HCV infections to be 3,238,000 in 2015, while only an estimated 1,180,000 have already been diagnosed (36.4%) [40]. HCV diagnostic rates can be as low as 31% in the Czech Republic, 33% in Portugal, and 16% in Turkey [41]. It is expected to be much lower in LMICs and, interestingly, in the US, the HCV diagnostic rate is around 50% [42].

#### 2.3.1. Limitations of the Traditional HCV Diagnostic Algorithms

The plausible justifications for the low rates of HCV diagnosis in the past by using traditional immunoassays and HCV RNA confirmatory tests can be due to the intricacy of classical HCV diagnostic tests, the costs of the required tests, the absence of reliable alternative tests to classical serological and molecular assays, the suboptimal efficacy of interferon (IFN) plus ribavirin (RBV) therapy, the treatment cost, and the poor on-treatment monitoring with IFN/RBV therapy [43,44,45]. However, in recent years, with the advent and approvals of anti-HCV DAAs with promising clinical efficacy, good tolerability, the availability of low-cost generics, and high SVR rates (>95%) in treated patients, the HCV under-diagnosis only remains a predisposing barrier to overcome to achieve HCV elimination. On the flip side of the coin, in the last two decades, the introduction of RDTs, POC tests, RNA reflex testing, and DBS sample analysis as innovative, robust, and pragmatic alternatives for existing HCV screening and diagnosis platforms has escalated the HCV diagnostic rates and potentially impacted the HCV care cascade worldwide [43]. Despite this, the current HCV diagnosis is still performed in two steps in many LMICs and highly prevalent HCV settings; first, HCV seropositivity is confirmed by using either the traditional EIAs or RDTs to determine exposure to the virus. After that, in the second step, ongoing/active HCV infection is confirmed by HCV RNA testing for the patients who are anti-HCV Abs positive [44]. Although in these two-steps HCV diagnostic algorithms seem quite sound and practicable for HCV identification in any patient pool, multiple studies show that 25% to 50% of individuals with an anti-HCV positive result fail to complete follow-up HCV RNA testing to diagnose ongoing infection [46,47]. A possible reason for this drop-off to active infection confirmation could be the historically high costs of HCV RNA testing and the requirement of more laboratory facilities than anti-HCV Abs screening. However, almost all studies conducted on the continuum of HCV care reveals that these two separate testing approaches for HCV diagnosis never brings all positive anti-HCV Abs patients to receive confirmatory HCV RNA testing. Studies from resource-rich nations such as Canada, Australia, and the US suggest that only 46–73% of anti-HCV Abs-positive patients received confirmatory HCV RNA testing [10,13].

#### 2.3.2. Predisposing Factors to Patient Drop-Off with Traditional HCV Diagnostics

**Figure 2:**
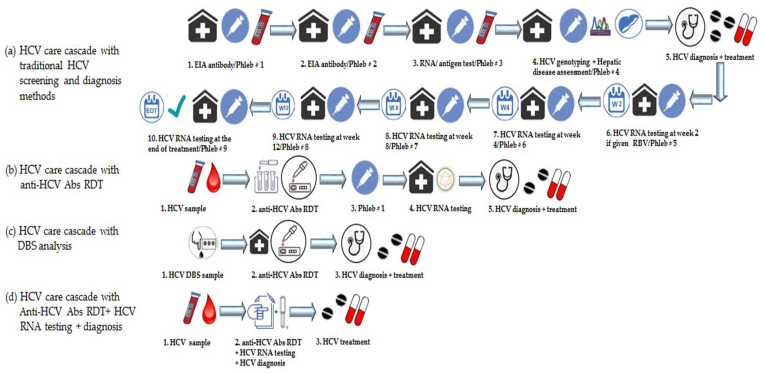
A schematic illustration deciphering the importance of simplified HCV diagnosis solutions to minimizing steps to infection diagnosis and pursuit a single visit diagnosis than the traditional complicated diagnostic pathways in HCV care cascade. (**a**) elaborates the traditional HCV screening and diagnostic procedures in a care cascade which requires 10 single visits, 9 phlebotomies, HCV genotyping assessment, and liver disease staging for disease assessment from HCV screening to infection cure [48]. (**b**) demonstrates the anti-HCV antibodies (Abs) rapid diagnostic tests (RDTs) from HCV screening to treatment which requires four single visits from screening to treatment, one phlebotomy for active infection confirmation, and skipping HCV genotype assessment provided that the patient is prescribed to pan-genotypic DAAs regimens for treatment; however, liver disease staging for disease assessment may require for determining cirrhosis status of the patient (i.e., present or absent; compensated or decompensated cirrhosis), for treatment initiation, treatment type, and length of HCV treatment for HCV GT-3 infected patients. (**c**) shows the dried blood spot (DBS) sample testing by anti-HCV Abs RDTs and the patient receives diagnosis within two visits. (**d**) depicts the anti-HCV Abs RDT, HCV RNA POC test for active viremic infection confirmation, infection diagnosis, and the patient receiving pan-genotypic DAA treatment in a single visit. The prospect of this illustration reflects the importance of a simplified HCV diagnostic pathway (i.e., either choosing (**b**), (**c**), or (**d**) rather than the traditional (**a**) to overcome the obstacles of multiple visits, phlebotomies, and increased time which potentially loses many patients during HCV screening to diagnosis, treatment monitoring, and cures follow-ups in real-world clinical settings [48]. Simplified HCV diagnostics are also helpful to remove the hurdles to scale-up HCV diagnosis in HCV vulnerable populations (e.g., PWIDs, IUDs, MSM) where poor venous access is always a major challenge to draw a venous blood sample [48]. 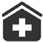 = central lab, 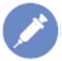 = phlebotomy, 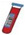 = sample, 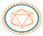 = HCV RNA testing, 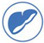 = hepatic disease assessment, 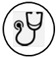 = diagnosis, 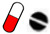 = treatment, 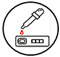 = RDT, 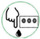 = DBS, 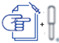 = RDT + HCV RNA + diagnosis, 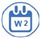 = treatment week, 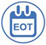 = end of treatment, 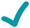 = HCV cure, 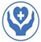 = linkage to care, 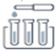 = testing 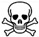 = mortality 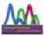 = HCV genotyping. RDT = rapid diagnostic tests, Ab = Antibody, DBS = dried blood spot, POC = point-of-care, RBV = ribavirin, Phleb = phlebotomy.

Several factors relate to this drop-off during active hepatitis C infection confirmation by HCV RNA testing following a positive anti-HCV Abs test result. The picture is also grim in many diagnostic settings, in particular to those areas which have no access or with limited access to DAAs, where multiple patient visits are required from HCV screening to post-treatment monitoring or infection cure confirmation (i.e., according to some studies, 10 visits are involved from initial HCV Ab screening to post-treatment HCV cure confirmation; Figure 2) [15]. Even up to five separate visits are required from initial anti-HCV Abs screening to receipt of an HCV diagnosis from the physicians [13]. Subsequently, a significant proportion of proposed HCV-affected patients may be lost during infection diagnosis, linkage to care, treatment uptake, and post-treatment follow-ups. Furthermore, it complicates the HCV care pathway both in terms of time and costs for many working patients to take the whole day off from their employers, nursing caring management for their kids, and transportation costs for multiple visits. This is evident by the studies that any single drop-off or delay of any of these steps cannot make a confirmed diagnosis of active HCV infection. This situation could also be problematic in remote or rural areas with poor communication facilities and among marginalized populations (e.g., PWIDs, sex workers, etc.) who are fewer in contact with healthcare systems.

Studies predict that many individuals from this HCV-affected patient pool with positive anti-HCV Abs test do not return for confirmatory HCV RNA testing. Similarly, when HCV screening and confirmatory test facilities are available only in central labs, it also requires many costs for the patients to take a lot of time and often travel great distances for test results, diagnosis, and treatment follow-ups. Although the reflex testing rolled out in the US and the UK minimize the steps for HCV identification both in centralized and decentralized testing settings, limited, inconclusive, and murky data are available to infer the impact of it on improving the retention of HCV active infection people in care cascade and enhancing the overall cure rates in the era of pan-genotypic DAAs regimens [15]. In addition to that, this strategy also relies on centralized HCV testing facilities so hepatitis C diagnosis cannot be achieved in a single visit. Despite the continuation of these existing diagnostic approaches, real-life conditions demand a paradigm shift from these platforms to a single visit, one-step, and on-site diagnostic test for active HCV infection in LMICs as well as in high risks HCV populations in developed nations. Such a testing approach will be more favorable for poor people in LMICs both in terms of reduced diagnostic costs as well as time-saving and integrating other services to retain them in care. It has been estimated that 80% of people are living with CHC infection in LMICs and they have to manage to take off a day’s work, arrange for child care, and sometimes travel long distances for HCV diagnosis by current centralized HCV testing platforms. Furthermore, the available RDTs testing platform only detects anti-HCV Abs (i.e., HCV exposure) from oral fluid or FS whole blood samples and does not detect active HCV infection [13,15].

### 2.4. To Facilitate Accurate and Rapid HCV Diagnosis within Minutes

It is a fact that to sustain the progress towards the elimination goals for hepatitis C by 2030, a ‘test-to-treat’ approach for all HCV-affected populations worldwide would be required in any HCV care cascade. For this purpose, the existing HCV diagnostic algorithms could be upgraded and simplified both in terms of specificity and sensitivity as well as in terms of low costs and quick accessibility. Furthermore, novel diagnostic tools with fundamental advancements in their sample capture and detection with quick turnaround time, robustness, and with easiness of transportation are eagerly needed. For a perfect HCV diagnostic algorithm, an HCV patient without any stigma should go to a community health care center, provide the sample, receive the diagnosis, and start treatment immediately—all in a single visit within or under 30 min [49]. In an ideal diagnostic world, such a platform would be very valuable, diagnosing millions of people living with HCV infection and immediately linking them into the appropriate care pathway. To fill this ideal criterion, a POC HCV RNA test with a small handheld device or a small portable instrument will be required in a setting to load FS capillary whole blood sample or self-collected oral fluid onto the instrument by the patient himself or by any health care worker [50,51,52]. The results will be interrupted simply and accurately within 20 min to pursue treatment uptake. In addition to that, such an exemplary HCV RNA POC-test that would allow global scale diagnosis of active HCV infection should be cost-effective ideally less than USD 5 per test including reagent, at scale, and ex-works costs. As mentioned above, the majority of RDTs detect only anti-HCV Abs, and the solely available POC HCV RNA assay is the Xpert HCV viral load test, which is WHO prequalified (PQ) and CE marked. The current Xpert Viral load assay requires a blood sample via venipuncture and has a test turnaround time of 2 h [53]. The efforts are underway to develop a second-generation cartridge of this assay (i.e., Xpert^®^ HCV VL FS assay) while analyzing FS whole blood as a sample with a short turnaround time of approximately an hour with high sensitivity and specificity [54,55]. Although not FDA approved yet, the advent of this testing platform has opened a new horizon to improve and upgrade the existing HCV RNA POC testing platforms and invent truly rapid HCV RNA tests with a turnaround time from 5 to 10 min.

### 2.5. To Facilitate HCV Diagnosis in LMICs

The large-scale or population-based HCV screening in LMICs with classical or traditional EIAs and HCV RNA testing is not feasible and cost-effective due to many technical and administrative reasons. The classical approaches of HCV screening in the form of EIAs and HCV qualitative or quantitative RNA confirmation assays require laboratory infrastructure, facilities, and expertise in their operation. In many, but not all, LMICs, providing such platforms is difficult due to financial constraints at national levels, and a lack of a well-organized quality management system by the government also hampers the access to accurately and timely diagnosis of HCV infected populations [11,12]. In parallel to that, the complexity of current HCV diagnostic algorithms limits their capabilities to initiate large-scale HCV screening programs in many LMICs. Additionally, the affordability of the simplified HCV diagnostic solutions and the insufficient development of current diagnostic tools also restrict the efforts to ensure broad access to HCV care in poor countries. For such premises, decentralized testing approaches by Anti-HCV RDTs and/or POC RDTs seem advantageous and promising over the use of traditional platforms of HCV diagnosis [13,15]. However, providing the tests at the lowest price or free of costs and improving the patients’ retention before and after testing will be integral to achieve massive HCV screening in HCV-endemic LMICs. Interestingly, a huge focus has been levied by the partners engaged in HCV treatment strategies to reduce the cost of DAAs, while little focus has been noticed to negotiate on the costs of HCV diagnostics [13,15]. It is important to mention here that in many LMICs (e.g., Kenya), HCV diagnostic costs (including HCV monitoring and testing for cure) seem to be more expensive than IFN-free DAAs regimens for HCV cure [56,57]. For instance, in Kenya, a three-month HCV treatment course with generic DAA sofosbuvir would cost around USD 260 or less; however, fewer efforts were paved to reducing the costs of nucleic acid amplification test (NAAT) [57]. In this prospect, two countries have already been engaged in improving access to HCV diagnosis while facilitating the screening of HCV-infected populations. In Georgia, 5.4% of the country’s population was infected with HCV infection and most of them were unaware of their active infection status by 2015 [58]. It was the first country who initiated the world’s first program to eliminate HCV with an estimated 90% reduction in HCV prevalence by 2020 [59]. The key features of the program included initiating HCV screening at no cost for vulnerable HCV populations (e.g., PWIDs, blood donors, pregnant females, hospitalized patients, etc.). Then, the individuals with positive anti-HCV Abs were referred to the linkage of care. Although HCV RNA confirmation service is not free of charge in the country, and enhancing public awareness to offering free HCV RNA testing will support the expansion of the testing campaign and will be vital to achieving hepatitis C elimination targets in the country [60]. Second, in Egypt, which is representing the highest rates of HCV prevalence in the world, the government initiated one of the largest infectious disease screening campaigns in history where 49.6 million individuals were screened for HCV over 7 months [61]. The testing platforms were based on the centralized lab settings by using finger-prick anti-HCV RDT tests with results interpretation within 20 min. Then, HCV seropositive patients were referred to a HCV RNA confirmation test for HCV diagnosis and treatment uptake. The interesting fact of this largest HCV screening campaign was the price negotiation for anti-HCV RDT and HCV RNA confirmation tests, which was USD 0.58 per test for the former and USD 4.80 per test for the latter [61]. Although every country needs to design and implement its own HCV elimination strategies by reviewing the availability of HCV diagnostic tools and launching adequate HCV testing, the bottom line message from the large scale HCV screening campaigns from these two countries is that successfully implementing HCV screening program at national levels with low costs and easily accessible anti-HCV RDTs and HCV RNA assays could be helpful to undertake similar initiatives by other countries to curb the burden of this silent epidemic.

### 2.6. To Improve Linkage to HCV Care

The HCV care cascade with current paradigms of HCV diagnostic algorithms seems complicated and often difficult to navigate for many high risks HCV populations [10]. The major factors contributing to drop-off during HCV RNA testing, pretreatment phase, and treatment follow-ups include multiple lab visits, multiple samples of blood draw, disease assessments, and interactions with different health care providers (HCPs) and insurance payers. This type of care cascade from HCV screening to cure can pose hurdles for some specific HCV populations that want to benefit from a specific health care package from a streamlined care pathway because of a high incidence and prevalence of the infection among them, issues with their social determinants of the health (e.g., stigma, discrimination, criminalization), and facing difficulty accessing health care services [10]. Examples of high-risk HCV populations which may increase the epidemic size or enhance the patient pools with new HCV incidences include PWIDs, incarcerated people, homeless individuals, migrants, MSM, sex workers, OUDs, people suffering from some mental disorders, those who live in remote communities with poor access to care, and aboriginal individuals who are historically less engaged in health care [13]. Furthermore, the current HCV care pathway requires well-organized laboratory infrastructure with skilled people clinically capable to diagnose HCV infection, HCV genotyping, assess the staging of liver disease for treatment decision, and treatment monitoring. Perhaps these requirements also create hurdles to mapping an ideal HCV care pathway. An ideal HCV care pathway for CHC-infected patients or vulnerable HCV populations should involve HCV diagnosis, pretreatment work-up, and treatment initiation in a single day [13]. An increase in HCV screening and diagnosis will not significantly impact the efforts to eradicate hepatitis C by 2030 without simplifying or concomitant improvements in the current HCV diagnostic algorithms and enhancing the treatment uptake. With the approvals of pan-genotypic DAA regimens to treat all HCV genotype (GT) patients, less severe HCV infections can be monitored or treated by the primary healthcare providers (PHCPs) with adequate training. It reflects the critical role of PHCPs in expanding HCV care in areas with high prevalence rates of HCV. A U.S. based study analyzed the impact of a “consolidated” HCV care pathway in highly prevalent settings of HCV that required only two visits for patients from HCV screening to receive treatment [15]. According to this model, a positive anti-HCV test was immediately processed for HCV RNA testing, HCV genotyping, and staging of the liver disease during a single visit. Referral to a specialist was required only with moderate to advanced fibrosis (i.e., METAVIR stage ≥ F2) and less severe cases were handled by the PHCPs. According to this scenario, around 40% of HCV patients can be managed by their PHCPs. In comparison to the current HCV care pathway, which requires at least four visits from HCV screening to receive treatment (Figure 2), the ‘consolidated pathway’ reduced the ratio of the patients lost to follow-up from HCV screening to treatment from 71% and 76% to 4% and 5%, respectively [15]. It reveals that minimizing the steps of the HCV care cascade will increase the number of patients who know the status of their ongoing infection, as well as will enable them to immediately link with the care pathway and receive treatment. It will also help to reduce the loss of follow-up visits between HCV screening and diagnosis. Reinforcing a link between HCV screening and diagnosis will ensure better identification of HCV-infected individuals and will improve the treatment uptake rates by the retention of more patients in the HCV care pathway. Although, by the use of current HCV diagnostic algorithms, HCV screening and diagnosis is still a two-step process in a single visit (Figure 2); however, the development of a cost-effective HCV RNA confirmation test or the advances such as RNA reflex testing can be an alternate approach to combine both steps in a single clinic visit (Figure 2). Some resource-replete nations have already been adopting these approaches in their national hepatitis C care plans, such as Australia and France, with higher HCV diagnosis rates (i.e., 75% and 74%, respectively) [15]. Such diagnostic models can be advantageous in decentralized HCV testing platforms in LMICs to reduce new HCV incidences as well as to increase treatment uptake in HCV-diagnosed patients.

## 3. The Existing and Current Paradigms of HCV Diagnostics

**Figure 3:**
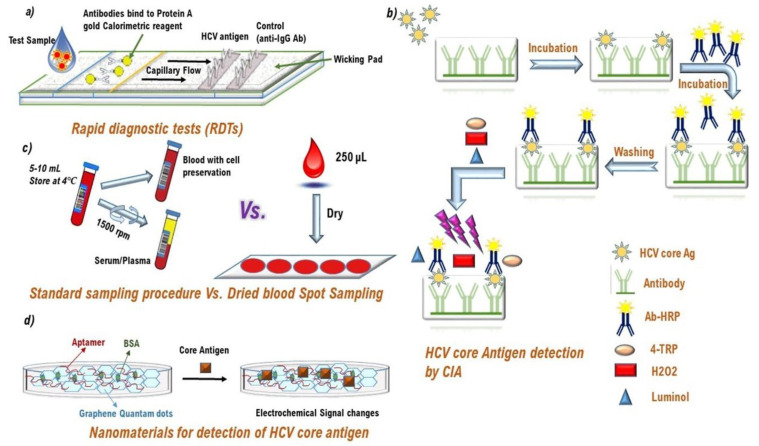
The working principles of currently used HCV screening and diagnosis platforms by detecting anti-HCV Abs, cAg, and RNA. HCV enzyme immunoassays (EIA) are used in three categories to detect HCV antibody, HCV core antigen, or multiple HCV antigen detection. (**a**) depicts the principle of anti-HCV antigen detection by a rapid diagnostic test platform while (**b**) deciphers the principle of HCV core antigen detection by CIA. (**c**) illuminates the advantages of HCV dried blood spot sampling over standard sampling procedure for hepatitis C screening. (**d**) demonstrates the significance of nanomaterials for the detection of HCV core antigen. IgG = immunoglobulin G, BSA = bovine serum albumin, Ab-HRP = Antibody horseradish peroxidase, H_2_O_2_ = hydrogen peroxide, ClA = chemiluminescence assay.

In the pursuit of achieving the WHO’s HCV elimination goal as a major public health threat by 2030, efficient, reliable, and simplified diagnostic algorithms are urgently needed [62]. As such, simplified, robust, and sensitive HCV diagnostic tools that can scale up on-site and a single-visit HCV diagnosis in a single-test approach will also fully assist linkage to care and treatment uptake. Here, in first, we will briefly overview the traditional and current paradigms in HCV diagnostics, which are widely used in centralized and decentralized laboratory settings, in public health care centers, and nontraditional settings, to screen and diagnose at-risk and on-risk HCV-affected populations and to capture individuals from vulnerable HCV populations who cannot access or actively engaged in health care services. We will also discuss HCV diagnostics in the pipeline to simplify, decentralize, and integrate diagnosis into existing healthcare infrastructure (e.g., sexual health clinics, drug harm reduction centers, primary health care, self-testing at home), and the development of innovative diagnostic devices and tests to be suitable for the use in emergency departments, obstetrics centers, surgical and psychiatric wards, dental clinics and pharmacies. Some of them have already been approved for WHO prequalification (PQ) and are CE marked for EU standards and some are in the pipeline to be approved soon. An unexpected delay for the approval of some diagnostic platforms was experienced in 2019 because of the emergence of the COVID-19 pandemic, where the companies rushed to market diagnostics for the SARS-CoV-2 virus (Figure 3 shows the working principle of the currently used HCV screening and diagnosis platforms with their working principles. While not exhaustive, and beyond the scope of this review, the working principle of each test has been comprehensively discussed in the reference study [63]).

### 3.1. The Classical HCV Screening and Diagnosis Approach

The traditional standard protocol for HCV diagnosis is based on a preliminary serological test (e.g., by using an enzyme immunoassay (EIA)) for anti-HCV Abs detection accompanied by a confirmatory nucleic acid testing (i.e., NAT) for HCV RNA detection in serum or plasma samples of testing individuals [11]. Third-generation EIAs use a multi-antigen format of HCV structural protein (e.g., core) and nonstructural regions (e.g., NS3, NS4, and NS5). Generally, EIAs are considered reference standards and are highly sensitive and specific (>99%) assays to detect anti-HCV Abs; however, in some exceptional clinical settings (e.g., HCV hemodialysis patients, HIV and HBV coinfection, other immunocompromised patients, and regional differences in assay performance), sensitivity issues and false negatives have also been reported with anti-HCV Abs testing; albeit, current data remain limited. Furthermore, these EIAs serve only as a screening tool and cannot identify individuals with active HCV infection among those who have been naturally resolved the infection and no longer viremic (i.e., acute HCV patients) [11]. HCV RNA testing (both for HCV genome detection and quantification) is based on real-time polymerase chain reaction (Real-Time PCR) or transcription-mediated amplification (TMA) techniques. The testing platforms can be fully or partially automated. With the well-established limit of detection (LOD; 15 U/mL), and a lower and upper limit of quantification (i.e., LLOQ; usually equal to LOD and 10^8^–10^9^ IU/mL, respectively), the range of linear quantification of HCV RNA detection assays widely supports and is well suited for the clinical needs to diagnose the infection, treatment monitoring, and post-treatment follow-up [11]. EASL commends for using sensitive qualitative or both qualitative and quantitative molecular assays for HCV confirmation within the lower limit of detection (i.e., 15 IU/mL) [64]. Afterward, in 2018, EASL devises for the use of large-scale qualitative RNA detection assays with the detection limit of ≤1000 IU/mL in LMICs as well as in some specific settings in resource-replete nations. As mentioned earlier, 30% of individuals exposed to HCV may naturally resolve the infection within 6–12 months, which means that they will be undetectable for HCV RNA; however, they will be positive for detectable HCV antibodies that persist lifelong. Consequently, HCV RNA testing is required to confirm active infection in such populations. In 70% of CHC-infected individuals, natural clearance of virus occurs rarely after 6–12 months post-exposure; hence, a positive HCV RNA result would be adequate for active infection diagnosis even years after HCV exposure [11]. However, the costs of these molecular RNA confirmation assays for HCV genome detection vary widely across the world, being even more costly than antiviral treatment in many countries. Furthermore, this complex set of two-step HCV diagnostics can take several days or sometimes even weeks to interpret diagnosis, which may result in patient drop-off and thereby decreasing the overall number of HCV cases diagnosed [11]. HCV genotyping is still recommended in decision making to choose the right DAAs for treatment, its efficacy, and duration; however, with the advent and approvals of pan-genotypic DAA regimens to treat HCV, pre-genotyping may no longer be recommended in HCV treatment guidelines (except for HCV genotype 3 cirrhotic patients who usually need longer treatment duration and often require the addition of RBV to DAA regimens to prevent treatment failure) [65]. To prevent the emergence of drug resistance associated with certain DAAs, HCV RAS (resistance-associated substitution) testing is also recommended in selected settings (e.g., previous treatment failure to first or second generation NS3/4A protease or NS5A inhibitors, HCV genotype 3 infection patients, and decompensated cirrhotic patients) by using traditional population sequencing or deep-sequencing (i.e., next-generation sequencing; NGS) based methods with a cutoff value of 20% and 0.1%, respectively [12]. The challenges also remain in the accessibility, affordability, and availability of these testing approaches in LMICs with poor infrastructure for testing in their health care system and even need to address the gaps between HCV screening and diagnosis in resource-rich nations where HCV-vulnerable populations have limited access to the healthcare system due to certain social determinants of health.

### 3.2. The Current and Emerging HCV Diagnostic Platforms Involving RDTs, POCT, DBS, and HCV Self-Test Approaches

It is evident that to achieve the ambitious goal of HCV elimination by 2030, HCV diagnostic algorithms must be simplified and updated with innovative tools to better identify high-risks HCV populations to reduce new HCV incidences at the population level and preventing the progression of HCV-associated co-morbidities and mortalities at an individual level. The ultimate benefit of this dynamic HCV transmission model (i.e., “cure as prevention”) will increase the retention of diagnosed patients to linkage to care and treatment uptake. From the last two decades, new horizons have been opened and adopted for the improvements in HCV diagnostic algorithms with the fundamental advancement in each diagnostic assay component by considering novel assays chemistries and nanotechnologies for sample analysis, and utilization of numerous matrices and microfluidics to allow miniaturization [50,51,52].

**Figure 4:**
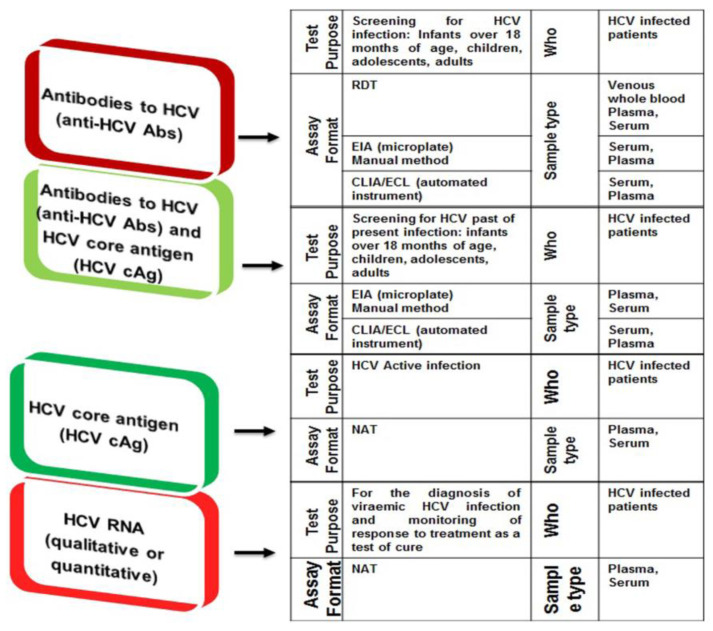
HCV diagnostic platforms with their purpose of use, assay format, sample type, and for which type of test to be used. WHO and international HCV diagnostic and treatment guidelines releasing organizations recommend three types of HCV screening and infection diagnosis testing strategies for general HCV-affected populations and high-risk HCV populations [48]. RDT = rapid diagnostic test, EIA = enzyme immunosorbent assay, EIA = electrochemiluminescence, CLIA = chemiluminescent immunoassay, NAT = nucleic acid testing.

Point-of-care tests (POCTs), including immunological POCT with the rapid diagnostic tests (RDTs), non-immunological POCT based on NAT (both qualitative and quantitative), and serological assays that detect and quantify HCV cAg as an alternate to HCV RNA detection and quantification, have shown their clinical diagnostic promise both in terms of specificity and sensitivity in real-world HCV testing. Recently, the development of POCT and RDTs based on the use of dried blood spot (DBS) samples analysis is also representing an assiduous intervention to scale up HCV testing and linkage to care in vulnerable HCV populations. The rationale to HIV self-testing, which is considered as a safe and effective approach to increase HIV infection diagnosis and treatment uptake in key populations, is that HCV self-test approaches can also be an additional way to accelerate universal screening campaigns in settings with limited human resources, primary care, and at-home settings [66,67]. The transformation of HCV testing from large equipment and portable devices to smartphone-assisted diagnosis either to adopt reading devices or inclusion of biosensing chips to mobile devices are also in the pipeline and promises to be marketed soon to maximize HCV testing in rural and remote communities as well as in LMICs. The additional benefits to expanding smartphone HCV diagnostic innovations can be to improve HCV treatment by collecting and maintaining HCV surveillance data in remote and resource-constrained settings, by increasing telehealth capacity, and by more easily implementing treatment interventions to escalate HCV treatment uptake. Albeit many of these novel diagnostic tools have already been developed and implemented for other viral infections such HIV and Zaka, there is a big hope to quickly translate and transform these tools and technologies to HCV diagnosis and management with a positive commitment to implement those in real-world clinical settings [66,67]. Figure 4 demonstrates the three types of HCV screening and infection diagnosis testing strategies for general HCV-affected populations as well as in high-risk HCV populations as recommended by the WHO and other international organizations releasing HCV diagnostic and treatment guidelines.

#### 3.2.1. Immunological Point of Care (POC) Rapid Diagnostic Tests (RDTs)

As an alternative to classical HCV virological testing using blood serum or plasma from venous puncture, point-of-care (POC) tests have already been developed that allow HCV screening and diagnosis near to the site of patient care and tests based on the dried blood spot (DBS) samples analysis.

##### Pros and Cons of Immunological HCV POC RDTs

HCV POC testing has become a common diagnostic tool in resource-replete nations as well as in LMICs during the last 10 years where those have been potentially impacted on the quality of HCV diagnosis, system redesign, and linkage to care. Most POC tests are based on the immunoassay principle, which qualitatively detects various HCV markers of infection including antigens and antibodies in a limited time frame, also known as “rapid diagnostic tests (RDTs) [11]. In contrast to traditional HCV testing, POC RDTs use alternate sample matrices to plasma or serum such as fingerstick (FS) whole blood or crevicular fluid (i.e., saliva or oral fluid). The on-site processing of serum or sterile plasma samples is sometimes not trustworthy or feasible and shipment requirements to centralized lab settings may complicate logistics. In addition to that, during two-step anti-HCV diagnosis, patients often lost to follow-up, and sometimes have to attend four or five visits to get a full diagnosis [13]. Difficult venous access in PWIDs and IDUs is also a major barrier to collecting serum or plasma samples for HCV screening and many of them are not present for testing at all [13]. POC RDTs reduce the non-attendance to off-site phlebotomy, which provides instant diagnosis results and can be useful to enhance patient counseling, education, and linkage to care [15]. Detection of antibodies in oral fluid is relatively cost-effective, noninvasive, more convenient, and a safer method as compared to the serum-based specimen, and it is useful for HCV screening in remote/and outreach settings [67,68]. In addition to that, POC anti-HCV Abs RDTs do not require much investment in laboratory infrastructure and can be performed with minimal maintenance costs and reagents.

However, the acceptability of RDTs for clinical performance needs to meet the high standards of analytical performance in terms of accuracy, robustness, sensitivity, specificity, short turnaround time, and reproducibility. The clinical performance of individual anti-HCV Abs RDTs is heterogeneous and varies widely [67,69]. For instance, the clinical sensitivity for OraQuick^®^ HCV RDT (OraSure Technologies, Bethlehem, PA, 18015, USA) has been demonstrated to be excellent in both FS whole blood and in oral fluid (i.e., 99.4% and 97.6%, respectively); however, Labmen HCV test (Turklab, Izmir, Turkey) showed poor sensitivity in FS whole blood (i.e., 63.1%) [70]. Similarly, significant variability has also been demonstrated at the manufacturer level, the sample type (i.e., fingerstick whole blood vs. oral fluid), and at pre-analytical conditions (i.e., sample and reagent preservation conditions) [67,71]. On the other hand, anti-HCV RDTs showed better positive and negative predictive values in studies conducted in resource-rich nations as compared to developing nations, which could be partly explained based on the variations of HCV prevalence in targeted populations. Hence, the use of POC anti-HCV RDTs is cautiously recommended to ensure that the individual RDT approach works in specific testing settings and can be limited to only qualitative “yes or no” results, costs, low throughput, subjective interpretation of results, variations in test sensitivity and no traceability [72]. Usually, these approaches were designed to be used at the sites of patient care including intensive care units (ICUs), outpatient clinics, drug harm reduction centers, medical emergency rooms, or even at patient homes for self-testing. In these settings, such testing approaches are able to provide testing results at the time of testing without the need for a follow-up visit to receive diagnosis results. Furthermore, patients may start treatment uptake after an immediate discussion with the health care provider (HCP) and may benefit from other treatment opportunities [72]. Overall, there will be an increase in health care accessibility, which can lead to curing a large number of HCV patients in a single visit. However, concerns have been raised about the performance limitations of some anti-HCV RDTs in clinical settings, so should be carefully recommended in real-life clinical conditions. In large-scale HCV testing, such approaches are suited for decentralized settings to reach high-risk HCV individuals who may not access or often remain outside of the traditional health care system.

The most widely used anti-HCV Ab RDTs are lateral-flow immunoassays, which are easy to use, simple, and have a short turnaround time of 20 min for result availability [63,67]. Most of the existing anti-HCV Abs RDT platforms are WHO prequalified, US FDA approved, and are EU CE-marked. The first RDT approved by the US FDA was the OraQuick^®^ HCV rapid antibody test (OraSure Technologies, Bethlehem, PA, 18015, USA) which detects HCV Abs from serum, plasma, and capillary whole blood samples collected by venous puncture and FS samples in patients aged ≥15 years. By 2020, two RDTs were WHO-prequalified: OraQuick^®^ HCV rapid antibody test and SD Bioline HCV. High sensitivity and specificity of OraQuick^®^ for HCV Abs detection have been demonstrated after testing in the US, Europe, South Korea, and many other countries of the world [73,74]. The findings of a meta-analysis involving 47 studies with total samples of 90,008 explored 98% of the pooled sensitivity of OraQuick^®^ HCV RDT in whole blood either collected by venous puncture or FS and a pooled specificity of 98% [73,74]. However, the pooled sensitivity and specificity were measured at 94% and 100%, respectively, with oral fluid HCV samples. The OraQuick^®^ HCV RDTs are the most sensitive and specific anti-HCV Abs POC tests rather than their available competitors in the market, which express different sensitivities and specificities [11]. Some false negatives have been reported in capillary whole blood or oral fluid in patients infected with HIV/HCV confection, possibly due to the lesser amount of IgG in oral fluid of HIV/HCV-infected patients with residual HIV replication (i.e., HIV RNA load >200 copies/mL) [11]. All four international HCV screening/diagnosis and treatment guideline releasing organizations including WHO, AASLD, IDSA, and EASL recommend the use of RDTs based on serum, plasma, capillary whole blood, or oral fluid testing for HCV screening and further linkage to care in out-reach populations, high-risk patient pools (e.g., PWIDS, migrant IDUs, and prisoners, etc.), in communities with low HCV prevalence, and resource-constrained nations (Figure 4) [63,66].

#### 3.2.2. HCV Core Antigen (cAg) Detection as an Alternate to HCV RNA Testing

From the last decade, there is a growing interest in the use HCV core antigen (cAg) assay as a potentially stable and affordable alternate to HCV RNA testing for the diagnosis or confirmation of active hepatitis C infection. HCV cAg can be detected in the bloodstream within two weeks of infection, which is far earlier than anti-HCV Abs detection (i.e., which are usually detected within or after 10 weeks to HCV exposure) [12,63]. Clinical studies strongly correlate HCV cAg levels equivalent to HCV RNA levels. In one meta-analysis, the comparison of HCV cAg detection with Abbot HCV cAg assay and the ORTHO ELISA-Ag test to HCV RNA revealed that the assay has a sensitivity up to 93.4% and 93.2%, respectively, with very high specificity (>98%) [12]. In addition to that, being a part of HCV virion and detectable in serum of HCV patients, HCV cAg testing does not require dedicated specimens as are needed for HCV RNA testing. Although less sensitive than HCV RNA testing (i.e., missed infections have been reported in clinical validation as well as in real-life clinical data), the real-world data decipher its adequate performance for detecting CHC infections and post-treatment confirmation of hepatitis C cure. A recent systematic review based on the HCV cAg analysis of 8136 samples explored that 0.56% of them were cAg positive and HCV RNA-negative, while 3.52% were HCV RNA-positive and cAg-negative [75]. Furthermore, it is easy to run an Architect^®^ HCV Ag assay EIA on an Architect^®^ device (Abbott, Chicago, IL, 60064-3500, USA), both for qualitative and quantitative analysis. In one study conducted in Cameroon (i.e., The French ANRS 12336 study), the Abbot HCV cAg quantification assay showed high performance in both HIV/HCV and HBV/HCV coinfected patients [76]. This assay is not yet FDA approved but is CE marked with a dynamic range of HCV cAg quantification from 3 to 20,000 fmol/L [12]. However, the LLOD of this assay corresponds to approximately 500–3000 HCV RNA IU/mL and is genotype-dependent. Nevertheless, at this cutoff level, the assay may cover the vast majority (>95%) of CHC infections [11]. Furthermore, being less costly than HCV NATs, cAg levels can be used as a surrogate biomarker of HCV replication for diagnosis, in treatment decision making, and for the on-site and post-treatment monitoring as recently recommended by the WHO and EASL guidelines. In a cost-mapping study in the UK, it was evaluated that a NAAT cost was much higher (i.e., US$ 108) than for HCV cAg assay (i.e., USD 23.4) including the kit, operators, and ex-works costs [77]. In another study in Egypt, the HCV NAAT test’s cost per patient was estimated to be at USD 141 vs. USD 19.8 for HCV cAg [78]. A POC HCV cAg assay Daktari™ system (Daktari diagnosis^®^, Cambridge, MA, USA) was launched in 2018 with a turnaround time of 30 min and test cost likely to be USD 10 to USD 20 [79]. Another advantage of HCV cAg over NAAT is the stability of the surrogate marker at room temperature for 96 h.

The cAg detection is dependent on centralized laboratory infrastructure with sophisticated laboratory equipment, which could not be feasible, easily accessible, and cost-effective in many LMICs; however, it can be effective to improve HCV care access in under-resourced health settings. Another disadvantage is that most validation studies for cAg test specificity and sensitivity were conducted in resource-replete countries within reference laboratories and are missing data for genotype 4, 5, and 6 cAg detection [63,66]. Some false negatives have been reported in HCV GT-3-infected populations with a plausible justification of viral polymorphisms in core nucleotide sequences, which hampers the detection of HCV cAg. It is unlikely that the Architect^®^ system is suitable for centralized lab facilities and access often remains limited in many settings. Furthermore, bulk-volume pricing of HCV RNA PCR tests continues to be reduced in many LMICs (i.e., USD 14–USD 25), which means that for the short-term goal of HCV diagnosis in LMICs, HCV NAT will be a more affordable option. Centralized HCV cAg testing could be a reasonable approach in middle-income countries where testing platforms are already in operation and upfront investment is not required for instrument purchase and other lab facilities. Recent efforts to develop some challenging platforms such as an HCV cAg rapid diagnostic lateral flow test by an immunoassay detection is also in the pipeline, with an early stage prototype being developed by the efforts of Global Health Technologies (Northwestern University, Evanston, IL, 60208, USA), Abbott Diagnostics (Des plaines, IL, 60018, USA), and Qoo Labs (Sorrento Valley Blvd, San Deigo, CA, 92121, USA) [66].

#### 3.2.3. Non-Immunological Point of Care (POC) Rapid Diagnostic Tests (RDTs)

The intricacy of the current HCV diagnostic algorithms limits their capability to advance and scale up HCV screening programs in LMICs and even difficult-to-reach populations in developed nations. Even though, if HCV screening can be done, limited capacity to refer to health care specialists (HCP) for treatment uptake and on- or post-treatment monitoring often represents an additional barrier to HCV cure (e.g., in some centralized lab settings, it requires 10 visits from HCV screening to post-treatment follow-ups; Figure 2). This means that the simplification of the HCV care cascade can transfer the extra burden of HCV diagnosis and treatment uptake from classical HCPs (e.g., specialized infectious disease centers and hepatology clinics) to PCPs for most HCV patients or likely for all patients in community health care [15]. In addition, the lack of cost-effective, simple, and efficiently deployed HCV diagnostic tools in LMICs also prevent the undiagnosed or even patients with ongoing HCV infection from accessing HCV care. It reflects the need for developing and utilization of innovative and decentralized but simplified HCV diagnostic tools for outreach sites in resource-rich nations as well as in LMICs to initiate large-scale HCV diagnosis campaigns and to immediately link infected populations to HCV care cascade [13]. In this scenario, a transformational simplification approach including three main tools in one diagnostic test as a one-step procedure can be pragmatic, laudable, and approachable in the high prevalent HCV population both in developed countries and in LMICs, provided that the cost of the test is affordable [66]. These approaches may include a reliable HCV cAg test amalgamated with a novel POC-RDT based on HCV RNA detection and quantification with the potential to skip HCV genotype identification in the context of current HCV treatment with pan-genotypic DAAs. Although it seems unachievable, with the recent developments in HCV diagnostics, we must be optimistic with a positive commitment about this [66].

##### Advantages and Limitations of HCV RNA POC RDTs

Non-immunological RNA POC assays are now available, which may confirm active HCV infection both qualitatively and quantitatively in a single visit. Several existing RNA POC assays and platforms can be modified and adopted to diagnose active HCV infection and some are under late-stage development (e.g., Xpert^®^ HCV VL FS assay, TrueNAT HCV PCR, and Genedrive^®^ HCV RNA) with more sophistication in sample handling for short turnaround time and improved diagnosis accuracy [12,15]. In an era of highly effective pan-genotypic DAAs available for HCV cure, quantitative HCV RNA assays have become less significant and can be replaced with less expensive qualitative HCV RNA POC assays. The random access to samples (i.e., does not require batch processing), semi-portable, modular, and an automated aspects of RNA testing favors these devices to be operated in a single step and same-day diagnosis interpretations [66]. However, the need for venipuncture, the turnaround time for results, and the initial up-front instrument costs, as well as higher cost per test than anti-HCV Abs RDTs makes those less relevant in low HCV prevalence settings. In contrast, the testing platform and assay approach seem quite attractive for HCV-vulnerable populations (e.g., PWIDs, migrant IDUs, and prisoners) in developed nations. Such testing platforms with the rapid response of the same-day results and being simple enough to be operated by the personals without an essential background in RNA diagnostics or virology can be useful as a single visit diagnosis in settings that do not require specific laboratory infrastructure or expertise in their operation. These test set-ups should be considered as a two-step strategy after screening with Anti-HCV RDTs in premises where centralized lab settings are not available and one-step diagnosis could be theoretically feasible provided that the test costs are down. However, the already approved and marketed HCV RNA POC assays require venipuncture for blood samples, which could be challenging without the services of a phlebotomist and sample draw from PWIDs with poor venous access. Similarly, it is still controversial that whether an HCV RNA POC assay will be eligible to achieve a cheap enough price to be used as a first-line assay in decentralized HCV testing settings. In this perspective, cost-mapping studies are urgently needed to explore whether a one-step or two-step test with an RNA or HCV cAg quantification and diagnostic approaches such as a laboratory or POC test will be cost-effective and affordable in settings with high-risk HCV populations and in LMICs with high HCV prevalence [63,66].

The Xpert HCV Viral Load test (Cepheid, Sunnyvale, CA, USA) was the first RNA POC assay to receive WHO PQ and CE-marking [53,63]. It was the first plasma-based assay to detect active HCV infection and uses a single-use viral load cartridge to extract, amplify, and detect HCV RNA by fluorescent reverse transcriptase PCR when the cartridge is loaded into a GeneXpert instrument, with a total turnaround time of 108 min [66]. Several existing platforms such as the Enigma Minilab (Enigma Diagnostics Ltd., 5555 Oberlin Drive, San Diego, CA, 92121,USA) and DxNA GeneSTAT System (DxNA LLC, Ste 201, St George, UT, 84770-7492, USA) can also be adopted to diagnose HCV. Several other RNA platforms using alternate amplification technologies such as reverse transcriptase loop-mediated isothermal amplification have commercially available assays for other viral infections such as Alere I and Alere q for HIV-1/2 early infant diagnosis (Abbott Diagnostics) [66]. The diagnostic assays based on this amplification technology may reduce the assay turnaround time and platform complexity and provide a visual result facility. The capacity to add HCV RNA assays and integrate with already approved molecular RNA POC platforms (i.e., multianalyte) will increase competition in the field of HCV RNA POC assay development while expediently reducing prices rather than the launch of a new test platform. Furthermore, the development of a true HCV RNA POC NAT to detect active HCV infection from FS whole blood outside of a laboratory setting will have a greater impact. Several studies have documented the higher sensitivity (i.e., 94–98%) and specificity (98–100%) for HCV RNA quantification in venous blood samples by using the Xpert Viral Load assay platform [54].

A finger-stick (FS) capillary whole blood version of Xpert^®^ HCV viral Load assay has also been developed with good sensitivity and specificity output in the real-world clinical setting [55,66]. In a study conducted on FS blood samples collected from people attending drug health and homelessness services in Australia, the VL FS Xpert viral load assay demonstrated 98% and 99% test sensitivity and specificity, respectively [12,55]. Although the total turnaround time including sample collection was not ideal (i.e., 2 h), a version with a total time to result of 1 h or less than that is also under late-stage development. The short turnaround time with this assay version can be used to enhance the number of tested HCV patients and diagnoses in a single visit. GeneXpert Omni (Cepheid, Sunnyvale, CA, USA) is a new and advanced iteration of the Xpert Viral Load device that may detect and quantify HCV RNA from 100 µL of capillary whole blood in a single-use cartridge within an hour, making it the first true POC molecular test [12,13]. The clinical performance of this new device was comparable to other already existing HCV RNA POC assays in the market with an LLOD 35 IU/mL and LLOQ of 100 IU/mL, respectively.

#### 3.2.4. Nanocomposites as HCV Diagnostic Approach

Another important and advanced parameter for the diagnosis of HCV is nanotechnology specifically relating to the POCT system due to the least requirement of sample volume and equipment in addition to their time-affordability, sensitivity, and cost-effectiveness. This paper-based diagnostic system does not exhibit transport and storage issues [63,80,81]. In a study, a sensitive aptamer conjugated graphene quantum dots (GQD) nanocomposite diagnostic system was developed. An electrochemical impedance spectroscopy (EIS) scheme was used to implement GQDs as nanoconjugate for ultrasensitive diagnosis of HCV cAg with the detection limit of 3.3 pg/mL [82]. Such aptasensor-based nanocomposites might serve as a specific, sensitive, and rapid detection system for the HCV cAg diagnosis and can provide a potent strategy in clinical diagnosis. In another study, a potent diagnostic approach for the detection of HCV nonstructural NS3 protein using a nanocomposite encapsulated aptamer probe was designed. Using quantum dots with the carboxylic group as imaging probe and 5′-amino group-modified RNA oligomer as capturing probe, HCV NS3 was detected visually on-chip (Figure 3d). Such nanoparticle-based RNA aptamer conjugating systems possess high specificity and sensitivity with a 5 ng/mL detection limit for NS3 protein [63,83]. However, limited data are available regarding HCV detection through nanotheragnosis as a new paradigm of HCV diagnosis. Further research might enhance the utilization of nanotechnology tools in HCV clinical diagnosis.

#### 3.2.5. Multi-Disease Analyzers

Multi-disease analyzers are used to perform integrated diagnosis of HCV, along with other pathogens such as *Treponema pallidum* (syphilis) and HIV [84]. Low sample volume and efficiency for the detection of multiple pathogens at the same time make this technique favorable. The obvious advantages of this approach also include the polyvalency of various tests by using lower sample volume, fewer FS pricking if capillary whole blood is used, cost-effectiveness for multi-testing, and less time required to perform a series of tests. The testing approach has already been proclaimed for the screening of various acute illnesses to help in outbreak settings but could be considered for the screening of chronic illnesses. Multiple RDTs are in the process of development for anti-HCV/anti-HIV, anti-HCV/HBs Ag testing, and for anti-HCV/syphilis/anti-HIV detection on the same assay platform [84]. However, more research data are required to depict their diagnostic sensitivity and specificity before their implementation in real-world clinical diagnosis and their impact on HCV care management.

#### 3.2.6. DBS Sample Testing for HCV Screening and Diagnosis

##### Advantages and Disadvantages of DBS Sample Testing

The remarkable scaling up of HCV diagnosis is dependent upon numerous parameters including HCV screening or diagnosis strategy, facilitation regarding access to diagnostic units specifically in remote areas with limited access, and among HCV-vulnerable populations [11]. The DBS utility, as a promising tool for whole blood sampling, was first touted in the AIDS epidemic for HIV type 1 (HIV-1) antibody detection [66]. Later on, the sampling tool was iterated for Abs detection against other viruses such as Ebola, Human T cell leukemia/lymphoma virus type 1, HBV, and HCV. Literally speaking, dried blood spot (DBS) sample testing is not strictly a POC analysis; however, it simplifies the collection of whole blood samples, their diagnosis, and their transportation to the testing facilities [13]. Ultimately, DBS testing has been shown to enhance HCV diagnosis and linkage to care. Furthermore, DBS sampling provides an alternate HCV sample collection procedure with two main advantages including the use of capillary whole blood through FS or heel pricking in infants and the stability of the specimen even by regular mail or courier services without the need for an intact cold chain, which can be a convenient HCV specimen preservation solution in resource-constrained settings with long transportation times and high temperatures [11]. Once received to centralized labs, DBS specimens can be stored at −20 °C or −80 °C for a long time. In parallel to that, DBS testing is also advantageous into two aspects of HCV diagnosis: first it obviates the need for phlebotomy in PWIDs and migrant IDUs with poor venous access or in premises where phlebotomy services are not available, and second, the possibility of linking HCV serological testing to reflex RNA testing for HCV diagnosis confirmation [13]. For this purpose, once DBS samples are ready for testing, the specimen is eluted off from the embedded blood drops on filter paper by using an appropriate buffer, and then the eluate can be used for HCV screening by anti-HCV Abs RDTs and/or for HCV diagnosis by RNA NAT testing [11]. The other advantage of DBS sampling is to perform reflex RNA testing on a second or third spot on the same batch of spots if the initial spot is positive for anti-HCV Abs [11]. This approach while screening and confirming HCV diagnosis on a single sample batch specimen is highly advantageous to avoid second or multiple visits for the patient for confirmatory HCV RNA testing, which is considered as a major barrier that causes many patient drop-offs in the HCV care cascade in most test settings. Similarly, DBS testing is quite attractive in off-site premises for difficult-to-reach populations, as phlebotomy is not required so testing can be performed by non-specialist staff with limited training [10]. The stored DBS samples can also be used for other HCV diagnostic algorithms such as HCV RNA sequencing or RAS testing as well as for other viral infections (e.g., for HIV and HBV) [12]. Along with the advancement and simplicity in HCV sampling such as DBS, recent studies confirm the improved acceptability of DBS over other standard HCV testing algorithms. Even though actual DBS testing is still based on the use of traditional EIA tools and a well-organized centralized lab setup to process the specimen, the ease of sample collection, transport, and storage diminishes the need for multiple central lab facilities. Furthermore, the expedited approval of DBS samples can be used as an alternate sample type for HCV diagnosis and management while using existing approved assays with DBS-adapted protocols (e.g., Abbott RealTime HCV Viral Load assay (Abbott Diagnostics, 100 Abbott Park Road, Abbott Park, IL, 60064, USA), Aptima HCV Quant Dx assay (Hologic Inc, Danbury, CT, USA), and COBAS Ampli-Prep/COBAS TaqMan HCV Test (Roche Molecular Diagnostics, Basel, Switzerland)), which may significantly impact on universal HCV screening and diagnosis campaigns in both resource-replete nations and LMICs [66].

DBS specimen testing relies on central lab settings for HCV screening and diagnosis, without the need for a second visit for HCV diagnosis confirmation. However, the results are not immediate and may require specialist consultation before final result interpretation and decision for their delivery [11]. DBS specimens are analytically less sensitive as compared to HCV fresh plasma or serum samples collected by venipuncture. HCV RNA detection is somewhat less reliable, in particular at low viral specimen titers on DBS from where a small sample volume is acquired after elution of the DBS. Furthermore, smaller blood sample volumes from PWIDs, migrant IDUs, and incarcerated people require multiple pricks to be collected, which may yield a lower HCV RNA titer and further impact the assay sensitivity. However, the assay sensitivity and result variations may depend on various factors including specimen quality and volume of collected blood, the quality of the filter paper used as sample matrices, the elution buffer, the HCV Abs and RNA detection method, sample preservation and storage conditions, and improper specimen collection, etc. [11]. Furthermore, the existing commercial assays to extract HCV Abs and RNA from the DBS samples have not been validated or received WHO PQ or CE marking, although real-world, published studies describe detailed protocols for sample collection, specimen extraction, and analysis of DBS [11]. In parallel to that, the manufacturer of the instrument or device used for DBS analysis does not provide specific instructions for their assays including processing protocols, pre-analytical treatment, and cutoffs of LOD and LLOQ. This reflects an eager need for manufacturers to develop standardized instrument protocols for regulatory approval to analyze DBS sampling such as those previously established for HIV molecular markers [66]. Furthermore, extensive elaborated studies are also required for DBS handling in different test settings including central lab, decentralized settings, or POC testing with various sample storage conditions (e.g., in extreme wet conditions for HCV RNA detection) for quality control and stringent regulatory approval (SRA).

##### Clinical Performance of DBS Sample Testing

The clinical performance of DBS collected samples for detection of anti-HCV Abs in DBS specimen vs. serum or plasma has shown excellent results in both sensitivity and specificity (generally higher than 95%). In a meta-analysis based on 19 studies of quantitative anti-HCV Abs detection from DBS specimen, a pooled estimate of sensitivity and specificity of 98% and 99% were reported, respectively, while the overall quality and heterogeneity of the study was reported as moderate [85]. In another study for anti-HCV detection by using DBS sampling, consolidated diagnostic accuracy estimates were found as: specificity 99.2%, sensitivity 96.1%, positive predictive value (PPV) 105, negative predictive value (NPV) 0.04, and with a diagnostic odds ratio (DOR) 2692.9 [86]. Until now, none of the HCV RNA diagnostic assays have been approved for use with DBS specimen on automated real-time PCR or TMA platforms, even though numerous clinical studies strongly correlate HCV RNA levels measured in DBS samples to serum or plasma samples regardless of HCV GTs/subtypes. However, in those studies, LLOQ of HCV RNA in DBS specimen was in the order of 500 IU/ mL (i.e., 2.7 logs IU/mL) which correspond to 30–50 times higher than the LLOQ in HCV serum or plasma samples, following the EASL acceptance of a LOD ≤1000 IU/mL for HCV RNA assessment [6,12]. One study reveals that HCV RNA levels in whole blood DBS specimens were lower by >1.5 logs IU/mL on average than serum samples. However, it was evident that HCV RNA could be detected in almost all DBS samples. LOD for HCV RNA from DBS samples is entirely dependent on the used NAT assay and has been estimated between 58 IU/mL to more than 250 IU/mL [12,87]. Absolute quantification of HCV RNA should not be considered and seems impractical while using DBS specimens for HCV RNA quantitative analysis. Qualitative HCV RNA analysis from the DBS specimen is reliable as the viral titer (i.e., RNA level) remains high in more than 95% of patients at diagnosis and viral relapse. In an updated systematic review, HCV RNA detection from DBS specimen demonstrated sensitivities between 80% and 100% and specificities between 94% and 100%. One study predicts the consolidated specificity and sensitivity of 98% for HCV RNA detection for both DBS specimen and venipuncture blood samples [12]. In a meta-analysis, based on an evaluation of the diagnostic accuracy of laboratory-based screening for HCV in dried DBS samples, the pooled diagnostic accuracy for HCV RNA detection was found as: specificity 99.2%, sensitivity 97.8%, PPV 44.8, NPV 0.04, with a diagnostic odds ratio (DOR) 1966.9 [87]. In another study, Aptima HCV Quant Dx assay (Hologic Inc, Danbury, CT) was evaluated for HCV RNA detection from DBS and venipuncture collected specimen. The sensitivity and specificity of the Aptima^®^ assay for HCV RNA detection from DBS samples was 95.6% and 94.1% while RNA quantification sensitivity and specificity was 100% for both sample types [88]. Hence, Aptima^®^ Quant assay effectively detects active viral infection from DBS that is clinically in proportionate to plasma. HCV cAg detection can also be used as a surrogate marker of the presence of HCV RNA before confirming active HCV infection by using HCV RNA detection assay from whole blood specimen on DBS. However, the studies decipher good HCV cAg specificity but low sensitivity for cAg detection and quantification from DBS specimens of HCV RNA positive patients (i.e., 65% sensitivity was demonstrated for one filled spot containing 50 µL of whole blood and significantly improved when two spots of the specimen were used for cAg detection and quantification).

#### 3.2.7. HCV Diagnostic Platforms in the Pipeline or Late-Stage Development

The outbreak of the COVID-19 pandemic all around the world in 2020 also halted the research and development in HCV diagnostics and validation studies (e.g., HCV self-test) as well as many non-emergency services being paused or altered [89]. Nonetheless, since July 2019, the manual of HCV diagnostics both in terms of HCV screening and diagnosis is expanding with the approval of several tests for diagnosis for WHO PQ, including the Standard-Q antibody test (SD Biosensor^®^, Suwon, Republic of Korea), the Monolisa antigen/antibody combo test (Bio-Rad^®^, Hercules, CA, USA), a portable HCV RNA test (Genedrive^®^, Genedrive Diagnostics, Manchester, UK), the Alinity-m quantitative PCR test (Abbott^®^, Des plaines, IL, 60018, USA), the RealTime HCV viral load test (Abbott^®^), and the ARCHITECT core antigen test (Abbott^®^). In addition to that, TrueNAT (Molbio^®^, Kolkata, India) was recently approved in India as a portable HCV PCR device and is currently being considered for WHO PQ. Herein, we briefly overview some emerging diagnostic tools in the pipeline or in late-stage development and their potential use and embarking role for HCV screening and diagnosis in highly prevalent HCV settings as well as in vulnerable HCV populations.

##### HCV Self-Test Diagnostics

An HCV self-test (HCVST) can be useful as a POC antibody assay for HCV screening in sexual health clinics, drug harm reduction centers, and at home to address the low coverage of HCV testing and to accelerate HCV elimination efforts in highly prevalent areas [89]. However, it would only be successful if linkage to care and HCV treatment is accessible with the patient counseling component. Furthermore, this type of testing approach may provide confidentiality and telehealth support for highly stigmatized communities (e.g., PWIDs, migrant IDUs, sex workers, MSM, homeless HCV infected individuals, and prisoners, etc.) to encourage people to get HCV tested. A large number of studies are going to be conducted in different countries to assess the usability and acceptability of HCV self-testing supported by FIND and using a testing platform donated by OraSure. Recently, an observational study was conducted in Vietnam to assess the acceptability and usability of the OraQuick^®^ HCV Self-Test (prototype) among PWIDs and MSM [90]. The study findings revealed overall high acceptability of HCVST with moderate to high usability among PWIDs (92.9%) and MSM (98.6%) in tested individuals. Similarly, the proportion of observed mistakes, difficulties, or attendees requiring assistance was fewer in MSM (33.7%, 28.8%, and 17.3%, respectively) as compared to PWIDs (62.9%, 53.3%, and 66.7% respectively; all *p* < 0.0001). In both groups, inter-reader and inter-operator agreements were found to be good (i.e., Kappa coefficient range: 0.61–0.99). However, the concordance between HCVST and the study staff-read or performed HCV testing was found to be lower among PWIDs than MSM (inter-reader concordance 88.6% vs. 99.0% and inter-operator concordance 81.9% vs. 99%). The study emphasizes enhancing support and assistance to optimize the acceptability and accelerated performance of HCVST in marginal populations (e.g., PWIDs, MSM, sex workers, homeless shelters, etc.) and for elderly people with low literacy or education levels [90].

Jiangsu Well Biotech Co., Ltd. (Changzhou, China) has developed another self-test (i.e., the Well oral anti-HCV assay) that is designed to detect HCV Abs in oral fluids with the characteristics of low cost and simplicity [91]. The clinical performance of Well anti-HCV RDT was evaluated in terms of its potential utility specifically in the areas of limited resources with limited medical personnel. The Well anti-HCV assay exhibited a specificity of 98% and sensitivity of 91.8%. The consistency between InTec (Rapid Anti-HCV Test, Xiamen, China) and the Well anti-HCV assay was found as 97% while the consistency between OraQuick and the Well assay was 97%. In addition, self-testing results were found consistent with the researcher-administered tests [91]. Currently, Kimble et al. analytically assessed a better efficiency of the OraQuick HCV diagnostic test with self-collected oral fluid [92]. Research findings depict the Well oral anti-HCV assay as user-friendly and can be used for self-testing. The assay turnaround time is 15–20 min and with proper training and instructions can be considered for use in universal screening campaigns, primary health care, and at-home settings.

##### Anti-HCV Abs Fourth-Generation EIAs

A recent study conducted in Ukraine evaluates the suitability of the INNOTEST^®^ HCV Ab IV enzyme immunoassay in paired DBS and plasma samples to screen Anti-HCV Abs [93]. The DBS samples were prepared from FS whole blood (i.e., fDBS) and venous whole blood (i.e., vDBS) of HCV-infected adults (*n* = 149) with no prior history of HCV treatment. The assay sensitivity and specificity were both 100% for samples collected on fDBS and vDBS as compared to the venipuncture plasma samples as the reference standard. The study reflects the importance of an anti-HCV serology test for use with DBS in settings where venous blood collection is not possible and to date, no anti-HCV Ab test is formally approved for use with the DBS. Another anti-HCV Abs detection assay STANDARD Q HCV Ab test developed by SD Biosensor (Korea) shows 100% sensitivity (*n* = 163; 95% CI: 97.8–100%) and 100% specificity (*n* = 320; 95% CI: 98.9–100%) in clinical diagnostic studies. The assay requires only two steps in total from sample preparation to getting results with a total turnaround time of 10–15 min. Furthermore, the assay reagents can be stored at 2–40 °C with a 2-year shelf life [94]. Table 1 compares the advantages and disadvantages of current and emerging HCV screening and diagnostic procedures with representing testing platforms.

##### HCV RNA Confirmation Assays and Tests for the Infection Cure

Some other late-stage development RNA POC assays are also in the diagnostic pipeline for obtaining WHO PQ or CE marking soon. An HCV ID Kit by using Genedrive system^®^ (Genedrive Diagnostics, Manchester, UK) has been recently launched for the detection of HCV RNA without the need for other additional equipment to enabling the testing [89]. The system detects HCV RNA from 30 µL of plasma or serum samples within 90 min without any requirement of HCV RNA extraction from serum or plasma samples. The preliminary studies reveal the assay sensitivity of 98.8% (97–99%: 95% CI) and specificity 100% (99–100%: 95% CI) while detecting HCV RNA, indicating slightly less sensitivity with an intermediate rate of 3.1%. One study conducted in Singapore showed high sensitivity (100%) and high specificity (100%) for HCV GT 3 (*n* = 240) and GT 6 (n = 16) infected patients (i.e., the highly prevalent HCV GT in Asia). The LOD for this assay is claimed to be 3203 IU/mL by the study authors [89]. Another validation study conducted in India demonstrated high sensitivity and high specificity (i.e., 100%) for HCV GT-1 and GT-3-infected patients, which remained above LOD = 2362 IU/mL [94]. The lowest LOD demonstrated for the Genedrive assay was 103 IU/mL. The assay is CE marked but has not obtained WHO PQ yet. The assay reagents can be stored at 2–30 °C with one-year shelf life. However, the assay operation requires 13 steps in total, including 5 precise pipetting steps, which require a specialist or an operator with training to perform the pipetting. Another disadvantage of the assay is that it can only accept plasma or serum samples for testing, which requires venipuncture and centrifugation of blood and several manual steps to perform the test. The latest iteration of the existing Genedrive system adds Bluetooth capability and a system for maintaining patient data with confidentiality. The user downloads an app that allows the patient and HCPs to share their result data with secrecy and the decision about the patient’s linking to care can be made locally with the care team. The assay is an ideal approach for decentralized POC diagnosis with scenarios such as “test-to-treat” and will facilitate the elimination of patients lose during follow-up visits. Although the Genedrive assay may be less sensitive, an initiative can be taken to weigh its sensitivity with a fast turnaround time of results and by surveillance of lost patients during follow-ups in vulnerable HCV populations and LMICs. Such an initiative is underway in some African countries (e.g., Rwanda, Uganda, and Kenya) by registering the Genedrive system to confirm HCV diagnosis; however, performance studies in imprisoned HCV patients have been paused due to the COVID-19 outbreak [89].

TrueNAT HCV PCR is a two-step RNA confirmation platform with the unique characteristics of a small and light-weight portable unit (5–6 kg; the larger one weighing 10 kg) with the capability of polyvalency [89]. The total turnaround time for the device is 40 min, including sample preparation tasks, around 20 min, with two or four slots in the PCR module. The smaller unit is capable to run a maximum of 6 tests per hour and 12 tests per hour can be conducted with the larger device. The clinical performance studies predict 100% assay sensitivity and specificity with a LOD of 235 IU/mL. The assay is also proclaimed for treatment monitoring; however, the assay needs to be evaluated for determining SVR rates. The system is easy to operate even by non-professionals with one or two days of training, as results are automatically displayed and may exchange between HCPs and the patient through Bluetooth wireless connectivity [89].

The modified version of the Xpert^®^ viral load assay (i.e., Xpert^®^ HCV VL FS assay) demonstrates high sensitivity (100% (93.9–100%); 95% CI) and specificity (100% (96.6–100%); 95% CI) for HCV RNA quantification with an estimated turnaround time <1 h, which may save time and can prevent patients lost to follow-ups [55]. However, the median turnaround time depends on RNA level where one study among 1426 participants found faster results for the patients with detectable HCV RNA (i.e., 32 min) vs. patients with undetectable HCV RNA (i.e., 57 min). Eighty percent of participants with a detectable HCV RNA received a result in ≤40 min. This type of testing approach could be useful for HCV RNA detection in high-prevalence settings or marginalized HCV populations including hospital EDs, PWIDs, and homeless people to reduce waiting times for an HCV diagnosis and will improve linkage to care.

Another real-time quantitative PCR assay has been approved by the FDA in March 2020 to diagnose and monitor viral load for all six HCV GTs [89]. The Alinity m HCV assay is a simplified viral load monitoring/adherence method that uses three steps for one sample and specific hybridization probes to be combined with simple RNA extraction. The assay sensitivity and specificity were found to be 100% with a LOD of 11 IU/mL, although the verified Abbott’s claim was of 12 IU/mL. The main disadvantages of the assay are to ensure correct storage of reagents to achieve sensitivity and a long turnaround time for results (i.e., 2.25 h). Furthermore, the assay system requires adequate lab equipment and sufficient space to perform testing.

##### Plasma Separation Card

COBAS^®^ Plasma Separation Card was first time touted for HIV RNA confirmation by measuring baseline levels or to monitor antiretroviral therapy (ART) in HIV-treated individuals [89]. However, the technology has been modulated and is under the late-stage development for HCV RNA confirmation and perhaps will be obtained registration approval in 2020/21. The assay LOD was found to be <1000 IU/mL (i.e., below EASL guidelines); however, Plasma Separation Card LOD has detected 866 IU/mL and even much lower (i.e., 408 IU/mL) by using an optimized assay-specific analysis package protocol [95]. The minimum sample requirement is 140 µL whole blood and collected dried plasma spot (DPS) must be dried for 4 h before packing for sample transport. The simple mail or courier service can transport the sample to the lab for up to 28 days at 18–45 °C temperature and around 85% humidity conditions. The samples at the testing place can be stored at room temperature (i.e., 18–30 °C) or 2–8 °C or −10 °C for up to 56 days. One study conducted among MSM in Amsterdam by using the COBAS Ampliprep/COBAS TaqMan (Roche^®^, Basel, Switzerland) platform for self-sample collection demonstrates that HCV (and HIV-1) RNA can be reliably self-sampled by using the DBS and can be transported to the lab for up to 21 days provided that stably stored at room temperature [96]. High sensitivity was documented both for self-sample (95.7%) and lab-based DBS sample collection (i.e., 96.4%) analysis but less sensitivity was assumed as compared to plasma samples. Subsequently, some infections can be missed and re-testing can be recommended if an individual is believed to be suffering from acute infection. HCV RNA LOD may vary in the lab from 2.2 to 3.4 log10 IU/mL and DBS LOD can be from 0.5 to 1.7 log10 IU/mL, which is more than the plasma samples. Nevertheless, DBS self-sample collection could be advantageous for diagnosing HCV infections because CHC-infected patients have viral loads higher than 3.0 log10 IU/mL. However, the patient will be needed to follow written instructions and video to self-collect good DBS to ensure accuracy and confirm hepatitis C infection diagnosis [96].

##### One-Step HCV cAg Diagnosis Platform for Therapeutic Monitoring and HCV Cure

ARCHITECT HCV CAg assay can be a substitute for HCV RNA analysis to confirm an active HCV infection both from serum and DBS samples. Although the Assay is WHO PQ and CE-marked for HCV cAg screening, efforts are underway through an abridged process by the manufacturer to achieve WHO PQ status for HCV diagnosis while submitting revised instructions for the assay used within the intended populations, the skill levels of the platform user, and which instrument to be used by March 2022 [89]. HCV cAg detection to confirm HCV cure seems less expensive in LMICs and is currently implemented as a part of reflex testing to confirm the diagnosis in 2–3 days in some countries including Georgia and Uzbekistan [89]. However, in Uzbekistan, the strategy was shifted to Roche COBAS PCR in 2020 due to shipment delays and customer service complaints. A retrospective study conducted in Canada measured HCV cAg at baseline, at weeks 4, 12, and 24 of treatment, and at the end of treatment (treated with DAAs ± PEG-IFN/RBV from 1 January 2014 to 31 March 2015) [97]. The study findings revealed good concordance (r = 0.97; *p* < 0.0001) between HCV RNA and HCV cAg at 12 weeks end of treatment; however, 3 patients out of 148 who achieved SVR12 tested HCV cAg positive at the end of treatment. False-negatives reported in the study were attributed to low viral loads. It was also evident from the study that HCV cAg levels increase over time for HCV patients with DAAs treatment failure and cAg could become positive after treatment ends. Interestingly, HCV RNA and cAg levels were found in agreement at week 24, suggesting its usability as a one-step method to confirm HCV cure. This finding is in line with the EASL 2018 recommendation that emphasizes the use of HCV cAg quantification only at week 24 SVR rates after treatment completion with DAAs [64]. Overall, HCV cAg testing is considered less sensitive and can miss infections during HCV active infection confirmation. HCV cAg DBS could be a pragmatic approach for diagnosing active HCV infection, and then sending negative cAg samples/results for RNA testing. This approach can be used in high HCV-prevalent settings instead of using Anti-HCV Abs screening or HCV RNA analysis. One study was conducted to investigate the impact of environmental factors (e.g., temperature stability) on HCV cAg assay sensitivity while using DBS samples from different blood sources (i.e., venipuncture vs. FS) and results from samples with one or two DBS [98]. The study findings projected minimal differences in assay sensitivity and sample storage temperature; however, the samples with a lower volume (3–10 fmol/L) and stored above 37 °C could affect sensitivity. Interestingly, the type of sample could not affect assay sensitivity, which was detected at about 94% for HCV diagnosis. However, larger studies and more clinical performance data both in terms of assay sensitivity and specificity are required to standardize protocols for cAg DBS collection and their stringent regulatory approval (SRA).

Lastly, the studies conducted by FIND support, with significant pieces of evidence, the use of DBS on various PCR platforms for decentralized POC testing in LMICs who are in the process of recruitment to evaluate manufacturer protocols for various platforms including Abbott m2000, COBAS^®^ AmpliPrep/COBAS TaqMan HCV Test from DBS, cobas^®^ HCV for use on the cobas^®^ 6800/8800 Systems from PSC and DBS and Aptima^®^ HCV Quant Dx Assay from DBS [99]. Table 2 enlists the WHO’s essential diagnostic list of current and emerging HCV diagnostic platforms and tests with WHO prequalification, CE marked, and that are in late-stage development.

#### 3.2.8. HCV Screening and Genotype Identification Assays

As mentioned earlier, the accurate identification of HCV genotype is a pre-requisite for physicians to prescribe appropriate DAAs regimens in certain clinical demographics of HCV affected individuals; the clinical laboratories must use accurate, robust, and short-turnaround time HCV genotype identification methods to provide in-depth information about HCV genotyping to specialists and HCPs for better patient care [100,101]. EASL also recommends the identification of HCV genotypes and subtypes and informing the clinicians while prescribing DAAs to infected patients in certain clinical circumstances (e.g., HCV GT-3-infected patients, HCV subtype 1b-infected patients, treatment failure patients with first or second generation DAAs, and RAS testing before retreatment of treatment failure patients) [102]. Interestingly, the cited literature on HCV genotype studies deciphers that the mutual concordance of two HCV genotype assays is relatively poor [101]. Furthermore, HCV genotypes and subtypes share around 95% sequence homology; that is why discrepancies have been documented between sequencing and LiPA 2.0 assay in different studies while identifying HCV genotypes/subtypes in clinical specimens [101]. One study reported the failure of the Truegene assay and the LiPA 2.0 assay with sequencing to differentiate between HCV subtypes 1a and 1b [101]. Similarly, the LiPA 2.0 assay and the Abbott Realtime HCV Genotype II assay were also found to be less efficient at identifying HCV genotype 6 sequences [101]. The studies also unveil that the LiPA 2.0 assay has certain limitations to detect HCV genotype 6 and subtype 1b genome sequences [101].

The gold standard method of HCV genotyping is still reliant on the sequencing of specific regions of the HCV genome such as 5′ UTR (untranslated region) and the structural (core, E1) and nonstructural (NS5) regions; however, the methods are time-consuming, expensive, and require the experts for sequencing analysis. Because of this, such approaches are not considered appropriate for HCV genotype analysis in POC HCV testing settings. Recent studies document the higher sensitivity and specificity of an HCV Genotyping 9G test for the detection of all six HCV genotypes in the clinical specimens [100,101,102]. The test is based on the application of 9G technology, which provides accurate information to the clinicians about HCV genotypes performed in a real-time PCR reaction at 25 °C in 30 min [100]. The testing principle is based on the meticulously designed HCV-genotype-specific ssDNA oligonucleotide probes appended with nine consecutive nucleotide base guanines (Gs) immobilized on the surface of a DNA chip [102]. The immobilized probes show high SNP discrimination ratios while hybridizing with the complementary cytosine5 (Cy5) labeled ssDNA in the PCR product [100]. The 6 HCV genotyping 9G test has shown high accuracy and simultaneous genotyping in 30 min in preliminary lab clinical studies [100]. One study conducted in HCV serum samples found higher sensitivity and specificity of the test (i.e., 99.1% and 99.7%, respectively) with the higher concordance of kappa (κ) coefficient values between the 9G test and sequencing [100]. Another study conducted in plasma samples showed 98.3 and 100% 9G test sensitivity for the detection of HCV genotype 1b and 6, respectively, whereas the LiPA 2.0 assay demonstrated a 57.9 and 71.7% sensitivity for these genotypes [101]. However, extensive studies are required in vulnerable HCV populations and mass screening programs to further validate the accuracy of the test to consider appropriate for both centralized and decentralized HCV POC testing approaches.

## 4. The Real-World Clinical Performance of Current HCV Diagnostic Algorithms

In real-life clinical experiences, the use of current HCV diagnostic algorithms has been shown their diagnostic promises to find missing millions of undiagnosed HCV infections in highly endemic areas (e.g., Egypt), from high risks HCV populations, and outreach sites with low access to HCV diagnostic facilities in the last decade and deciphered their pragmatic potential to achieve WHO goal of HCV diagnosis by 2030. For this purpose, numerous HCV testing approaches and strategies have been adopted and implemented in real-world HCV diagnostics with the inclusion of HCV RDTs, POCT, self-testing, and simplified solutions of sample collection by using various matrices (e.g., DBS). Numerous data have been collected and analyzed to interpret a steady decrease in overall epidemic size as well as to scaling up HCV diagnosis worldwide. The trajectory of current and updated HCV diagnostic data taking into account the most recent guidelines issued by major HCV professional societies predicts that by using current and emerging upcoming diagnostic approaches, the elimination of HCV is plausible, although this seems to not be possible within the time frames suggested by the WHO in many member countries. It seems impractical and is beyond the scope of this review article to elaborate on all studies deciphering the potential impact of current and under late-stage development HCV diagnostic tools on HCV elimination in real-world clinical settings. However, herein we will briefly overview some ideal HCV diagnostic strategies/policies with real-life proven clinical data which were adopted and implemented by the countries that are on the track to eliminate HCV before or by 2030. The inclusion and implementation of such diagnostic strategies can be the role model for many LMICS in their national health care plan to ‘test and treat HCV affected populations and even in some developed countries to scale up HCV diagnosis despite the availability of HCV treatment.

### 4.1. HCV Micro-Elimination Approaches in the Context of HCV Diagnosis

Two different HCV elimination approaches have been successfully tested in real-world conditions; i.e., “micro-elimination” and “macro-elimination” [103]. The developed nations and the majority of high-income countries launched micro-elimination strategies as a preliminary step toward their national HCV elimination plan. According to this strategy, the national goals of the HCV elimination plan are divided into small objectives by selecting high-risk HCV populations into groups to tailor concentrated elimination efforts both in terms of HCV diagnosis and cure. PWIDs, migrants IDUs, sex workers, MSM, individuals with HIV-1/AIDS infection, HCV/HIV or HCV/HBV coinfected patients, prisoners, homeless individuals, patients treated with hemodialysis, health care workers, patients with decompensated cirrhosis or organ transplantation, and women of reproductive age (WORA) represent to some of these key population sub-groups with a high risk of HCV transmission in any community. Targeting these delineated HCV risk groups for HCV screening and diagnosis allows faster and more efficient delivery of HCV therapeutic interventions to cure. Ultimately, micro-elimination as a ‘bottom-up” approach could be a more stable and pragmatic path to eliminate HCV nationwide in some countries [103]. Social stigma, discrimination, and criminalization are major contributing factors that impede some of these high-risk HCV populations to attend routine HCV screening and diagnosis in health care facilities. Furthermore, due to the intricacy of the traditional two-step HCV screening and diagnosis process, many of them drop off without knowing their active infection status and are lost during treatment follow-ups [103].

#### 4.1.1. Real-World Outcomes of HCV RNA POC Tests and DBS Sample Analysis in Vulnerable HCV Populations

Innovations in HCV diagnostics with the development of RDTs, POCT, and reflex HCV-RNA testing have been widely tested and implemented in some hard-to-reach, vulnerable HCV populations in real-world clinical settings. These testing approaches have been successfully evaluated in studies of PWIDs, MSM, and homeless individuals in Australia and Spain, and other parts of the world. Global data sets from 12 countries with 66,640 patients with CHC infection were analyzed to derive an optimal limit of detection for an HCV POCT for viremic infection [12]. The study findings revealed that 97% of participants with viremic infection had 1300 IU/mL or more active circulating HCV at the time of diagnosis. The study projected that as most of the current HCV diagnostic instruments may be detected as lower as 12 IU/mL of HCV, this increase in viral detection limit closer to 1300 IU/mL would maintain the good accuracy of the tests and will facilitate to developing of more affordable portable testing tools for use in LMICs.

The studies from Spain have demonstrated that POCT and DBS were 98.4% (for detectable viral loads >4 IU/mL) and 93.7% sensitive for detecting HCV RNA (i.e., >4 IU/mL), 96.7% sensitive for the quantifiable RNA threshold (i.e., ≥10 IU/mL), and 98.3% sensitive both for quantifiable RNA threshold of ≥1000 and ≥3000 IU/mL with venous plasma samples as the gold standard comparison in PWIDs [104]. Eighty percent of the participants received their HCV diagnosis results on the same day and found it very convenient [104]. Furthermore, the feasibility of self-DBS sampling at home is currently under investigation in some studies [96]. An ongoing study deciphers the usefulness of a DBS-based HCV RNA assay for HCV treatment monitoring and reinfection among PWIDs in a harm reduction center. The preliminary findings revealed 84% sensitivity, 100% specificity, and 92.6% diagnostic accuracy for the DBS-based HCV-RNA assay for HCV RNA detection at FU 12 in comparison with the RNA-POCT. The sensitivity of the DBS-based assay for viral loads detection at >1000 IU/mL was found to reach up to 95.5%. This study demonstrates the first preliminary evidence for the usefulness of DBS samples for assessing HCV cure and reinfection after DAAs treatment in the real world, facilitating treatment decentralization in PWIDS attending harm reduction services. However, with the probability of a high reinfection rate in this setting, as evidenced by some patients with low viral loads during acute HCV infection at FU12 in the study, repeat DBS testing over time will be required to identify all reinfection cases. A current prospective study compares the performance of dried plasma spots (DPS), prepared by using the Cobas^®^ plasma separation card (PSC), to plasma and serum collected from venipuncture blood of HCV infected patients in Barcelona, Spain. The study findings demonstrate the comparable performance of DPS for the serological and virological markers of HCV infection to that of the conventional specimen types, albeit the LLOD for DPS was noticeably higher in the study [105]. Another study (i.e., HepCdetect II Study) deciphers the usefulness of plasma and/or DBS to characterize acute HCV infection kinetics and transmission networks in individuals who recently inject drugs in Catalonia, Spain based on next-generation sequencing (NGS) of HCV nonstructural protein (i.e., NS5B) in 220 viremic PWIDs [106]. The preliminary findings of the study support 93.3% sensitivity of the protocol to identify acute HCV infections and 95.0% specificity with well-defined controls. HCV diagnostic interpretations by such innovative tools can also be useful to assess the performance of targeted harm reduction programs as well as to design test-and-treat strategies and to facilitating linkage to care in this key population [106]. Another study predicted the HCV-RNA testing was more feasible than DBS and showed 97.2% sensitivity and 100% specificity for viral loads >3000 IU/mL in PWIDs in real-world settings [107]. A performance in concordance with no significant difference was observed when detecting viremic infections between this one-step DBS testing strategy vs. the traditional two-step diagnostic algorithm involving venous blood samples. Another community-based one-step HCV RNA screening and confirmation strategy based on real-time PCR assay for HCV–RNA detection in DBS was validated and implemented for HIV-negative MSM and men and trans women sex workers in Barcelona, Spain [108]. The performance of the diagnostic assay was found precise, robust, sensitive, and specific. Four HIV-negative MSM were found to be recently infected with HCV. The DBS testing was found to be easy with an acceptability rate of >95%; however, no silent case was diagnosed. HCV-RNA detection in DBS samples showed good results in the study but this one-step testing approach was not considered feasible by the authors of the study in this high-risk HCV population. A recent report predicts that HIV-negative MSM diagnosed with active HCV infection has raised concerns about expanding epidemic size among MSM [24].

The evaluation of POC-RNA testing as a one-step rapid screening approach coupled with rapid (same-day) DAAs initiation in PWIDs is underway in Australia (ClinicalTrials.gov, NCT03492112). The real-world outcome of HCV RNA POCT (i.e., Xpert HCV VL FS; Cepheid) was evaluated in the ETHOS engaged study conducted in people who inject drugs in Australia [109]. The identifiable HCV risk populations in Australia are more likely among people who are homeless, incarcerated in the previous year, and IDUs who inject daily or more. Overall, 96% of participants had valid Xpert Viral Load FS POC results and invalid results were mainly attributed to early withdrawal (4%) and operator/machine error (3%). The clinical specificity and sensitivity of the Xpert HCV VL FS assay for HCV RNA detection (finger-stick) and the Xpert HCV Viral Load assay (plasma) compared with the Abbott RealTime HCV Viral Load assay in venous blood samples was evaluated in an observational cohort in Australia [55]. HCV RNA was detected in 40% of participants (i.e., 85 out of 210 eligible participants) with available Xpert HCV Viral Load testing. Both the sensitivity and specificity of HCV Viral Load Assay for RNA quantification in plasma samples collected by venipuncture were detected at 100%. Similarly, sensitivity and specificity of HCV VL FS assay for HCV RNA quantification were also detected at 100% in FS samples. The HCV VL FS assay was found to be a feasible approach to accurately detect active HCV infection from an FS blood sample in 60 min, allowing for single-visit HCV diagnosis. Currently, no assay has been registered for HCV RNA testing from DBS samples. A recent study conducted in Australia revealed the sensitivity and specificity of the Aptima HCV Dx Quant assay for HCV RNA detection in DBS samples [110]. For this purpose, 107 paired venipuncture and DBS samples from anti-HCV Abs-positive patients were analyzed for HCV RNA detection on the Aptima HCV Dx Quant and compared with Roche CAP/CTM gold standard HCV assays. The sensitivity of the Aptima assay in DBS samples was found to be 96.4% and the specificity was 95.8%. At a quantitative RNA threshold of ≥15 IU/mL in plasma, the sensitivity of the assay was predicted to be 95.1% and the specificity was 96.0%. However, at a quantitative RNA threshold of ≥1000 IU/mL (which is considered closer to the clinically relevant threshold), the Aptima assay sensitivity and specificity in DBS was found 100% [110].

A study conducted in a Swiss tertiary care hospital evaluated the diagnostic accuracy of a rapid POC HCV RNA quantification by Cepheid^®^’s GeneXpert^®^ in 100 µL capillary whole blood using lab specified standard quantitative HCV PCR test (Roche Cobas^®^ Ampliprep/Taq-man) with 650 µL venous plasma sample as the reference test [111]. The sensitivity and specificity of the GeneXpert^®^ HCV VL test were found to be 97.0% and 94.7%, respectively. All discordant results (4.1%) were attributed to DAAs treatment (i.e., week 1–4 or end of treatment). The diagnostic accuracy of the GeneXpert^®^ HCV VL FS assay with 100 µL capillary whole blood was significantly higher while depicting 100% sensitivity and 88.9% specificity. The discordant results proportion (6.1%) was found to be impacted by the DAAs treatment (at weeks 3 and 4). The study emphasizes that the POC RNA testing approach could be advantageous with same-day results to diagnose active HCV infection, monitoring treatment response, and detecting reinfection in settings with difficult venous access in daily user or long-term IDUs.

A study conducted in seven cities of South Africa evaluated the performance of POC and DBS tests for HCV Abs and HCV RNA quantification in high-risk HCV individuals [112]. A total of 471 whole blood and 218 oral fluid samples were screened by the OraQuick HCV POC test, and 218 whole blood and DBS paired samples were tested on the ARCHITECT HCV Ab (Abbott) and HCV viral load (COBAS Ampliprep/COBAS TaqMan version 2) assays. The OraQuick HCV POC testing on either whole blood or oral fluid showed an overall sensitivity of 98.5%, a specificity of 98.2%, and an accuracy of 98.4%. The DBS samples testing demonstrated a sensitivity of 96.0%, a specificity 97%, and an accuracy of 96.3% for anti-HCV Abs. Furthermore, a good correlation (*R*^2^ = 0.90) for HCV RNA quantification for DBS and plasma samples was also demonstrated by the study authors. The study findings emphasize the use of HCV RNA POC testing in high risks HCV populations while considering DBS as an alternate sample source. Both approaches can scale up HCV diagnosis and linkage to care in parts of Sub-Saharan African (SSA) nations, where decentralization and POC viral load tests are not the options [112].

#### 4.1.2. Real-World Performance of HCV cAg Assay in High Risks HCV Populations

The real-world utility of HCV cAg detection as a substitute for HCV RNA confirmation to identify active infection was demonstrated in a cohort of 744 patients in a Scottish study [113]. The overall HCV cAg assay sensitivity was demonstrated to be 82.1% and the specificity was 99.8%. HCV GT-3-infected patients were found with an increased odds of a false-negative result (OR = 3.59) and reduced odds of a false negative were significantly aligned to older age (OR = 0.92). Although HCV cAg has been implemented as a cost-effective single-step HCV diagnosis strategy in high-income countries in less prevalent HCV settings, a lot of HCV-positive individuals can be missed. Due to this, HCV cAg testing could be feasible for viral clearance determination after post-HCV treatment in LMICs where GT-3 is more prevalent [113].

The clinical performance of the HCV cAg assay in plasma samples to monitor treatment and viral recurrence in IDUs was evaluated in an Australian study (i.e., the ACTIVATE study) including participants from 17 sites in 7 countries (Australia, Belgium, Canada, Germany, Norway, Switzerland, and the United Kingdom) in an international, open-label clinical trial (ACTIVATE (NCT01364090)) [114]. The HCV cAg sensitivity and specificity were assessed for HCV GT-2/3 infection treated with PEG-IFN/RBV therapy by ARCHITECT HCV Ag (Abbott Diagnostics) assay and HCV RNA by AmpliPrep/COBAS Taqman assay (Roche Diagnostics) and was compared to the HCV RNA assay (gold standard). The HCV cAg sensitivity with quantifiable HCV RNA threshold was evaluated at baseline, end of treatment response (ETR), and SVR visits and was detected 94%, 56%, and 100%, respectively. However, the assay specificity was demonstrated to be between 98 and 100% for all time points assessed. Viral recurrence was noticed in six patients and was accurately detected by HCV cAg assay demonstrating 100% sensitivity and specificity [114].

One cost implication study conducted in Malaysia estimates the feasibility of HCV cAg as an alternate diagnostic algorithm for detecting active HCV infection among HCV-hemodialysis (i.e., HCV/HD) population [115]. HCV RNA analysis is costly in the country due to which anti-HCV Abs positive HD patients do not undergo active infection confirmation. Pre-dialysis blood was tested for HCV cAg with Abbott ARCHITECT HCV cAg platform and HCV RNA by reference method to assess the correlation between cAg and RNA as well as the cost implication of other diagnostic algorithms to confirm active HCV infection by using Anti-HCV Abs, cAg, and RNA tests. The study showed a good correlation of HCV cAg assay with HCV RNA (r = 0.943; *p* < 0.001) in terms of assay sensitivity (93.9%), specificity (99.3%), and testing accuracy (97.36%). Cost-mapping demonstrated that a biphasic testing strategy involving anti-HCV Ab as an initial screening, followed by HCV cAg for active infection confirmation on anti-HCV Ab positive and HCV RNA on HCV cAg negative patients can be considered as a modest cost-saving HCV diagnostic approach for HCV/HD patients as compared to standard diagnostic algorithms. This alternate cost-effective approach could also be relevant to HCV/HD and HCV/CKD infected patients in many LMICs to scale up HCV diagnosis and further linkage to care [115].

#### 4.1.3. Real-World Performance of HCV cAg Assay in HCV Populations with Concomitant HIV or HBV Infection

One study conducted in Cameroon (the ANRS 12336 project in Sub-Saharan Africa) assessed the performance of HCV cAg quantification in diagnosing CHC infection in participants influenced by concomitant HIV or HBV infections [76]. The HCV cAg was detected and quantified by using an automated assay (ARCHITECT HCV Ab assay; Abbott Diagnostics) in 465 HCV-Ab negative serum samples and 544 HCV-RNA positive serum samples, some of whom were infected by HIV or HBV. The HCV cAg assay’s clinical performance both in terms of sensitivity and specificity was compared to the gold standard Real-time HCV assay (Abbott Diagnostics). The HCV cAg assay sensitivity was reported at 95.7% and a specificity of 99.7% in diagnosing CHC infections. Interestingly, HIV or HBV coinfection status could not impact the overall performance of cAg sensitivity and specificity (96.4% and 96.2% respectively) in HCV/HBV coinfected patients and as well as in HCV/HIV coinfected participants (cAg sensitivity = 100% and specificity = 88.2% respectively). The second part of the project (i.e., ANRS 12,311 TAC (NCT02405013)) focusing on HCV cAg assay specificity and sensitivity as a monitoring tool for treatment efficacy instead of real-time PCR in SSA is under evaluation [76].

A multicenter laboratory evaluation study demonstrated the sensitivity and specificity of two lots of 13 RDTs (NCT04033887) at 3 laboratories using archived plasma samples from 4 countries including Nigeria, Georgia, Cambodia, and Belgium for HCV patients with or without HIV coinfection [116]. From 13 RDTs, nine were on-market products, 1 RDT with its configuration was adapted to evaluate only the HCV line, and 3 RDTs were in the late-stage development at the time of conduction of the study (i.e., prototype). In HCV positive but HIV-uninfected samples, the sensitivity of the majority of RDTs in 1 or both lots was demonstrated to ≥98% and the majority of the assay had a specificity of ≥99%. In HCV/HIV coinfected patients, specificity was recorded high but the sensitivity was markedly lower as compared to HIV-uninfected samples (i.e., only one RDT reached sensitivity ≥95%). The study also revealed that the majority of HCV/HIV coinfected samples with low assay sensitivity did not contain detectable HCV viral load or HCV cAg. Interreader variability, lot-to-lot variability, and rate of invalid runs were noticed lower for all RDTs (i.e., <2%) [116].

### 4.2. HCV Macro-Elimination Approaches in the Context of HCV Diagnosis

**Figure 5:**
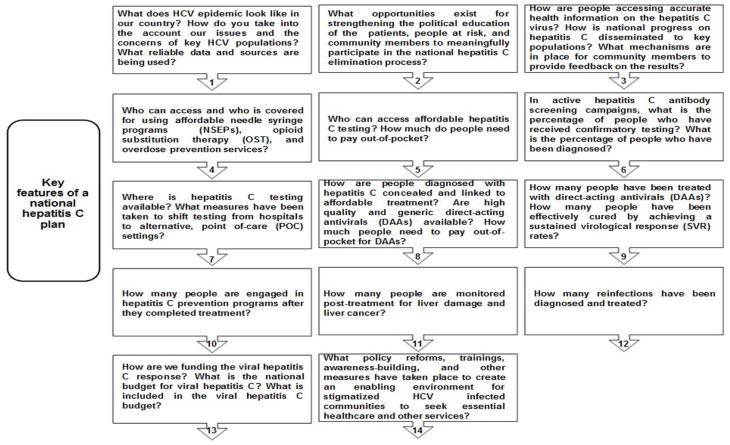
Key features of a national hepatitis C plan at a country level: The WHO member states which are off track or away to achieve the elimination goals of hepatitis C by 2030 should revise and restructure their national hepatitis C plan and reframe their national strategies to scale up HCV diagnosis, facilitating linkage to care, and speed up HCV treatment uptake. The advocacy points outlined in this figure cover all aspects of a national hepatitis C plan or strategy to overcome the barriers in terms of policy implementation and progress monitoring toward elimination goals of hepatitis C by 2030. (Source; Activist Guide to Hepatitis C Virus Diagnostics; Available online: https://www.hepcoalition.org/advocate/advocacy-tools/article/activist-guide-to-hepatitis-c-virus-diagnostics; accessed on 2 April 2021).

In contrast to micro-elimination HCV strategies, the aims and goals of the macro-elimination approach are based on large-scale HCV diagnosis, linkage to care, and treatment uptake either focusing on the entire population of the country or a large segment of it [103]. Such a comprehensive and attainable approach can be initiated with either a mass-HCV screening program, test and treat strategy, and by developing a national viral hepatitis action plan by building and involving innovative coalitions of community advocates, social society activists, researchers, and educators, legal experts, and government representatives. Three countries successfully adopted this strategy in their national hepatitis plan and implemented as nationwide or in highly prevalent HCV areas in their countries (Figure 5 demonstrates the key features of a national hepatitis C plan according to a country’s national demand and health resources) [103]. While not exhaustive, we only highlight some key aspects of this approach in terms of the use of simplified HCV diagnostic platforms with their real-life clinical outcomes to enhance linkage to care and rapid treatment uptake.

#### 4.2.1. The Georgian Experience by Initiating the World’s First HCV Macro-Elimination Program

Georgia was the first country that started the world’s first national HCV elimination program in 2015 [117]. Having a high prevalence rate of HCV infection at that time, a key strategy with strong commitment, solid cooperation of HCPs/workers, and pragmatic approaches of virus identification, linkage to treatment, and cure for an estimated 150,000 HCV-infected individuals was designed and initiated. Afterward, at the end of 2018, more than one million adult individuals (i.e., 40% of the adult population of the country) have been screened for anti-HCV Abs, and of whom 8.9% participants were found HCV seropositive [118]. Eighty percent were tested for HCV RNA confirmation, of whom 85.3% tested positive for active HCV infection with detectable HCV RNA. Around 53,000 with active HCV infection initiated DAAs treatment and approx. 49,000 completed their course of therapy at the end of the year. SVR rates were achieved in 98.5% of individuals treated with DAAs. In parallel, HCV reflex testing for cAg detection to obtain a same-day diagnosis on a single visit was initiated in the second quarter of 2018, which markedly scaled up the monthly viremia testing up to 97.5% among those that were anti-HCV abs positive. The virological surveillance data predicts that from 2015 to 2018, over one-third HCV infected individuals in Georgia were diagnosed with HCV, linked to care, and treatment. However, despite adopting this concrete HCV macro-elimination strategy at the national level, Georgia is still facing the challenge to bring the remaining individuals presumably living with HCV to linkage to care and treatment [117,118].

#### 4.2.2. Egypt HCV Macro-Elimination Approach While Initiating “Educate, Test and Treat HCV Program” and Launching the “100 Million Healthy Lives” Campaign

Egypt has the highest HCV infection rate in the world with an estimation that nearly 15% of the country’s population was infected with HCV ten years ago [119,120]. The historical epidemiological data reveals the majority of infection by the unsafe use of an injection program for the country’s decade-long fight against schistosomiasis. The demographics of a national health survey conducted in 2015 estimate that approximately 10% of the country’s population was seropositive for HCV Abs and 7% had detectable viremia. It was attributed to 5.5 million individuals with CHC infection (predominantly HCV GT-4 infection) in the general population in 2015, a significant economic and healthcare burden. To alleviate further HCV transmission and scale-up treatment for infected ones, a comprehensive national action plan was initiated by establishing a national viral hepatitis screening and treatment plan.

To achieve the realistic goal of this action plan, in the first phase, a large community-based “educate, test and treat HCV program” was conducted across 73 villages including around 200,000 villagers countrywide [119]. The importance of this HCV macro-elimination strategy was to evaluate a simplified care model which could scale up HCV testing, linkage to care, and treatment with high cure rates in rural communities of the country that had poor access to HCV diagnostics and health care centers. The key aspects of this model testing strategy were to mobilize the community for comprehensive HCV testing, linkage to care, and treatment facilitation by a network of village promoters to support the education/awareness about testing and treatment, as well as fundraising in HCV infected communities. The program was initially implemented in 73 villages between June 2015 and June 2018, and HCV diagnosis, linkage to care, and treatment was provided to all eligible individuals aged above 12 and up to 80 years old. From around 220,000 eligible villagers for viral hepatitis screening, 204,749 were screened for HCV Abs testing by using a rapid immune-chromatographic assay (InTec Products, Haicang, China), and around 34,000 anti-HCV Abs reactive cases were tested for active HCV infection by using a real-time HCV RNA PCR (Cobas Ampliprep, Cobas Taqman 48, Roche, Basel, Switzerland). Overall, the prevalence of HCV viremia was found to be 7.8%. The treatment algorithm for all confirmed HCV cases (a total of 14,495 villagers) consisted of one of several DAAs regimens following Egyptian national treatment guidelines and the 2014 AASLD and WHO 2014 guidelines for HCV GT-4 infection treatment for 12 or 24 weeks while considering the patient’s cirrhosis status (present or absent) and staging (i.e., compensated or decompensated cirrhosis). SVR rates were achieved in 98% (i.e., 14,328 patients) of all treated individuals, and treatment coverage and cure were estimated at around 84.6% of infected persons aged between 12 and 80 years. Despite the successful implementation of this community-based outreach model, it is assumed that several million Egyptians living with active HCV infection are still unidentified and require treatment. However, the number of new HCV incidences who are needed for treatment decreased to less than 5000 a month by late 2017 [119].

In the last quarter of 2018, Egypt initiated the “100 Million Healthy Lives” program with an optimistic goal to screen all Egyptians above 12 years of age or older for HCV infection and other illnesses (e.g., hypertension, diabetes, and obesity) with free consultation or treatment for those that tested HCV positive [120]. A total of 50 million countrymen (i.e., 80% of the targeted 62.5 million individuals) participated in the HCV screening program during the 7 months of the initiation of the campaign. Of those, 4.6% tested anti-HCV Abs positive, of whom 76.5% had an active infection. The WHO-approved anti-HCV Abs RDT (SD bioline HCV, Abbott) was used by finger prick with results available within 20 min. For active viremia, HCV RNA levels were quantified by real-time PCR assay (i.e., Cobas AmpliPrep/Cobas TaqMan HCV test, Roche Diagnostics). The treatment algorithm for all confirmed HCV infected patients consisted of sofosbuvir (SOF; 400 mg daily) plus daclatasvir (DCV; 60 mg daily) with or without RBV for 12 or 24 weeks duration based on the patient’s cirrhosis status (present or absent) and staging (i.e., compensated or decompensated cirrhosis). SVR rates were achieved at 98.8% in treated individuals. With strong political will, active participation of the HCPs, and a transparent negotiation policy with stakeholders, the total costs of the HCV testing and treatment amounted to only USD 207.1 million. The price negotiations led to a screening test cost of USD 0.58/test, USD 4.80/HCV RNA confirmation test, and USD 85 for the generic DAAs combination (SOF/DCV) for 12 weeks of treatment. The overall cost to identifying an individual with HCV viremia was estimated at USD 85.41 and the total cost of HCV identification and curing a patient resulted in USD 130.62 [120].

#### 4.2.3. The US HCV Elimination Approaches in the Context of HCV Diagnosis

In the US, many HCV infections cluster geographically, with higher prevalence rates in some specific regions customized with significant socioeconomic, racial/ethnic, and educational disparities seen in both rural and urban areas [10]. In such areas, community-based HCV screening and linkage to care interventions that are geographically focused can be useful to identify undiagnosed HCV people and overcome challenges to cure. This approach coupling targeted outreach with patient navigation can be promising to enhance HCV screening and linkage-to-care efforts. The project ‘ECHO’ and ‘Check Hep C’ are the two model examples in this scenario, where the former one provided access to HCV care in remote areas with severe shortages of specialty health care providers (HCP) and in prisoners. The latter model aimed to scale up HCV screening and linkage to care in sites located in low-income territories with high prevalence rates of HCV infection and to serve high risks and hard-to-reach PWIDs. The program aimed to use Rapid POC-HCV Ab screening, on-site phlebotomy for active HCV viremia detection, linkage to care via patient navigators, and telemedicine to deliver DAAs therapy. According to the program, HCV Abs screening was conducted in 4751 individuals representing a diverse and at-risk cohort of 49% Hispanic, 40% Black non-Hispanics, 41% experienced prior incarceration, 25% with prior IDUs, and 15% homeless people. The program identified 11% (n = 512) confirmed HCV cases, 85% of whom visited at least once for a follow-up appointment. These findings reveal that the implementation of such innovative HCV screening models can target hard-to-reach, high-risk HCV populations by using decentralized testing approaches and by patient navigation to increase HCV screening and linkage to care [10].

Second, Several academic research groups in the US have initiated several programs based on the educational interventions and data feedback to providers, and to a lesser extent patient navigation to scale up HCV screening and linkage to care [121]. The investigators in New York City tested the impact of this approach on the rates of HCV screening among baby boomers in two primary care practices. It was revealed that automated medical record alerts were not found so much effective. HCV screening rates were found to increase from 55% to 75% (*p* < 0.01) during the study period, and this improvement was more attributed to the physician trainees than among the faculty. 84 HCV-positive patients were identified during the study period, and 60 of whom completed an appointment with a hepatologist, and 32 initiated treatment [121].

Another study from the University of Michigan describes the impact of an ‘electronic health record (EHR) best practices alert’ on rates of HCV screening and linkage to care among baby boomers at 13 clinics [121]. According to this model, a soft alert linked to educational materials, an order set, and prompt access to specialty care was sent to the patients with a repetition at 10-months intervals if initially ignored. In comparison to the practices conducted six months before the alert, it was found that the HCV screening of eligible individuals increased from 7.6% to 72% (*p* < 0.001) during the first year afterward, and the number of confirmed HCV cases escalated from 23 to 53, of whom 31 had received a prescription of DAAs regimens, and 20 initiated treatment. Both of these studies validate the effectiveness of educational interventions to simultaneously increasing awareness about HCV screening and linkage to care cascade [121].

## 5. Real-World Challenges to HCV Diagnosis

The rapid pace of innovations in current HCV diagnostic algorithms and their real-life utility has raised the hopes to achieve the WHO set goals of HCV diagnosis (i.e., >80%) by 2030 [8]. In parallel to that, the real-world effectiveness of pan-genotypic DAAs while achieving SVR rates more than 95% to treating all six HCV GTs-infected individuals with different clinical demographics (i.e., treatment naïve or treatment-experienced patients, HCV GT-3-infected patients, virologic failure/viral relapse patients with first, second and third generation DAAs therapy, HCV/HIV, HCV/HBV, or HCV/CKD coinfected patients, and post-liver transplant patients, etc.) and in HCV-associated hepatic comorbidities patients (e.g., cirrhosis and advanced fibrosis) have also skipped the need of pre-genotyping testing and HCV viral load evaluation at weeks 4–8 post-treatment and in some cases after post-treatment completion or HCV cure. However, challenges remain in HCV diagnostics to implement universal anti-HCV screening and diagnosis strategies as well as gaps needed to be filled while streamlining confirmed HCV-positive patients to the linkage of care and ultimately cure. The barriers to HCV diagnosis and care access differ from country to country and can be identified, discussed, and managed by designing the strategies according to the country’s health care plan, available resources, and local contexts. Herein, we will briefly overview critical challenges in front of HCV diagnosis and some important gaps in terms of policy and progress toward their implementation in real-world clinical HCV diagnostic settings. We will also elaborate the broader efforts to use innovations in HCV diagnostics to enhance HCV testing across high prevalent areas, in health care systems, and outreach sites in remote areas with limited access to testing and treatment centers. We will also focus on national HCV plans and policy interventions of some countries, those who are on the track of HCV elimination before or by 2030, by leveraging the lessons for the strategy designing in many LMICs to specifically improving HCV screening, treatment uptake, treatment adherence, and cure.

### 5.1. Diagnostic Barriers at the Patient Level

#### 5.1.1. Social Determinants of Health

Social stigma, discrimination, marginalization, and criminalization represent some of the most significant barriers for the high risks and hardly reached HCV-infected populations (e.g., PWIDs, IDUs, migrant IDUs, MSM, sex workers, prisoners, and homeless persons) for HCV screening [122]. In most nations, the criminalization of drug use and discrimination of a spectrum of gender and sexual identities (e.g., LGBTQ+; an umbrella acronym for lesbian, gay, bisexual, transgender, queer/questioning) make targeted, risk-based testing among vulnerable HCV affected populations extremely challenging [123]. Once, anti-HCV screening for high-risk populations were included as a prominent feature of international HCV diagnosis guidelines. However, strategies to identify specific risk factors for HCV transmission predominantly based on risk behaviors or exposures could not be successful because of the patients’ reluctance to disclose those risks having a fear of social stigma and limitations of the HCPs in collecting risk information [13]. Worldwide, 40% of HCV-infected individuals represent recent IDUs and 9% of people living with HCV are those who recently injected drugs [13]. According to this ratio, HCV prevalence is estimated to be 52.3% among the 15.6 million people who inject drugs. However, to provide harm reduction services, less than 86 countries have NSEPs and OST programs of varying levels [48]. For this reason, PWIDs are always considered as a priority population for HCV elimination strategies both in developed nations and in themiddle-to-low income countries, with some variations. It has been predicted that 43% of all new HCV infections could be prevented over 12 years (i.e., from 2018 to 2030) if the HCV transmission associated with PWIDs can be removed over that period [8,13]. Drug use is also widely criminalized all around the world; however, IDU is still being practiced in 179 of 206 countries worldwide [48]. It is likely that the criminalization of drug use could not deter its use very significantly and is not an effective public health strategy for curbing the global burden of the HCV epidemic. Another factor that cannot be undermined is the treatment uptake in this population, which is historically low [10]. The key challenges for HCV screening in this population mainly pertain to lack of engagement with traditional sources of health care including linkage to treatment and cure.

Seventy-one countries worldwide have criminalized homosexuality, and homosexual activity can result in the death penalty in 13 of these countries [48]. This risk may prevent or make it nearly impossible for many hundreds of thousands of MSM and transgender individuals to access HCV diagnosis and treatment services for HIV, HCV, and other sexually transmitted infections (STIs). Although the criminalization of sex work is law-protected and pervasive in the majority of countries, this social stigma also prevents the sex workers’ access to HCV diagnosis and essential healthcare services and legal and other health-related consultations. HIV patients living with HCV without knowing their active infection status also represent a high-risk HCV transmission factor with an estimation of 2.3 million people are living with HCV/HIV coinfections worldwide [13]. HCV screening in such a population is not easy and even most of the screened individuals avoid follow-up visits for their diagnosis results and during treatment uptake and cure monitoring [48].

The seroprevalence of HCV is estimated to be 16.1% for US prisoners, with 10.7% having active viremia upon RNA testing [10]. In Canada, more than 50% of people who are incarcerated (PWAI) have a history of drug use, and around 76% of PWIDs have a history of incarceration [124]. Both identifiable risk factor populations are responsible for the higher prevalence of HCV among PWAI as compared to the general population. Anti-HCV antibodies have been detected both in indigenous men (27.7%) and women (44.8%) who are incarcerated. Interestingly, incarcerated women have a higher prevalence of anti-HCV antibodies as compared to men. Moreover, 25 out of 1000 PWAI who are in federal custody without prior HCV exposure are at risk per year to infect with HCV. Likely, PWIDs’ low HCV screening rates have also been demonstrated in inmates because of several factors [124]. Although an “opt-out” HCV testing strategy for screening all prisoners is recommended, the prison system of only 13 states of the USA has routine HCV screening, and only 40% of jails reported routine HCV screening in a 2012 survey [10]. Furthermore, the correctional systems have a disincentive to detect HCV infections by facing the difficulty in linking inmates to care and due to the high prices of DAAs [10].

The prevalence of HCV Abs has been demonstrated to be 0.1 to 3.6% worldwide in pregnant women [26]. Some studies suggest that CHC-infected pregnant females could face adverse neonatal outcomes and vertical HCV transmission could be a risk factor for HCV mono-infection in up to 5% of cases with a common source of HCV infection in children [26]. This population is also facing a unique challenge for risk-based screening in many LMICs, which is poorly implemented by clinicians and inadequately sensitive over routine screening of pregnant women for HCV infection with significant advantages in developed and high-income countries. Interestingly, despite the updated guidelines of AASLD and IDSA in 2018, and further recommendations of CDC in 2020, which recommends universal HCV screening with each pregnancy (except in setting where HCV prevalence is <0.1%), most pregnant females in the developed nations (e.g., in the USA) may not want to be screened for HCV because of social stigma, involvement of state agencies to snatch their kids as a result of being IDUs, and the criminalization of drug use during pregnancy [10]. Furthermore, linkage to care is also low in pregnant females such as PWIDs, and postpartum treatment completion rates are even lower. Furthermore, HCV treatment with DAAs is only recommended after breastfeeding is completed. Many mothers also lose health insurance during the postpartum period and may be lost to treatment follow-ups [10].

**Figure 6:**
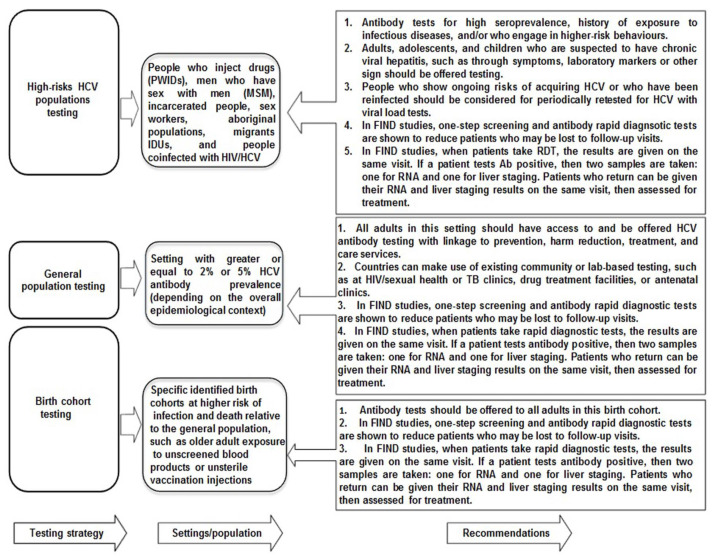
WHO guidelines focusing on three testing strategies for the screening of new HCV incidences in countries with high risks HCV populations, with anti-HCV Abs prevalence greater or equal to 2% or 5%, and birth cohort screening. (Source; Activist Guide to Hepatitis C Virus Diagnostics; Available online: https://www.hepcoalition.org/advocate/advocacy-tools/article/activist-guide-to-hepatitis-c-virus-diagnostics; accessed on 2 April 2021).

In the US, 50% of all reported HCV infections belong to individuals born between 1945 and 1965. For this reason, one-time HCV screening has been recommended in this birth cohort [10]. HCV screening rates are still low in this population, not only because of other predisposing factors but also involving stigma associated with HCV infection, the asymptomatic course of the illness from acute to CHC infection, lack of awareness about testing recommendations, and low health care engagement of the most at-risk populations. CDC guidelines now recommend one-time, routine, opt-out HCV testing for all individuals aged 18 years and older to broaden HCV screening in birth cohort settings as well as to all pregnant women, except in areas where HCV prevalence is <0.1%. In the general population, HCV testing can be initiated where HCV seroprevalence is intermediate (≥2%) or high (≥5%) [3,10]. (Figure 6 demonstrates the WHO HCV testing strategies for new HCV incidences in high risks populations, general population, and birth cohort screening).

#### 5.1.2. Plausible Solutions to Overcome Social Determinants of Health during HCV Diagnosis

##### By Raising Awareness about Social Determinants of Health

To scale up HCV screening and diagnosis to the above-mentioned high-risk, disproportionally affected, and harder-to-reach HCV-infected populations, the social determinants of health must be improved by reducing the stigma and discrimination among decriminalizing communities most affected by HCV. This can be accomplished by implementing and enforcing anti-discrimination and other suitable legislation that protect rights for everyone to test and treat. In Georgia, representing the first country in the world to initiate a national HCV elimination program, the diagnostic advocacy workshop participants pointed out several barriers to HCV testing and treatment including internalized patient stigma, the stigma associated with HCPs towards patients from key affected communities, and lack of patient confidentiality by medical record keepers [48,103]. To surmount these hurdles, stakeholders of the national HCV elimination program suggested workshops and informative seminars that can build awareness among community individuals on legal protections against discrimination and social stigma. Similarly, in Malaysia, to expand and facilitate decentralization of HCV testing and treatment, the stakeholders of the healthcare system in collaboration with civil society organizations developed learning modules and a curriculum on social determinants of health (e.g., social stigma and discrimination) and integrated those as part of medical and community health education activities [48]. Systems can be developed by establishing community-friendly, peer-led healthcare services and procedures that may be adapted to track and confront the stigma and discrimination associated with HCV screening in vulnerable HCV populations by trained and sensitized HCP with a check to be penalized if found to be discriminating and denying services.

##### By Introducing Simplified HCV Diagnosis in Stigmatized HCV Populations

To address HCV screening and diagnostic gaps among high-risk-exposure individuals where multiple visits and follow-ups with traditional two-step HCV diagnosis process are always a particular challenge, novel diagnostic tools with rapid POC tests have been developed and successfully implemented in various settings. The fast turnaround time (~20 min) with rapid POCT and reflex HCV RNA testing to minimizing diagnostic steps could be a feasible diagnostic approach in such populations [13,15]. Further transformational innovations in reflex RNA testing with DBS and DPS sampling, POC-RNA testing as one-step RDTs, and DBS self-sampling could also be implemented in high-risk HCV populations in outreach rural areas to alleviate the social stigma, discrimination, and racial disparity of HCV screening (Figure 1(1a)). Although not a matter of discussion of this review, patient drop-off after HCV diagnosis has also been demonstrated significantly higher in PWIDs, sex-workers, MSM, and homeless populations while linking to care because off-site referral to subspecialty care seems to not be effective in such populations [15].

##### By Implementing Decentralized HCV Screening Policies for the General Population

The birth cohorts and high-risk population screening would not be effective to find the missing millions infected with HCV because of the variable global epidemiology of HCV infection [8]. Simplified screening policies with a broader impact on regional strategies will be needed to achieve the WHO’s HCV elimination targets. In this scenario, health care authorities may opt for general population screening programs for individuals seeking driving licenses or visas, for students enrolling in college/university admissions, or for those joining the armed forces [48]. However, this approach also overlooks most affected HCV communities and may miss positive infections. Universal anti-HCV screening of all adults can be a pragmatic, goal-oriented, and deep-impacting strategy to eliminate the HCV epidemic worldwide [15]. As demonstrated earlier, Egypt successfully embarked on one such strategy to screen all adults aged 18 years and older, and nearly 2 million have to be treated by the end of 2018 [120] (Figure 1(1b)). Although this type of strategy can be too costly in premises with low HCV prevalence rates because a large number of individuals are needed to be screened, the modeled studies in France and the US have shown that universal HCV screening could be cost-effective in low prevalence areas [48]. Alternatively, random selection or using a ‘hub-and-spoke model’, as tried in Italy, could be a practical compromise between universal and targeted HCV screening (Figure 1(1b)).

### 5.2. Diagnostic and Healthcare System Barriers

An ideal care pathway for HCV-infected patients will involve accurate diagnosis, linkage to care (i.e., pretreatment workup), and treatment uptake/initiation in a single day. To achieve this, healthcare systems must have a deeper understanding of HCV prevalence in the general population as well as in vulnerable communities [48]. Furthermore, the facility of easily available and more affordable HCV diagnostic tests with streamlined procurement and social support for patients in terms of prevention and harm reduction counseling, transportation assistance, and other social services are pre-requisite. Some studies support this assumption with the evidence that while increasing HCV screening and diagnosis rates will not have a significant impact on WHO elimination targets without a steady improvement in linkage to care [8]. Because of this, many patients lose follow-ups during HCV screening, diagnosis, and even treatment uptake and post-treatment evaluation. Studies from Europe and the US have shown that 69% and 47% of HCV-screened patients did not follow their confirmatory HCV diagnosis tests, respectively [13]. In contrast, Australia and France have higher HCV diagnostic rates (i.e., 75% and 74%, respectively) while adhering to their national screening plans. These studies leverage the lessons to facilitate healthcare centers with innovative, cost-effective, robust, and short-turnaround time diagnostic techniques as well as cultural competency of the HCPs to ensure accurate HCV diagnosis and improved retention of the patients in the care pathway. The current care pathway can be visualized up to 10 single visits steps from HCV screening to cure, as advocated by international guidelines for HCV management, including AASLD/IDSA, EASL, and WHO (Figure 2) [13,15]. These steps can be configured into three distinct phases: HCV screening/diagnosis, linkage to care/pretreatment, and treatment uptake/treatment monitoring including post-treatment follow-up [15]. In many settings, HCV diagnosis involves positive anti-HCV Abs screening which further goes on to HCV RNA testing (i.e., active infection confirmation), known as reflex testing; however, this is not provided in every HCV diagnostic setting with a testing platform. After that, the pretreatment phase for many HCV infected patients include an initial visit to a specialist for pretreatment assessment with the recommendation of a liver staging test (e.g., APRI, FIB-4, FibroScan, and other tests, etc.) to determine liver fibrosis and hepatic cirrhosis staging and to identify viral and host factors, which may impact treatment choice, prognosis, and or post-treatment follow-ups [15]. With the availability of pan-genotypic DAAs proven highly effective against all HCV GTs/subtypes, some assessment tests of the pretreatment phase have been reduced; in particular, HCV GTs identification and RAS testing for HCV drug resistance [11]. However, it is still recommended to assess the other coinfection status such as HBV, HIV, and CKD in HCV-confirmed patients as well as HCV GT-3 identification where appropriate and to choose the right DAAs combination for treatment [12]. In regions where access to pan-genotypic DAAs is restricted and the treatment is not affordable, the numerous steps to HCV diagnosis, linkage to care, and test-of-cure (at week 12 or 24 post-treatment completion) are very intricate for the patients and ultimately result in their loss to follow-up visits.

#### 5.2.1. Advantages and Limitations of Centralized and Decentralized HCV Diagnostic Platforms/Settings

Currently, most HCV diagnostic services are provided both at central and decentralized locations while using various screening and diagnostic platforms in developed nations, middle-income countries, and many LMICs. It is a well-documented fact that both services are integral to scale up HCV diagnosis despite their pros and cons at community clinics, hospitals, and outreach open settings. Furthermore, no single approach including a sole diagnostic product or testing tools can reach all populations affected. Centralized HCV testing platforms are critical for regional surveillance programs to monitor the progress of the tasks toward elimination goals whilst providing patient management as well [10,11,12,13,15]. Furthermore, such approaches could be advantageous for the management of virological surveillance data and oversighting for quality assurance of tests used for HCV screening and diagnosis. In centralized testing, clinics and small labs rely on central lab infrastructure to analyze the samples and interpret the final diagnosis results. Such set-ups have certain roles to scale up and streamline diagnosed HCV people to linking to care [66]. However, when anti-HCV Abs RDTs and HCV RNA confirmatory testing is only available at central labs, it represents a costly barrier to many patients as it takes a lot of time and sometimes requires patients to travel great distances in remote areas [66]. In high risks HCV communities (e.g., PWIDs, sex workers, homeless, MSM, migrants IDUs), central lab HCV testing is a predisposing factor for many patients’ loss during HCV confirmatory testing, pretreatment assessment, and post-treatment follow-ups [10,66]. Furthermore, in LMICs, HCV screening and diagnosis based on central lab testing become more challenging for the individuals to take off a day’s work, arrange for childcare, and travel long distances [48]. Studies from high-prevalence settings predict that relying only on a centralized HCV testing and treatment cascade creates a bottleneck and puts a huge burden on healthcare resources, staffing, and infrastructure [66]. Furthermore, centralized testing requires strong sample transport and result delivery networks (e.g., mail, courier, etc.), which may delay the time that consequently increases the number of patient visits and likewise patient loss along the care cascade [11]. The general assumption is that adding steps to HCV diagnosis adds more net costs. In contrast, decentralized testing brings HCV diagnosis and treatment together at the point of care where most people are receiving their health care services (e.g., harm reduction centers, NSEPs, mental disorder clinics, etc.) and can retain more people in the HCV care cascade [66]. It may also alleviate the health disparity among populations that are disproportionally affected by HCV and can escalate access to HCV diagnosis and care. HCV samples collection from decentralized POCs platforms and sending them to the centralized lab for tasting has also been shown to be an effective way to scale up HCV diagnosis and linking people to care in highly prevalent HCV communities [48].

#### 5.2.2. Potential Ways to Overcome Health System-Related Obstacles to Simplify HCV Diagnostics

##### Shifting Centralized Diagnostic Approaches to Complete or Partly POC Testing Approaches for Vulnerable HCV Populations

Several models exist for minimizing the steps to confirm HCV diagnosis and to initiate treatment as early as possible in real-world clinical settings. A ‘consolidated HCV care pathway’ based on only two visits for patients to diagnose HCV infection and receive treatment was evaluated with significant outcomes in terms of reducing patients lost to follow-up from screening to treatment in the USA [15]. Furthermore, integrating HCV care into existing health care programs for other infections (e.g., HAV, HIV, HBV, tuberculosis, and malaria) and services are also bearing good fruits in some European countries as well as in high-income countries in this context [48]. The demands are growing in high-risk HCV communities (e.g., PWIDs, sex-workers, MSM, inmates, homeless, and migrants IDUs) to move away, at least partially, from HCV centralized lab facilities to offer HCV testing more rapidly at POC settings where such communities receive their routine health services (harm reduction centers or sexual health clinics) [13]. In this scenario, anti-HCV Abs screening is more feasible to skip in those populations and start HCV diagnosis with POC confirmatory testing not only to prevent the loss to follow-ups but also to accelerating HCV treatment uptake (Figure 1(1a)) [15].

Oral fluid or FS capillary blood collection onto filter paper as a DBS can facilitate decentralized diagnosis of HCV in most vulnerable populations (e.g., PWIDs, migrants IDUs) with poor venous access which is considered as a significant obstacle for standard phlebotomy [15]. Capillary FS blood on DBS can also be adopted to increase access to centralized HCV testing among PWIDs or those in remote populations. The studies are ongoing in Holland and Australia (i.e., NCT02102451) to evaluate the potential significance of self-collected DBS samples as a tool to scale up HCV screening, confirm HCV viremia, and to monitoring reinfection among those at high risks [10,96]. HCV sampling by FS for POC HCV RNA testing in those territories is already providing an opportunity for immediate results and treatment uptake to the patients.

##### Minimizing Patient’s Visits up to Four or Less in HCV Care Pathway from Diagnosis to Cure in LMICs

Significant and challenging barriers exist in many LMICs where two-thirds of people are living with active HCV infection status and tackle a lot of obstacles to access HCV testing and treatment from poor healthcare infrastructure and inadequately developed HCV diagnostic platforms [10]. In those settings, patient visits can be reduced up to four visits by conducting HCV Abs screening and POC RNA tests along with pretreatment assessment in one visit, followed by a second visit to receive results, consultations with HCPs, and receiving treatment prescription for generic pan-genotypic DAAs. The third visit could only be confined to SVR12 testing for treatment completion, and the fourth one can be fixed only for patients who might be at risk of reinfection, viral relapse, and monitoring for hepatic cancer [15]. Although, the gaps still exist in this approach to follow with the chances of patients drop-off and lost to follow-ups and even to the accessibility of pan-genotypic DAAs at the lowest costs; nevertheless, it could be more practical than reflex HCV RNA testing which is still not affordable and laudable to implement in many LMICs. Integration of other services (e.g., HAV/HBV/HIV testing, counseling, and vaccination, pregnancy and family planning, harm reduction services, and referral for mental health disorders and other services) with HCV testing may put a huge financial burden on their health infrastructure and cannot be provided to everyone under one umbrella of healthcare centers in many LMICs [48]. However, some services can be integrated into polyclinics acting as ‘one-stop shops’ to receive patients’ diagnostics and treatment services in one visit. Some real-world clinical studies (e.g., in Cambodia) have been shown the significance of simplifying the HCV diagnostics pathway with an increased number of individuals who were screened, confirmed as HCV-positive patients, and initiated treatment [48]. Subsequently, by simplifying the diagnostic pathway and model of care, the estimates predict that nearly all patients in Cambodia retained in care until tested for SVR12 as a confirmation of cure.

##### One-Stop Shops for HCV Diagnosis and Treatment Rather Than Decentralized Testing Approaches

As previously emphasized, decentralization brings HCV testing closer to people wherever they get services and provides opportunities to link HCV-positive patients into care in highly prevalent communities. However, it is entirely based on the availability, affordability, and quality of HCV diagnostic tests and lab/health systems infrastructure. Several models of care are practically being explored to expand decentralization both in urban and rural/remote areas. Polyclinics as ‘one-stop shops’ in urban areas, the integration of multiple health care services along with HCV screening in primary health care centers, as well as mobile microbiological lab units in remote areas are some notable examples in this regard [15,48]. Other healthcare platforms which could be intermingled with HCV’s decentralized testing approach include community health clinics, NSEPs, clinics for OAT and OUDs, incarceration centers, homeless shelters, pharmacies, youth clubs, and mobile units for community-based HCV screening and linkage to care [48].

##### Telemedicine and ECHO Models to Expand Decentralized HCV Testing

Telemedicine and the ‘extension for community health outcomes (ECHO)’ models could be used to reshape HCV health care delivery in many LMICs, in particular, those which are geographically isolated and have underserved healthcare populations [10]. The ECHO model provides training to HCPs and educators in HCV, using web-based software. Clinical knowledge related to managing patient health outcomes through ‘teleECHO’ clinics and case-based guided practice is brought to the physicians who treat HCV patients. Patients’ surveillance data, treatment outcomes, and program cost-effectiveness are monitored through centrally collected data with access to HCPs and resources. An ideal healthcare system’s strength cannot be measured solely by its capabilities of providing diagnostic and treatment services to the communities but also by the oversight of strong surveillance, therapeutic monitoring, and efficient reporting system. In a true sense, the ECHO model builds the capacity of non-specialist physicians and auxiliary HCPs to treat and manage HCV patients, complement decentralization of HCV testing, and improve HCV treatment uptake (Figure 1(1a)) [10].

##### One-Sample, One-Test, and One-Step HCV Diagnostic Approach

The one-sample strategy either by using DBS samples for anti-HCV Abs screening or HCV RNA analysis and one-test strategy (e.g., most probably HCV cAg assay) both for HCV screening and diagnosis could be a ‘holy grail’ in highly prevalent HCV settings and in many LMICs to improve access to early HCV diagnosis, enhancing treatment uptake and reducing patient loss to follow-ups. However, their real-world implications, utility, and effectiveness would be based on healthcare setting/infrastructure, epidemiological context, and affordability [15,48,66,103]. Perhaps an interesting alternate rather than to this one-test strategy could be a biphasic strategy involving first an anti-HCV positive Abs screening with HCV cAg assay and reserving HCV RNA analysis only for those patients who are anti-HCV Abs positive but are found to be HCV cAg negative [11]. This approach was tested in a study conducted in Canada to evaluate viral relapse in HCV-treated patients with DAAs. The study findings demonstrated that the use of HCV cAg assay eliminated the need for HCV RNA tests in more than 75% of cases. Another study signified the cost-effectiveness of this biphasic strategy in highly prevalent HCV communities in LMICs [15].

##### Task-Shifting Rather Than a Specialty in HCV Diagnosis and Treatment

Decentralized HCV diagnostics require massive training, budgets, and capacity building of a wide variety of HCPs. To surmount this burden continued medical and community health education and awareness must be included in national HCV programs. International guidelines for HCV testing and treatment must be simplified to transfer the service delivery burden to auxiliary health care professionals (i.e., task-shifting) to make them more efficient and expert in HCV care management [48,103]. Task-shifting in the HCV care cascade can only be tailored according to a country’s need and should occur along with other strategies to escalate the numbers of skilled healthcare staff through expanded educational and training programs at national levels. Furthermore, policies and regulatory changes related to shifting RDT testing to auxiliary health care staff, through training, reporting systems, and lifting restrictions to prescribe DAA regimens to non-specialists should be fast-tracked [8,48,103]. The procedures and protocols related to standardized medical certifications required for administering HCV care by additional healthcare workers, as well as for clinical monitoring and supporting supervision, should be regularly provided. People living with HIV/HCV and individuals with lived experience (e.g., peer educators) should be consulted and included in policy designing and decision making about the HCV care pathway through their experiences [48]. This segment of a country’s population can play an integral role in scaling up HCV diagnostics and may push the number of people to be cured.

### 5.3. Regulatory and Licensing Barriers

The pioneers to design and developing HCV RNA diagnostic platforms to confirm diagnosis along with the latest advancements in the tests or assay performance are only a few companies worldwide. Abbott, Roche, and Cepheid are the only giant distributors of POC RNA viral load or lab-based HCV cAg devices and testing reagents in the world [48]. The non-existence of any competitor of those companies in a country creates monopolies and cartelization that increase the prices of their testing platforms sky-high and also limit the supply of the testing kits/consumables required in certain conditions. In addition to that, high fees are levied upon for equipment maintenance, system repairs, and sample cartridges. Furthermore, sample cartridges are specific and only fitted in their testing platforms and ultimately prices are charged from the countries to supply. By analogy, some testing platforms are only available to diagnose specific viral infections and other diseases (e.g., HIV, HBV, and TB) depending on their procurement and funded by donors. Although these companies have multiplexing testing platforms, the exclusive licensing of those platforms permits the countries to perform only HIV, HCV, and TB testing [48].

#### 5.3.1. Advantages and Limitations of the WHO PQ and CE Marking for HCV Diagnostic Platforms

Being a high-risk in vitro diagnostic (IVD), an HCV diagnostic platform, screening, or diagnostic assay must undergo a rigorous and comprehensive assessment including diagnostic performance (both in terms of sensitivity, specificity, and platform/assay efficacy) in clinical samples obtained from the population in which the diagnostic platform/test/assay intended to be used, an independent assessment/evaluation of the product in laboratories assigned by stringent regulatory authorities (SRA), as well as a site inspection of the manufacturing unit to assess compliance with good manufacturing practice (GMP) [66]. For HCV-IVD platforms and tests, the WHO prequalification (WHO-PQ) process is one regulatory standard that ensures the quality, safety, and efficacy of IVDs suitable for their intended use in LMICs [48,66]. It also plays a standard role to help international procurement agencies and countries with resource-constrained settings and limited domestic regulatory frameworks to consider and procure diagnostic devices of high-quality standards for their national disease elimination programs and to obviate the chances of duplicative regulatory approval procedures [48]. The US FDA and Conformite Europeenne (designated as CE or CE-IVD) are also two other standards for IVDs. Data supporting the registration procedures of IVDs to US FDA and WHO-PQ are available on their websites; however, this is not easily accessible for medical devices to the European Databank and access is authorized only to competent national authorities [48]. It creates an unintended obstacle to the rapid identification of CE-IVD marked assays. The high fees of PQ assessment (i.e., USD 5000–12,000/product and an annual fee of USD 4000 to keep product on WHO PQ list) also keep small markets away from being part of this process [48]. Although the WHO-PQ review process ideally takes less than one year, the lengthy periods attributed to inquiring auxiliary manufacturing information and technical communication adds extra time to the process. Consequently, the manufacturers waiting for WHO PQ lose their sales during the process with reduced cash flow and ultimately quit from the process or enter into the market later than planned. It is also worth mentioning here that studies supporting the SRAs for HCV diagnostic platforms are generally based on data collected from laboratory settings and have limited applicability to other HCV-infected populations with GT-4, 5, and 6, as well as exempt from providing diagnostic efficacy data of HCV-tested populations coinfected with HIV or real-world clinical use [48,66]. Unlikely, few WHO PQ and CE-IVD marked diagnostic platforms for viral load test and HCV cAg assay are validated in real-life clinical diagnosis and perhaps in many LMICs where HCV diagnostic procurement is majorly through Global Fund are even limited to these.

#### 5.3.2. Designing of Simplified Regulatory Procedures for the Approvals of HCV Diagnostic Platforms in LMICs

To enhance the utilization of quality assured and accurate HCV diagnostic platforms in all countries (in particular to LMICs), concrete efforts are needed, not only to consider regionally and most affected population-representative clinical validation studies through data sharing or systematic reviews, but also to establish other cohesive and stringent international standards/mechanisms [48]. Although WHO PQ provides commendable inroads to assess the quality of HCV-IVD platforms used specifically in resource constraint settings, and the WHO’s provided Essential Diagnostic List (Table 2) is laudable for currently used HCV assays, additional procedures to accelerate and synchronize national assay approval and registration are urgently needed. In this context, the efforts are underway and an exemplary guidance series has already been streamlined which provides a framework to design and conduct local validation studies for national laboratories and generic protocols produced by the International Diagnostic Center (i.e., designed by the London School of Hygiene and Tropical Medicine) for CD4 and HIV viral load detection could also be valuable to rationalize HCV diagnostics. The development of an online dashboard with an open access database could also be valuable to synchronize existing real-world HCV clinical diagnostic performance data collected from laboratory settings and peer-reviewed publications with unpublished and government-led studies to help with the WHO PQ or CE-IVD validation process for HCV diagnostic platforms, assays, and tests which are already in the pipeline. Although this approach requires the significant engagement of data sources at the country level and time-consuming to ensure data quality, nevertheless, it could be very fruitful to have a check on transparency and performance to known high-performing tests. These databases could provide greater visibility of the clinical accuracy of high-performance HCV screening and diagnostic tests, as well as the generation and acquisition to more diverse local performance data by using not yet SRA approved, but performing excellent diagnostic platforms. It may also raise awareness about the risks associated with purchasing cheaper diagnostic products of unassured quality and compromised performance than those on a list of approved assays [48].

#### 5.3.3. Expediting the Review Process for the Approval of Current HCV Diagnostic Platforms in LMICs

The registration and regulatory approval pathway for diagnostic platforms and auxiliary accessories of regional companies that are interested in the marketing of their products may be relatively easier, although this depends on various factors including significant costs [66]. Furthermore, in many LMICs, the need for SRA approval for HCV testing platforms is often less clear, and in many countries, the approval processes seem vague depending entirely on existing and often unreliable package insert data which solely provides the whole test performance information. Consequently, inaccurate diagnostic results can potentially impact both public health outcomes and individual HCV patient management in LMICs due to which procurement of unapproved or unqualified HCV screening and diagnostic tests should be prohibited. The inroads of SRA approval, WHO PQ, and HCV diagnostic accuracy specifications in LMICs could be easier by providing additional trained staff, funding, and expediting the review process of intended diagnostic platforms to be used. In addition to that, procuring higher volume tests can reduce or may negotiate lower prices [66].

#### 5.3.4. Open Licensing Agreement for the Utilization of Current HCV Diagnostic Platforms

It seems likely to be unattainable, but many LMICs can negotiate with major stakeholders of the HCV diagnostic market to consider an open licensing agreement for their diagnostic platforms [48]. One good initiative in this context has been taken by the BLINK company by developing an open-license, polyvalent diagnostic platform (i.e., in the early prototype stage). The company is working on a POC HCV RNA assay with a total turnaround time of fewer than 20 min. The company offers to use or ‘rent’ its technology to perform the chemical reactions needed to diagnose the disease by using any assay develops by any developer. Furthermore, the company has already minimized the cost by simplifying the design of their diagnostic unit, reducing the complexity and number of parts, the usability of disposable materials, and the recycling ability of plastic consumables. It is a simple example of an integrated service delivery approach and to bring down diagnostics costs if multiple and larger volume test bundles can be procured. Governments and procurement agencies on their behalf can also take measures to streamline registration and licensing of WHO PQ diagnostics and may save middle-man bureaucratic costs through the WHO Collaborative Registration Procedure (CRP) [125]. This procedure was initially launched for an HIV viral load platform, m-PIMA Analyser (Abbott)—piloted in Cameroon, Cote d’Ivoire, Ethiopia, Nigeria, and Tanzania at the end of 2019. If rationalized for HCV diagnostic platforms, this process could be very handy to save time, improving access to generic HCV diagnostics by simplifying country national registration steps, tackling regulatory bottlenecks, and ensuring product sameness with the WHO’s PQ essential diagnostic list [125].

#### 5.3.5. Enhancing Funding for HCV Diagnostic Research and Development (R&D)

Both public and philanthropic funding is a prerequisite for the development of medicine and diagnostics. Although the design and development pathway of a diagnostic assay seems low cost and low risk as compared to drug development, the process of designing to penetration into the market of a new diagnostic assay can take minimally 7 years and needs to overcome two “valleys of death” before launch [66]. We find a chronic lack of funding and an overwhelming lack of interest in developing and implementing HCV diagnostic platforms for LMICs where the markets are not competitive and strongly developed despite the potential of consuming bulk diagnostic platforms and higher volumes tests [8,125]. Another aspect is that when innovative technologies become viable and proven candidates, large stakeholders (i.e., companies) acquire those to benefit privatized, under patent, or exclusive licensing [48]. This has been experienced for FibroScan and FibroTest/ActiTest, (i.e., an assay developed by French public research institutions and universities to monitor liver stiffness, the level of hepatic damage, and the degeneration of liver cells in viral hepatitis patients) for which the companies set exorbitant pricing, making it almost unaffordable for many LMICs [48]. Perhaps, FIB-4 and APRI tests could be an alternative to the FibroTest to assess liver damage in many LMICs; however, their operation requires lab facilities, additional patient visits, and is time-consuming for result interpretations before an individual is diagnosed with HCV and started DAAs treatment [48].

Research and development (R&D) in medicine and diagnostics based on public funding should remain affordable and accessible to everyone who needs them. Public institutions’ finance in the R&D of technologies can be reimbursed by preparing the licensing agreement for their diagnostic platforms, tests, and assays and by increasing their access and utility in public health sectors and various communities [48]. In Canada, Universities Allied for Essential Medicines, through student advocacy, adopted a Global Access Licensing Framework at universities to provide a transparent and equitable licensing framework on medicines to increase access in LMICs. A similar approach can be rationalized to open up licensing on HCV diagnostic technologies. Furthermore, the demand for already approved, simpler, affordable, and easily accessible POC diagnostics by understanding their significant benefits can also help to break registration and licensing monopolies in the LMICs. Lastly, SRA approval or meeting HCV diagnostic accuracy specifications in LMICs can be considered through generating external independent data as a part of their national registration regulations and by the transparent tendering process to ensure procurement and implementation of high-quality diagnostic platforms [48].

### 5.4. Cost and Pricing Barriers

#### 5.4.1. Potential Causes of High Costs for Current HCV Diagnostic Algorithms

The costs of HCV diagnostic platforms and pricing of HCV screening and confirmatory tests would always be remained a key barrier to scale-up or launch national HCV screening campaigns in HCV-vulnerable populations as well as in highly prevalent HCV settings in many LMICs where a diagnostic platform can be priced out of reach due to its estimated costs, which could be in the tens of thousands of US dollars [125]. Diagnostic platforms procured through international donor funding also require specific cartridges, kits, system maintenance, and with the limitations of use to only the disease areas that fit within the scope and broad areas of interest of the donor-funded projects [48]. At the county level, distributor markups, sales taxes, and heavy customs fees present additional cost barriers. At the customer level, local distribution mark-ups increase the final testing price up to sky-high. A survey conducted among health care workers from India, Indonesia, Malaysia, Morocco, and Thailand demonstrated immense barriers to the HCV care cascade resulting from ‘out-of-pocket’ costs in both public and private health care sectors [125]. In public healthcare centers, limited staff for HCV testing and a long patient waiting list further adds more time to individual testing or treatment uptake. Individuals have to take extra days off work for each diagnostics pathway visit, which turns into a waste of time and income as a part of overall test costs.

#### 5.4.2. Cost-Effectiveness of Simplified HCV Diagnostic Pathway in LMICs

A large-scale trial participating over 11,000 PWIDs across 12 sites in India demonstrated an overall significant impact on HCV testing and increased treatment uptake when offered an integrating free HCV Abs screening in HIV and harm reduction centers [126]. DBS sample analysis being widely integrated with HIV programs could also be a rapid, effective, and affordable method for HCV EIA and viral load testing in outreach, remote, and resource-deficient settings [13]. Furthermore, HCV self-testing (both in terms of HCV Abs screening and viral RNA confirmation) is becoming very handy in some resource-replete nations with telemedicine support and monitoring to integrate highly stigmatized HCV communities to care cascade [10,15]. Real-life studies (e.g., SELFIE and ICECREAM) are underway to evaluate the feasibility of HCV-self testing among MSM, to evaluate their impact on HCV diagnostics and care pathway in terms of time to diagnose, treatment initiation for reinfections, and prevent further infection transmission [125]. However, it is too early to comment about their applicability and implementation in LMICs with limited telemedicine infrastructure and needs extensive investigations before their implementation.

#### 5.4.3. Cost-Effectiveness of Simplified HCV Diagnostic Pathway to Launch National Test-and-Treat Programs

A simplified diagnostic pathway both applicable for individual screening and ‘national test-and-treat programs’ signifies its importance to scale up HCV diagnosis and further treatment uptake. The lowest market prices considering all components of an HCV patient screening and treatment pathway should be USD 79/patient for an FDC of SOF + DCV per 12-week course and USD 94/patient for individual SOF + DCV per 12-week course by using/following Global Fund’s pooled procurement mechanism [48]. Similarly, data from in-country advocates demonstrate HCV test pricing at USD 15–30/test in the public health center to USD 60–200/test in the private health sectors depending on the product specifications and country geographical distribution [48].

By analogy, in Rwanda, a 5-year HCV elimination program based on the ‘test-and-treat strategy’ was proposed to target and screen around 4 million individuals aged ≤15, of whom 230,000 were expected to be HCV Abs positive and required viral RNA load testing to confirm viremic HCV infection, and 112,000 were expected to be treated as chronically infected [125]. Through raised awareness, the low costs of HCV screening and treatment, and international stakeholders’ solidarity, the program included one HCV Abs RDT, one confirmatory RNA viral load test, a 12-week treatment course, and one SVR12 test at post-treatment completion to confirm cure. The program aims to decrease the current HCV prevalence rate from 4% to 1.2% and to achieve a 90% treatment uptake of all individuals aged ≤15 by 2024. The program also assumes to conduct a pretreatment assessment of liver staging tests, pregnancy assessment, genotype identification of harder-to-treat HCV populations, comorbidities risks, and possible drug–drug interactions in screening and treating patients. The program also focused on implementing affordable test and treatment services, as well as providing effective and innovative models of health services delivery to affected individuals by community awareness, scale-up of HCV screening at primary healthcare centers, and training of non-expert and non-specialist HCPs. So far, around 1 million Rwandans have already been screened after the launch of this campaign, and 15,000 have undergone DAAs regimens and have been cured. In addition to the involvement of other factors, the key factor for the successful running of this program lies in the reduced HCV diagnostic costs from USD 30 to USD 1 and decreased treatment costs from USD 8000 to USD 60 [125].

One study conducted in Cambodia also signifies the importance of cost-effectiveness of a simple diagnostic pathway to scaling up HCV diagnosis, with a reduced cost of USD 376 (Interquartile Range USD 344–422) [127]. This Cambodian ‘test-and-treat’ program is almost similar to Rwanda by following the WHO’s simplified testing guidelines (Figure 6). However, associated costs for two educational/awareness sessions, a pretreatment assessment by a FibroScan exam to check liver staging, two consultations with physicians and one with a pharmacist, and related time/salaries are also included in this diagnostic and cure package [127].

We have discussed above the national screening program in Egypt, which successfully showed the feasibility of nationwide HCV screening of 50 million people over 7 months [120] (Figure 1(1b)). The reduced costs of diagnostic algorithms (i.e., an anti-HCV RDT (USD 0.58 per test) and HCV RNA confirmatory test (USD 4.80 per test)) through mass procurement after discussions with a single negotiating body was a key factor to achieve this massive-scale population screening for HCV in millions of Egyptians. Indeed, the low prices of HCV diagnostic and treatment package in this campaign could be a benchmark for other LMICs to reach reduced costs. Lastly, a simplified HCV care cascade model with affordable test and treatment costs can save money and results in better cure outcomes as compared to an HCV care pathway of long-term costs associated with undiagnosed and untreated HCV patients. The money saved in this manner could help health budgets to integrate and fully cover other health care services expenses and diagnostics costs. Waiving sales taxes and exemption of customs fees for essential HCV diagnostics could also be helpful to expediting combat against HCV in many LMICs and setting caps on local distributor’s kickbacks could also put a price control and reduce end-user test prices. The transparent process of HCV diagnostic pricing and volume-based discounts offered by companies on mass procurement of the products could also be considered and negotiated by the governments in LMICs while making procurement decisions.

### 5.5. n-SARS-CoV-2 and the COVID-19 Pandemic

#### 5.5.1. The Virus That Shook the World Has Also Halted Hepatitis C Diagnosis

Since its first emergence in Wuhan, China, in 2019, the novel coronavirus (nCoV-19) and the associated pandemic coronavirus disease (COVID-19) have badly disrupted the world-leading health care systems and jolted the global economy. Since then, the pandemic has been prevailed in around 185 nations of the world while affecting 150 million people worldwide including 3.1 million deaths to date. The pandemic has created an unprecedented effect on all aspects of a society, where governments and health care authorities have reinforced and are still emphasizing lockdowns, social distancing, and pandemic prevention guidelines as a whole, protecting guided measures to slow down the transmission of this highly transmissible disease. The raised death tolls have worsened the health conditions of the individuals already living with HCV and PWIDs due to self-isolation, pending or missed medical appointments, and lack of to access their essential routine health services [125]. The reallocation of existing health care systems, services platforms, and enormous amounts of public funding to battle against COVID-19 has compounded the complications of providing essential health services, let alone the scaling up of HCV diagnostics and treatment response [8]. Furthermore, many non-emergency services have been paused or altered by health departments, and laboratory-based assessments of innovative models of HCV diagnostic platforms and tests that emerged before COVID-19 have been stopped or postponed [8,125]. HCV self-test validations also experienced delays as diagnostic companies started a race and rushed to approve and market SARS-CoV-2 virus diagnostics [125]. Clinical studies have shown elevated hepatic enzymes in people who recovered from COVID-19 while living with viral hepatitis, liver fibrosis, and other hepatic comorbidities and will need to monitor their liver function tests following recovery [128]. The pandemic has also interrupted routine health visits and mass screening programs to confirm viremic HCV infections and identify new cases as protective measures to reduce people’s risk exposure to the SARS-CoV-2 virus.

#### 5.5.2. Plausible Solutions to Continue HCV Screening during the COVID-19 Pandemic Period

In such unusual circumstances, alongside COVID-19 testing, ‘opt-out HCV Abs screening’ and ‘reflex RNA testing’ in emergency rooms, in pharmacies, or at drive-through screening sites could ensure some continuity with adequate diagnostic supplies and cooperation of HCPs [125]. Furthermore, during this difficult time, countries with a history of harm reduction services have already included NSEPs and OAT for OUDs as essential services [8]. The COVID-19 pandemic is still ongoing and may well last until 2021 or 2022, so in the post-pandemic period, wider community distribution of OAT (e.g., naloxone) and take-home as well as home deliverance of DAAs and OAT combined with telemedicine, treatment monitoring, and social support could also be helpful to prevent the new HCV incidences in vulnerable populations if done so permanently [8]. Countries should focus on efforts to curb the overlapping HCV, substance use overdose, and COVID-19 pandemics. The huge investments to combat the COVID-19 pandemic both at diagnostic and treatment levels should be used to strengthen lab infrastructures, increasing testing capacities, improving supply chains, develop the healthcare workforce, raise public awareness on infectious diseases, expand multi-disease platforms, and approach the outreach of HCV/COVID-19 coinfected communities [8]. Many companies have adopted, diversified, and modified their already existing HCV diagnostic platforms (e.g., COBAS TaqMan^®^ (Roche^®^), GeneXpert^®^ (Cepheid^®^, Sunnyvale, CA, USA), Genedrive, RealTime (Abbott^®^), and ARCHITECT^®^ (Abbott^®^)) for COVID-19 tests [125]. After ending the COVID-19 pandemic, the lessons learned from the diagnostic platforms used for COVID-19 testing and health care services provided for patient management and treatment could also be aligned with HCV elimination strategies to simplify and decentralize diagnostic pathway as well as to integrate into existing essential healthcare infrastructures and facilities (e.g., HIV, sexual health, and harm reduction centers) [8]. Local distributors and government health care stakeholders in LMICs may negotiate for the procurement of discounted, volume-based deals for HCV diagnostics and assays alongside COVID-19 testing algorithms.

## 6. Future Promises

“Everything should be made as simple as possible, but no simpler” (Albert Einstein 1879–1955).

This famous quote of Albert Einstein can be applied to any discovery in science in particular to medical diagnosis where the world is badly suffering from endemics, epidemics, and pandemics of microbial pathogens and viruses. It is troublesome for the world that, despite the latest advancement in medicine, infection diagnosis, and treatment, we are only successful in eliminating ‘the measles’, and other contagious and infectious viral infections are still prevailing. It seems overambitious that without ‘simplification of diagnosis’ and ‘treatment for all who need them’, we would be able to combat or eradicate viral infections from the globe. Consequently, the next implementation of research should be focused on the development of simplified diagnosis devices, tests, and strategies to find everyone who is affected by an infection and linkage to care. Herein, we briefly highlight the future expectations from the current and emerging but simplified HCV diagnostic techniques to escalate hep C elimination efforts.

### 6.1. The Next 5-Year Perspective

The world has reached the cusp of an HCV diagnostic revolution where we have now innovative commercial digital devices supported with robust assays to detect HCV RNA (i.e., active viremic infection) within 60 to 90 min, in a single visit, and with on-site HCV diagnosis. However, challenges remain for assay turn-around time, test costs, and the need for auxiliary instruments to perform POC HCV RNA testing [13]. The technology is turning so fast that, in the next 4–5 years, it is possible that fully portable or wearable HCV RNA detection diagnostic devices with a result time around 10 min that are user-friendly, low cost, and with smartphone-like data sharing facilities will be available. However, the implication of all this in real-world clinical diagnosis will require huge investments for diagnostic space, implementation research, and healthcare infrastructure to unlock the HCV diagnostic market. Further investments will be required for rapid evaluation of the new state-of-the-art HCV diagnostic devices and assays to recognize their regulatory status with WHO PQ and CE marking followed by their approvals in regions/countries where those are desperately needed to scale up HCV diagnosis. To transform the novel HCV diagnostic technologies and treatment strategies from diagnostic/treatment pipeline to real-world clinical practice, the researchers, health care practitioners, government health ministries, diagnostic and pharma industrialists, social activists, and affected HCV communities must work together to improve the life expectancy of people living with HCV and to amalgamate the efforts to escalate HCV diagnosis and care pathway to eliminate HCV infection as a major public health threat by 2030.

### 6.2. The Next 10 Years

An important synergy exists within the screening, diagnosis, prevention, and management programs of HIV, STDs, and viral hepatitis [8]. This integration should be widened by raising general public awareness about the substantial HCV infection burden to healthcare services and by better leveraging funding amongst key stakeholders because HCV test-and-treat programs are facing a chronic lack of funding worldwide. Efforts should be directed to provide affordable, automated, and rapid POC tests to launch mass test-and-treat campaigns in LMICs as well as the targeted screening of high-risk populations in less prevalent HCV locations. The programs must be built to facilitate one-stop HCV diagnosis in outreach sites for highly vulnerable HCV populations and prioritize treatment access to PWIDs, migrant IDUs, and incarcerated peoples, for instance. Liver health education and literacy programs are in very urgent need to expand in many LMICs to increase knowledge about HCV transmission risks, harmful consequences of CHC infection, and most importantly to convey the advantages of initial diagnosis and treatment uptake. It would be beneficial to build the mind and escalate the will of suspected HCV-affected individuals to be screened and may overcome the stigmatization of HCV infections. For this purpose, HCV education could be confined or customized by interactive tele-education for high risks groups (e.g., PWIDs, MSM, sex-workers), and for correctional or marginalized populations (e.g., prisoners, pregnant women, children, adolescents, WORA with drug abuse, migrants/refugees) to raise their knowledge about the risks of HCV infection, the consequences of high-risk behaviors, and to make them aware about the existing public health policies that could facilitate or support them if they get infected [8]. Blood safety services, harm reduction centers, and correctional institutes should also be improved and upgraded to reduce HCV transmission and should provide allied health services to treat OUDs, HIV, and STDs alongside management of HCV for stigmatized or criminalized HCV affected individuals by the support of government and social activists. International investments should be spent on HCV elimination programs with equity in LMICs while focusing on prevention and treatment strategies. The HCV treatment coverage can be raised by reducing the costs of DAAs and by introducing generic pan-genotypic agents to cure in resource-constrained nations. The efforts to develop effective HCV vaccines should be carried on which will facilitate to accomplish the progress toward elimination goals of hepatitis C by 2030.

Considerable work is also likely to be done in the coming years to provide simplified HCV care pathways with equal and affordable access to everyone who is needed in the world. For this purpose, in many LMICs, the availability of HCV RNA POCT that could perform reliably from FS whole blood, serum or plasma, DBS, or DPS specimen interface would be an important solution. Other plausible diagnostic concepts for many LMICs, remote, or hard-to-reach locations are ‘near-to-patient diagnostics’ using mobile molecular biology lab units, following the ‘lab-on-a-drone’ or ‘rent-a-POCT’ concepts using either current or upcoming diagnostic approaches [103]. To implement these HCV diagnostic concepts in real-life conditions, R&D of several innovative devices which are already in the late stages of development and are based on ‘loop-mediated isothermal amplification’, ‘smartphone-operated instruments’, ‘biosensors’, ‘lab-on-a-chip solutions’, ’paper-based microfluidics’, and ‘CRISPR-Cas’ transformational technologies must be sped up. Despite all these impressive real-life efforts and relatively quick diagnostic and short-term HCV cure, not all countries will probably get rid of HCV by 2030; at least in several countries HCV elimination does not seem possible with the WHO time frame or beyond. It is also important to note that achieving the WHO’s targets to eliminate HCV by 2030 does not mean that all individuals living with HCV have been diagnosed and cured in those countries/regions which are successfully on the track to eliminate HCV [8]. Hence, even removing HCV from the WHO’s list of major public health threats by 2030, sustained considerations on HCV elimination policy, affordable availability of an ideal and pragmatic test-and-treat approach, global and philanthropic research funding, consolidated and concrete political will, and stronger mobilization of social activists will be pre-requisite with keeping an eye on this ‘silent epidemic’ issue.

## 7. Conclusions

The rapid advancement in HCV elimination strategies both in terms of scaling up HCV diagnosis and enhancing treatment uptake has come a long way within the previous 4 to 5 years since the WHO designed and published the global health strategy on viral hepatitis. Since then, 11 nations are on the track to achieve the HCV elimination goal as a major public health threat by the 2030 deadline. The simplification of the HCV care cascade including rapid HCV screening, infection diagnosis, treatment uptake, and cure monitoring as the realistic goals of their national hepatitis plan (Figure 5) by some nations as well as international guidelines have resulted in substantial individual and global efforts to minimize new HCV outbreaks and enhanced the cure for HCV affected populations. The detection of active HCV viremic infection through sophisticated semi-digital diagnostic platforms with robust assays as well as the treatment of almost all HCV GTs infected populations with pan-genotypic DAAs signifies the importance of a simplified ‘test-and-treat’ strategy to achieve the objectives of HCV elimination. The guidelines and successful national hepatitis plans of the nations that are on the course to achieve HCV elimination by or before 2030 also reflect that a simplified HCV diagnosis and treatment approach can be very vital to clinical decision-making for quick infection diagnosis and rapid treatment initiation based on available clinical trials and large real-world cohorts’ data. It also emphasizes that other existing guidelines and national hepatitis plans (Figure 5) should be updated accordingly in many LMICs who have poor healthcare infrastructure or inefficiently developed HCV diagnostics or care settings to continuously evolve the simplified test-and-treat strategy to ensure that they reach HCV elimination. The collection of the standardized venipuncture blood specimen, their transportation through shipment or courier, and HCV testing within centralized laboratories will be continued in the next 5 years. In parallel, decentralized models of HCV diagnosis will flourish in settings such as drug harm reduction centers and alcohol clinics, sexual health clinics, community health care centers, mental disorder clinics, prisons, NSEPs, practitioner consumption rooms, and other outreach community places with mobile services. DBS and DPS sampling analysis for active viremic infection will be more common in places where on-site phlebotomy services are not available and poor venous access in PWIDs and IDUs in rural/remote areas and many LMICs are a major problem to collect whole blood venous specimens. Furthermore, such approaches will be beneficial to collect large patient pool data through DBS sample analysis as well as FS POC HCV RNA assays for the application of receiving regulatory approval of various commercial HCV RNA assays including CE-marking and WHO PQ. The fast-tracking transformational technology advancement in the advent of digital diagnostics will lead to a robust, quick (i.e., <10 min assay turnaround time), user-friendly, and low-cost FS POC HCV RNA assay with the probability of a wearable device that is battery-powered and without the requirement of the auxiliary instruments very soon. On-site, one-test, single–visit, and near-to-patient care diagnosis will be the norm in many settings with the practical implantation of HCV-self test card technology, lab-on-a-drone, rent-a-POC-test, and mobile microbiological laboratories concepts in real-life clinical diagnostic conditions. All these proposed advancements in HCV diagnostic algorithms will be helpful to expand HCV testing globally in high-risk populations as well as in highly prevalent HCV settings in many LMICs, will accelerate HCV linkage to care and treatment, and will amalgamate the collaboration of health care systems and care-givers to achieve WHO defined HCV elimination targets.

## Figures and Tables

**Table 1:** The advantages and limitations of current and emerging HCV screening and diagnostic approaches. The table compares current and emerging HCV screening and diagnostic platforms in terms of their accessibility, affordability, durability, testing turnaround time, and cost-effectiveness [48]. Ab = antibody, POC = point-of-care testing, LMICs = low to middle income countries, LLoD = lower limit of detection, VL = viral load, FS = finger stick, DBS = dried blood spot, PQ = prequalification, CE = Conformité Européenne, TB = tuberculosis, HPV = human papillomavirus, SRA = stringent regulatory approval.

Anti-HCV Abs Tests		HCV EIA Tests	
Advantages	Disadvantages	Advantages	Disadvantages
Easy to use (i.e., better for task shifting)	Low throughput	High sensitivity and high specificity (approx. 100%)	Effective specimen transport system required
Suitable for oral fluid, plasma, serum, saliva, finger prick, and whole blood	Inadequate for patients with low Ab titer or with the compromised immune system	High sample throughput (good for large volumes)	A cold chain environment from 2 °C to 8 °C is required for reagents and not always possible in LMICs
Feasible for decentralization HCV testing	Subjective interpretation (i.e., operator dependent)	Low cost if used for high volume samples	Additional precise equipment requires (e.g., micropipette, centrifuges)
Equipment-free and suitable for POC analysis with small lab infrastructure	Possible higher cost based on volume	Data processing-based results	Possible higher cost based on volume
Good sensitivity and specificity	Compromised quality control	Cost-effective for a large number of samples	Turnaround time usually longer (one batch runs 96 samples), 12 or 24 samples test configuration required for different volume
Qualitative results interpretation (i.e., yes/no)		Easier oversight quality control	Interference from different sample matrices
Fast turnaround time (i.e., ~20 min)		Polyvalency for serological screening of HIV, HBV, etc.	Requires technically skilled staff
1–2 years shelf life if stored properly at room temperature		1–2 years shelf life if stored properly at room temperature	Equipment calibration, maintenance, and service required
**HCV cAg tests**		**HCV RNA confirmatory test platform (Abbott Realtime^®^)**	
Good to conduct HCV cAg tests for large sample settings (i.e., 100 samples/h)	Cannot use without an effective specimen transport system	Good to conduct HCV RNA tests for high volume settings (i.e., 93 samples/h)	An effective specimen transport system required
The testing platform also suitable for HIV screening based on licensing in different countries (i.e., polyvalency)	Not licensed for use with DBS samples	Also suitable for multiple disease screening (e.g., HIV, HCV genotyping) based on licensing in different countries	Not licensed for use with DBS samples
Advantageous to detect cAg in the bloodstream (i.e., 2 weeks after infection) than anti-HCV ab tests (i.e., 10 weeks after infection)	Only used for diagnostic confirmation; cannot confirm HCV cure	Low cost for high volume samples	HCV test cost: USD 11–23 (GF price) which may vary depends on volume and term commitment
Low cost if used for high volume samplesCost-effective for a large number of samples	Less sensitive with chances of miss infections	Easier oversight quality control	Centralized lab settings are required to handle large volume specimens
Easier oversight quality control	Centralized lab settings are required to handle large volume specimens		Turnaround time usually longer
	Turnaround time usually longer		Skilled technical staff requires for instrument handling
	Skilled technical staff requires for instrument handling		Equipment calibration, maintenance, and service required
	Equipment calibration, maintenance, and service required		
	HCV test cost: USD 8–23		
**HCV RNA confirmatory test platform (Cepheid GeneXpert^®^)**		**HCV RNA confirmatory test platform (RocheCobas^®^Taqman)**	
Xpert VL has an HCV RNA quantitative assay for plasma samples and Xpert VL FS for fingerstick whole blood samples	Xpert VL and Xpert VL FS are not available in many countries and not approved in the USA	Suitable for both HCV RNA qualitative and quantitative RNA confirmation (93 tests/batch)	Phase-out for the Cobas^®^ 4800 and 6800 systems
Xpert VL HCV RNA assay is CE marked and has WHO PQ certifications	High cartridge costs for Xpert VL in many countries	HCV RNA qualitative test is cheaper than quantitative	Effective specimen transport system required
Xpert VL FS is also CE-marked and WHO PQ	Biowaste incineration may not be available in decentralized settings for both Xpert VL and Xpert VL FS platforms	Low cost with reduced test pricing to 85 LMICs	Not licensed for use with DBS samples
Xpert VL FS is suitable for HCV-infected IDUs with poor vein access	Lab infrastructure is required for GeneXpert instrument and reagents	Efficient automated system	Turnaround time usually longer
Xpert VL FS cartridge is feasible for simplification in sample collection	Transport cost and centrifuges are required for plasma specimens used of Xpert VL platforms	Polyvalency for screening multiple diseases (e.g., TB, HIV, HPV, HBV, etc.) based on licensing in different countries	Lab infrastructure is required to house equipment
Xpert VL is very accurate, simple to operate, and good for task shifting to facilitate decentralized HCV testing	Distributor mark-ups, additional services, and maintenance costs required	Easier oversight quality control	Skilled technical staff requires for instrument handling despite fully automated
Xpert VL turnaround time is around 108–110 min on the same day while Xpert VL FS in 60 min	Fixed test price regardless of sample volumes		Equipment service and maintenance required
Having an extensive test menu for Integration of testing across other disease programs (e.g., TB, HIV, HPV, HBV, etc.)			Preferential pricing is not publically available
Preferential pricing in 145 LMICs			
**HCV RNA confirmatory test platform (Genedrive^®^HCV)**		**Dried blood spot (DBS) samples**	
Suitable for HCV RNA POC testing	High cost per patient sample	An alternative to plasma samples	An organized, strong, and effective transport system for sample delivery
Small portable unit (weighs 1 kg)	Both device and test pricing are high	Suitable for small volume samples collected by fingerstick	Reduced analytical sensitivity for RNA analysis due to small sample volume
Qualitative HCV RNA analysis (+ve/−ve/undetected)	Sensitivity is less than lab-based methods (i.e., LLoD is 2362 IU/mL than the recommended 1000 IU/mL for other HCV RNA POC tests)	An alternative to RDTs, in harm reduction centers or prison settings	No immediate delivery of results to the patients
Turnaround time 90 min, suitable for same-day diagnosis	Low throughput per device	Suitable for HCV Ab testing, genotyping, and treatment resistance testing	Quality standards need to develop
Suitable for few numbers of patients/day	A large volume sample (i.e., 30 µL plasma) is required which could be challenging in POC settings	Useful for HCV vertical transmission detection in infants and children	Limited use for research purposes because of lack of SRA status for companies
Room temperature storage for reagents	Not fully automated, some steps require precise micro pipetting for sample/reagents	Inexpensive	Scale-up of DBS in LMICs requires additional studies and validation work
	Requires uninterrupted power supply, which may not available in some LMICs	Suitable for mass/large HCV screening campaigns in vulnerable populations and highly prevalent HCV settings	Requires uninterrupted power supply, which may not available in some LMICs
	Only valid for HCV testing and not appropriate for multi-disease testing (i.e., polyvalency)	Appropriate for decentralized collection of samples to deliver by courier in central labs	Only valid for HCV testing and not appropriate for multi-disease testing
		No risk of biohazard because of nonhazardous sample interface	
		Room temperature transportation	
		Low level of training and suitable for task-shifting	

**Table 2:** The WHO essential diagnostic list of current and emerging HCV diagnostic platforms and tests with WHO prequalification, are CE marked, and are in late-stage development. ND = not defined, PQ = prequalification, CE = Conformitè Europëenne, LMICs = low-to-middle-income countries, CE-IVD = Conformitè Europëenne in vitro diagnostics, POC = point of care, NAT = nucleic acid testing, HAV = hepatitis A virus, IVD= in vitro diagnostics, TB = tuberculosis, GTs = genotyping, TK = to come. (Source: Activist Guide to Hepatitis C Virus Diagnostics; Available from: https://www.hepcoalition.org/advocate/advocacy-tools/article/activist-guide-to-hepatitis-c-virus-diagnostics; accessed on 2 April 2021).

Test Name	Sample Type/Setting	Turnaround Time	Multiplexing	Price	Regulatory Status	WHO PQ Review	System Suitability
**1. HCV Enzyme Immunoassay (HCV-EIA) tests**							
INNOTEST HCV Ab IV	Serum, plasma	179 min	Yes (HIV & other markers)	ND	WHO PQ	-	Central labs
INNO-LIA HCV Score	Serum, plasma	1 day	No	ND	WHO PQ CE-marked	-	Strip-based method but requires cold chain and other small equipment
Bioelisa HCV 4.0	Serum, plasma	150 min	Yes (HIV, HBV, HEV, among others)	ND	WHO PQ, CE-marked	-	Central labs
Murex Anti-HCV 4.0	Serum, plasma	120 min	Yes (HIV, HBV, HEV, among others)	ND	WHO PQ, CE-marked	-	Central labs
Enzygnost Anti-HCV 4.0	Serum, plasma	120 min	Yes (HBV, HAV)	ND	Did not receive WHO PQ approval	-	Central labs
**2. HCV core antigen (HCV cAg) tests**							
Abbott ARCHITECT HCV Ag assay	Serum, plasma	ND	Yes (HIV, HBV, HAV, among others)	USD 8–23 (EUR 7–20) per test; USD 100,000 per instrument	CE-marked, WHO PQ	-	Central labs
Monolisa HCV Ag-Ab ULTRA V2	Serum, plasma	150–180 min	Yes (HIV, HBV, HAV, among others)	ND	CE-marked,	WHO PQ (Jan 2020)	Central labs
**3. HCV Rapid diagnostic tests (HCV-RDTs)**							
OraQuick HCV RDT	Oral, fingerstick, venous blood	20 min	No	USD 8 (MSF price); USD 12 per test	FDA Approved CE-marked WHO PQ	-	Price remains too expensive for LMICs
SD Bioline	10 μL whole blood, serum, plasma	5–20 min	HIV	USD 1–2.40 per test	WHO PQ	-	-
Intec Rapid anti-HCV	10 μL, whole blood, serum, plasma	15–20 min	No	<USD 1–2.40 per test	WHO PQ, CE-marked	-	-
Standard Q HCV Ab Test	Whole blood, serum, plasma	10–15 min	No	ND	CE-IVD	WHO PQ March 2020	POC
Premier Medical Corporation First Response^®^ HCV Card Test	Whole blood, serum, plasma	ND	No	USD 0.60–1 per test	CE-marked	WHO PQ under review	-
ABON HCV Rapid Test Device	Whole blood, serum, plasma	ND	No	ND		WHO PQ under review	-
**4. HCV RNA viral load tests**							
Cepheid Xpert VL Assay (use with Cepheid GeneXpert)	Plasma samples, can be POC	Same-day results in 108–110 min	HIV, TB	USD 17,000 per instrument; USD 14.90 per test (for all virological tests in LMICs)	WHO PQ, CE-marked, not approved in United States	-	Can be used for all genotypes; need WHO recommendations for use in all genotypes
Cepheid Xpert VL Fingerstick	100 μL, capillary blood, Fingerstick Tertiary PoC: harm reduction settings, maybe easier to give for some PWID with poor vein access	Within 60 min	HIV	ND	CE-IVD; Not FDA-approved	Modified version of the VL assay; CE-marked, not approved in United States	Tertiary POC, harm reduction settings
Genedrive HCV RNA	30 μL plasma, serum	90 min	No	USD 5000 per device; USD 25-35 per test USD 30–40/test in India USD 14–18/test for Africa	CE-IVD	WHO PQ (May 2020)	POC; Decentralized diagnosis
RealTime HCV Viral Load	0.5 mL plasma, 0.2 mL serum, DBS (FS)	ND	HIV	USD 11–23 per test; Global Fund price varies according to test volume/term commitment	CE-IVD (for HIV DBS and HCV RNA)	WHO PQ	Lab HCV viral load, All six GTs detected
Abbott Alinity m HCV assay RNA	Plasma, serum	135 min	No	USD 50 per test	FDA-approved in March 2020	CE-IVD, WHO PQ (March 2020)	Lab, clinical. All HCV GTs detected
Hologic Aptima HCV Quant	Serum, plasma	ND	HIV	USD 10–15 per test; USD 12 (€12–15) all-inclusive price for HCV VL	FDA- approved	-	CE-marked; Lab-based
Biocentric Generic HCV PCR assay	Serum, plasma	ND	HIV	USD 23 per test, USD 13.50–17 per test	-	-	CE-marked
Roche Cobas 6800/8800 systems (HCV RNA)	Lab-based, High volume clinical settings	First 96 results <3.5 h, every 90 min for 96 more results (Cobas 6800 System); first 96 results <3.5 h, every 30 min for 96 more results (Cobas 8800 System)	HIV	USD 35–45 per test; USD 340,000-USD 475,000 per instrument (depending on instrument and volume)	-	-	CE-marked, FDA approved
**5. HCV diagnostics pipeline or late-stage development tests**							
ARCHITECT HCV cAg assay	Serum, DBS	ND		USD 8–23	CE-IVD	WHO PQ	Lab
HCV Self-Test	Oral fluid, capillary blood	-	-	<USD 4 per test	TK	-	PoC; sexual health clinics, harm reduction centers, at home
Well anti-HCV RDT; Self-Test	Oral	15–20 min		TK	TK	-	POC; primary care, at-home settings
TrueNAT HCV PCR	250 µL whole blood, 500 µL plasma	40 min	polyvalent	USD 10,000 (small); USD 14,000–18,000 (large) USD 18 per test, depending on volume	CDSCO-India approval in 2019;	CE-IVD and WHO PQ (Estimated in 2020)	POC; primary healthcare
COBAS^®^ Plasma Separation Card	Whole blood, plasma, dried plasma spots	-	-	TK, GPRO range: USD 10–15 per test Egypt (high-volume): USD 7 per test	Not FDA approved	CE-IVD (for HIV only, in development for HCV in 2020/2021)	lab
DBS on various PCR platforms	0.2 mL serum DBS (fngerstick)	-	-	TK	RealTime HCV Viral load, CE-IVD (for HIV DBS and HCV RNA); WHO PQ (Dec 2019) COBAS^®^ AmpliPrep/COBAS TaqMan HCV Test CE-IVD (for HIV only) Aptima^®^ HCV Quant Dx Assay (TK)	-	Lab

## Data Availability

Not applicable.

## References

[1] Blach S., Zeuzem S., Manns M., Altraif I., Duberg A.S., Muljono D.H., Waked I., Alavian M.S., Lee M.H., Negro F. (2017). Global prevalence and geno-type distribution of hepatitis C virus infection in 2015: A modelling study. Lancet Gastroenterol. Hepatol..

[2] World Health Organization (WHO) Global Hepatitis Report 2017 (Internet). https://www.who.int/hepatitis/publications/global-hepatitis-report2017/en/.

[3] World Health Organization (2019). Hepatitis C Fact Sheet.

[4] hepCoalition (2018). Sofosbuvir Turns 5 Years Old: The Vast Majority of People with Chronic Hepatitis C still Have not Been Treated. https://hepcoalition.org/IMG/pdf/factsheet_sofosbuvir_5_anniversary-2.pdf.

[5] Cooke G., Andrieux-Meyer I., Applegate T.L., Atun R., Burry J.R., Cheinquer H., Dusheiko G., Feld J.J., Gore C., Griswold M. (2019). Lancet commission: Accelerating the elimination of viral hepatitis. Lancet Gastroenterol. Hepatol..

[6] European Centre for Disease Prevention and Control (2018). Hepatitis C. https://ecdc.europa.eu/en/hepatitis-c.

[7] Association Française pour l’Etude du Foie (2017). Recommandations AFEF sur la Prise en Charge de L’hepatite Virale C. http://www.afef.asso.fr/ckfinder/userfiles/files/recommandationstextesofficiels/recommandations/RecommandationsAFEFMars2017.pdf.

[8] Cox A.L., El-Sayed M.H., Kao J.-H., Lazarus J.V., Lemoine M., Lok A.S., Zoulim F. (2020). Progress towards elimination goals for viral hepatitis. Nat. Rev. Gastroenterol. Hepatol..

[9] Hill A.M., Nath S., Simmons B. (2017). The road to elimination of hepatitis C: Analysis of cures versus new infec-tions in 91 countries. J. Virus Erad..

[10] Patel A.A., Bui A., Prohl E., Bhattacharya D., Wang S., Branch A.D., Perumalswami P.V. (2021). Innovations in Hepatitis C Screening and Treatment. Hepatol. Commun..

[11] Fourati S., Feld J.J., Chevaliez S., Luhmann N. (2018). Approaches for simplified HCV diagnostic algorithms. J. Int. AIDS Soc..

[12] Chevaliez S. (2019). Strategies for the improvement of HCV testing and diagnosis. Expert Rev. Anti-Infect. Ther..

[13] Grebely J., Applegate T., Cunningham P., Feld J.J. (2017). Hepatitis C point-of-care diagnostics: In search of a single visit diagnosis. Expert Rev. Mol. Diagn..

[14] Hepatitis C Cure, Sofosbuvir, Turns 5 Years Old: The Vast Majority of People Still Nave Not Been Treated. https://hepcoalition.org/news/press-releases/article/hepatitis-c-cure-sofosbuvir-turns-5-years-old-the-vast-majority-of-people-still.

[15] Pawlotsky J.-M., Ramers C.B., Dillon J., Feld J.J., Lazarus J. (2020). Simplification of Care for Chronic Hepatitis C Virus Infection. Semin. Liver Dis..

[16] Duffell E.F., Hedrich D., Mardh O., Mozalevskis A. (2017). Towards elimination of hepatitis B and C in European Union and European Economic Area countries: Monitoring the World Health Organization’s global health sector strategy core indicators and scaling up key interventions. Eurosurveillance.

[17] European Centre for Disease Prevention and Control (ECDC) (2016). Systematic Review on Hepatitis B and C Prevalence in the EU/EEA..

[18] European Monitoring Centre for Drugs and Drug Addiction (EMCDDA) (2016). Hepatitis C among Drug users in Europe. Epidemiology, Treatment and Prevention.

[19] Wiessing L., Ferri M., Grady B., Kantzanou M., Sperle I., Cullen K.J., Hatzakis A., Prins M., Vickerman P., EMCDDA DRID Group (2014). Hepatitis C Virus Infection Epidemiology among People Who Inject Drugs in Europe: A Systematic Review of Data for Scaling Up Treatment and Prevention. PLoS ONE.

[20] Hofmeister M.G., Rosenthal E., Barker L.K., Rosenberg E.S., Barranco M.A., Hall E.W., Edlin B.R., Mermin J., Ward J.W., Ryerson A.B. (2019). Estimating Prevalence of Hepatitis C Virus Infection in the United States, 2013–2016. Hepatology.

[21] Rich J.D., Beckwith C.G., Macmadu A., Marshall B.D.L., Brinkley-Rubinstein L., Amon J.J., Milloy M.J., King M., Sanchez J., Atwoli L. (2016). Clinical care of incarcerated people with HIV, viral hepatitis, or tuberculosis. Lancet.

[22] Beckwith C.G., Kurth A.E., Bazerman L., Solomon L., Patry E., Rich J.D., Kuo I. (2015). Survey of US Correctional Institutions for Routine HCV Testing. Am. J. Public Health.

[23] Emory Center for the Health of Incarcerated Persons and MGH Institute for Technology Assessment. http://www.hepcorrections.org/.

[24] Nijmeijer B.M., Koopsen J., Schinkel J., Prins M., Geijtenbeek T.B. (2019). Sexually transmitted hepatitis C virus infections: Current trends, and recent advances in understanding the spread in men who have sex with men. J. Int. AIDS Soc..

[25] European Centre for Disease Prevention and Control (ECDC) (2016). Epidemiological Assessment of Hepatitis B and C Prevalence among Migrants in the EU/EEA.

[26] Dibba P., Cholankeril R., Li A.A., Patel M., Fayek M., Dibble C., Okpara N., Hines A., Ahmed A. (2018). Hepatitis C in Pregnancy. Diseases.

[27] Indolfi G., Easterbrook P., Dusheiko G., El-Sayed M.H., Jonas M.M., Thorne C., Bulterys M., Siberry G., Walsh N., Chang M. (2019). Hepatitis C virus infection in children and adolescents. Lancet Gastroenterol. Hepatol..

[28] Ly K.N., Jiles R.B., Teshale E.H., Foster M.A., Pesano R.L., Holmberg S.D. (2017). Hepatitis C Virus Infection Among Reproductive-Aged Women and Children in the United States, 2006 to 2014. Ann. Intern. Med..

[29] Ward C., Tudor-Williams G., Cotzias T., Hargreaves S., Regan L., Foster G.R. (2000). Prevalence of hepatitis C among pregnant women attending an inner London obstetric department: Uptake and acceptability of named antenatal testing. Gut.

[30] Lambert J., Jackson V., Coulter-Smith S., Brennan M., Geary M., Kelleher T.B., O’Reilly M., Grundy K., Sammon N., Cafferkey M. (2008). Universal antenatal screening for Hepatitis C at an Irish maternity hospital. Am. J. Obstet. Gynecol..

[31] Diab-Elschahawi M., Dosch V., Honsig C., Jatzko B., Segagni L., Assadian O., Presterl E. (2013). Evaluation of a uni-versal vs a targeted hepatitis C virus screening strategy among pregnant women at the Vienna University Hospital. Am. J. Infect. Control.

[32] Jhaveri R., Broder T., Bhattacharya D., Peters M.G., Kim A.Y., Jonas M.M. (2018). Universal screening of preg-nant women for hepatitis C: The time is now. Clin. Infect. Dis..

[33] Schillie S., Wester C., Osborne M., Wesolowski L., Ryerson A.B. (2020). CDC Recommendations for Hepatitis C Screening Among Adults—United States, 2020. MMWR Recomm. Rep..

[34] Chaillon A., Rand E.B., Reau N., Martin N.K. (2019). Cost-effectiveness of Universal Hepatitis C Virus Screening of Pregnant Women in the United States. Clin. Infect. Dis..

[35] Rose M.M.J., Evans J., Prince A., Espinosa C. Hepatitis C risk based vs. universal screening among preg-nant women: Implementation and cost-effectiveness analysis. Proceedings of the Liver Meeting November.

[36] Oliver C., Black J., De Pont S., Sizemore L., Wester C. (2019). Pregnancy Status, Risk Factors, and Opportunities for Referral to Care Among Reproductive-Aged Women with Newly Reported Chronic Hepatitis C Virus Infection in Tennessee. Public Health Rep..

[37] Saab S., Kullar R., Gounder P. (2020). The urgent need for hepatitis C screening in pregnant women: A call to action. Obstet. Gynecol..

[38] Modin L., Arshad A., Wilkes B., Benselin J., Lloyd C., Irving W.L., Kelly D.A. (2020). Epidemiology and natural history of hepatitis C virus infection among children and young people. J. Hepatol..

[39] Smith B.D., Morgan R.L., Beckett G.A., Falck-Ytter Y., Holtzman D., Ward J.W. (2012). Hepatitis C Virus Testing of Persons Born During 1945–1965: Recommendations from the Centers for Disease Control and Prevention. Ann. Intern. Med..

[40] The European Union HCV Collaborators (2017). Hepatitis C virus prevalence and level of intervention required to achieve the WHO tar-gets for elimination in the European Union by 2030: A modelling study. Lancet Gastroenterol. Hepatol..

[41] Negro F. (2014). Epidemiology of hepatitis C in Europe. Dig. Liver Dis..

[42] Yehia B.R., Schranz A., Umscheid C.A., Re V.L. (2014). The Treatment Cascade for Chronic Hepatitis C Virus Infection in the United States: A Systematic Review and Meta-Analysis. PLoS ONE.

[43] American Association for the Study of Liver Diseases (AASLD), Infectious Diseases Society of America (IDSA) (2017). HCV Guidance: Recommendations for Testing, Managing and Treating Hepatitis C. www.hcvguidelines.org.

[44] World Health Organization (WHO) (2016). WHO Guidelines on Hepatitis B and C Testing. http://apps.who.int/iris/handle/10665/.

[45] European Association for the Study of the Liver (2016). EASL Recommendations on Treatment of Hepatitis C 2016. J. Hepatol..

[46] Udompap P., Mannalithara A., Heo N.-Y., Kim D., Kim W.R. (2016). Increasing prevalence of cirrhosis among U.S. adults aware or unaware of their chronic hepatitis C virus infection. J. Hepatol..

[47] Spradling P.R., Tong X., Rupp L.B., Moorman A.C., Lu M., Teshale E.H., Gordon S.C., Vijayadeva V., Boscarino J.A., Schmidt M.A. (2014). Trends in HCV RNA testing among HCV antibody-positive per-sons in care, 2003–2010. Clin. Infect. Dis..

[48] Activist Guide to Hepatitis C Virus Diagnostics. https://www.hepcoalition.org/advocate/advocacy-tools/article/activist-guide-to-hepatitis-c-virus-diagnostics.

[49] Reipold E.I., Easterbrook P., Trianni A., Panneer N., Krakower D., Ongarello S., Roberts T., Miller V., Denkinger C. (2017). Optimising diagnosis of viraemic hepatitis C infection: The development of a target product profile. BMC Infect. Dis..

[50] Zarei M. (2017). Advances in point-of-care technologies for molecular diagnostics. Biosens. Bioelectron..

[51] Radin J.M., Topol E.J., Andersen K.G., Steinhubl S.R. (2016). A laboratory in your pocket. Lancet.

[52] Romao V.C., Martins S.A.M., Germano J., Cardoso F.A., Cardoso S., Freitas P.P. (2017). Lab-on-Chip Devices: Gaining Ground Losing Size. ACS Nano.

[53] Gupta E., Agarwala P., Kumar G., Maiwall R., Sarin S.K. (2017). Point-of-care testing (POCT) in molecular diagnostics: Performance evaluation of GeneXpert HCV RNA test in diagnosing and monitoring of HCV infection. J. Clin. Virol..

[54] Grebely J.L., Lamoury F.M.J., Hajarizadeh B., Mowat Y., Marshall A.D., Bajis S., Marks P., Amin J., Smith J., Edwards M. (2017). Evaluation of the Xpert HCV Viral Load point-of-care assay from venipuncture-collected and finger-stick capillary whole-blood samples: A cohort study. Lancet Gastroenterol. Hepatol..

[55] Lamoury F.M.J., Bajis S., Hajarizadeh B., Marshall A.D., Martinello M., Ivanova E., Catlett B., Mowat Y., Marks P., Amin J. (2018). Evaluation of the XpertHCV Viral Load Fingerstick point-of-care assay. J. Infect. Dis..

[56] Duchesne L., Lacombe K. (2017). Innovative technologies for point-of-care testing of viral hepatitis in low-resource and decentralized settings. J. Viral Hepat..

[57] World Health Organization (2017). Essential Medicines and Health Products: In the Lead-up to Paris AIDS Conference, WHO Prequalifies First Generic Hepatitis C Medicine and First HIV Self-Test. http://www.who.int/medicines/news/2017/1st_generic-hepC_1stHIVself-test-prequalified/en/.

[58] Nasrullah M., Sergeenko D., Gvinjilia L., Gamkrelidze A., Tsertsvadze T., Butsashvili M., Metreveli D., Sharvadze L., Alkhazashvili M., Shadaker S. (2017). The role of screening and treatment in national progress to-ward hepatitis C elimination—Georgia, 2015–2016. MMWR Morb. Mortal Wkly. Rep..

[59] Mitruka K., Tsertsvadze T., Butsashvili M., Gamkrelidze A., Sabelashvili P., Adamia E., Chokheli M., Drobeniuc J., Hagan L., Harris A.M. (2015). Launch of a Nationwide Hepatitis C Elimination Program-Georgia, April 2015. MMWR Morb. Mortal Wkly. Rep..

[60] Gvinjilia L., Nasrullah M., Sergeenko D., Tsertsvadze T., Kamkamidze G., Butsashvili M., Gamkrelidze A., Imnadze P., Kvaratskhelia V., Chkhartishvili N. (2016). National Progress Toward Hepatitis C Elimination—Georgia, 2015–2016. MMWR Morb. Mortal Wkly. Rep..

[61] Kim D.D., Hutton D.W., Raouf A.A., Salama M., Hablas A., Seifeldin I.A., Soliman A.S. (2015). Cost-effectiveness model for hepatitis C screening and treatment: Implications for Egypt and other countries with high prevalence. Glob. Public Health.

[62] WHO (2016). Global Health Sector Strategy on Viral Hepatitis 2016—Towards Ending Viral Hepatitis.

[63] Warkad S.D., Nimse S.B., Song K.-S., Kim T. (2018). HCV Detection, Discrimination, and Genotyping Technologies. Sensors.

[64] EASL (2018). Recommendations on treatment of hepatitis C 2018. J. Hepatol..

[65] EASL (2017). Recommendations on Treatment of Hepatitis C 2016. J Hepatol..

[66] Applegate T.L., Fajardo E., Sacks J.A. (2018). Hepatitis C Virus Diagnosis and the Holy Grail. Infect. Dis. Clin. N. Am..

[67] Warkad S.D., Song K.-S., Pal D., Nimse S.B. (2019). Developments in the HCV Screening Technologies Based on the Detection of Antigens and Antibodies. Sensors.

[68] Cameron S.O., Carman W.F. (2005). The use of the OraSure^®^ collection device for hepatitis virus testing in health care settings. J. Clin. Virol..

[69] Shivkumar S., Peeling R., Jafari Y., Joseph L., Pant P.N. (2012). Accuracy of rapid and point-of-care screening tests for hepatitis C: A systematic review and meta-analysis. Ann. Intern. Med..

[70] Chevaliez S., Poiteau L., Rosa I., Soulier A., Roudot-Thoraval F., Laperche S., Hézode C., Pawlotsky J.M. (2016). Prospective assessment of rapid diagnostic tests for the detection of antibodies to hepatitis C virus, a tool for improving access to care. Clin. Microbiol. Infect..

[71] Greenman J., Roberts T., Cohn J., Messac L. (2015). Dried blood spot in the genotyping, quantification and storage of HCV RNA: A systematic literature review. J. Viral Hepat..

[72] Scalioni L.P., Cruz H.M., de Paula V.S., Miguel J.C., Marques V.A., Villela-Nogueira C.A., Milagres F.A., Cruz M.S., Bastos F.I., Andrade T.M. (2014). Performance of rapid hepatitis C virus antibody assays among high- and low-risk populations. J. Clin. Virol..

[73] Cha Y.J., Park Q., Kang E.S., Yoo B.C., Park K.U., Kim J.W., Hwang Y.S., Kim M.H. (2013). Performance evaluation of the OraQuick hepatitis C virus rapid antibody test. Ann. Lab. Med..

[74] Pallarés C., Carvalho-Gomes Â., Hontangas V., Conde I., Di Maira T., Aguilera V., Benlloch S., Berenguer M., López-Labrador F.X. (2018). Performance of the OraQuick Hepatitis C virus antibody test in oral fluid and fingerstick blood before and after treat-ment-induced viral clearance. J. Clin. Virol..

[75] Khan H., Hill A., Main J., Brown A., Cooke G. (2017). Can Hepatitis C Virus Antigen Testing Replace Ribonucleic Acid Polymearse Chain Reaction Analysis for Detecting Hepatitis C Virus? A Systematic Review. Open Forum Infect. Dis..

[76] Duchesne L., Njouom R., Lissock F., Tamko-Mella G.F., Rallier S., Poiteau L., Soulier A., Chevaliez S., Vernet G., Rouveau N. (2017). HCV Ag quantification as a one-step procedure in diagnosing chronic hepatitis C infection in Cameroon: The ANRS 12336 study. J. Int. AIDS Soc..

[77] Cresswell F.V., Fisher M., Hughes D.J., Shaw S.G., Homer G., Hassan-Ibrahim M.O. (2015). Hepatitis C core anti-gen testing: A reliable, quick, and potentially cost effective alternative to hepatitis C polymerase chain reac-tion in diagnosing acute hepatitis C virus infection. Clin. Infect. Dis..

[78] Kamal S.M., Kassim S., El Gohary E., Fouad A., Nabegh L., Hafez T., Bahnasy K., Hassan H., Ghoraba D. (2015). The accuracy and cost-effectiveness of hepatitis C core antigen assay in the monitoring of anti-viral therapy in patients with chronic hepatitis C genotype *Aliment*. Pharmacol. Ther..

[79] Cohn J., Roberts T., Amorosa V., Lemoine M., Hill A. (2015). Simplified diagnostic monitoring for hepatitis C, in the new era of direct-acting antiviral treatment. Curr. Opin. HIV AIDS.

[80] Abdollahi-Aghdam A., Majidi M.R., Omidi Y. (2018). Microfluidic paper-based analytical devices (µPADs) for fast and ultrasensitive sensing of biomarkers and monitoring of diseases. BioImpacts BI.

[81] Eletxigerra U.J., Martinez-Perdiguero S., Merino R., Villalonga J., Pingarrón J.M., Campuzano S. (2014). Amperometric magnetoimmunoassay for the direct detection of tumor necrosis factor alpha biomarker in human serum. Anal. Chim. Acta.

[82] Ghanbari K., Roushani M., Azadbakht A. (2017). Ultra-sensitive aptasensor based on a GQD nanocomposite for detection of hepatitis C virus core antigen. Anal. Biochem..

[83] Roh C., Lee H.Y., Kim S.E., Jo S.K. (2010). Quantum-dots-based detection of hepatitis C virus (HCV) NS3 using RNA aptamer on chip. J. Chem. Technol. Biotechnol..

[84] Easterbrook P.J., Roberts T., Sands A., Peeling R. (2017). Diagnosis of viral hepatitis. Curr. Opin. HIV AIDS.

[85] Lange B., Cohn J., Roberts T., Camp J., Chauffour J., Gummadi N., Ishizaki A., Nagarathnam A., Tuaillon E., van de Perre P. (2017). Diagnostic accuracy of serological diagnosis of hepatitis C and B using dried blood spot samples (DBS): Two systematic reviews and meta-analyses. BMC Infect. Dis..

[86] Vázquez-Morón S., Jiménez B.A., Jiménez-Sousa M.A., Bellón J.M., Ryan P., Resino S. (2019). Evaluation of the diagnostic accuracy of laboratory-based screening for hepatitis C in dried blood spot samples: A systematic review and meta-analysis. Sci. Rep..

[87] Lange B., Roberts T., Cohn J., Greenman J., Camp J., Ishizaki A., Messac L., Tuaillon E., van de Perre P., Pichler C. (2017). Diagnostic accuracy of detection and quantification of HBV-DNA and HCV-RNA using dried blood spot (DBS) samples—A systematic review and meta-analysis. BMC Infect. Dis..

[88] Catlett B., Bajis S., Starr M., Dore G.J., Hajarizadeh B., Cunningham P.H., Applegate T.L., Grebely J. (2020). Evaluation of the Aptima HCV Quant Dx Assay for HCV RNA detection from finger-stick capillary dried blood spot and venepuncture-collected samples. J. Infect. Dis..

[89] 2020 Pipeline Report. https://www.treatmentactiongroup.org/resources/pipeline-report/2020-pipeline-report/last.

[90] Nguyen L., Nguyen V., Le Ai K., Truong M., Tran T., Jamil M., Johnson C., Reipold E., Easterbrook P., Park K. (2021). Acceptability and Usability of HCV Self-Testing in High Risk Populations in Vietnam. Diagnostics.

[91] Liu L., Zhang M., Hang L., Kong F., Yan H., Zhang Y., Feng X., Gao Y., Wang C., Ma H. (2020). Evaluation of a new point-of-care oral anti-HCV test for screening of hepatitis C virus infection. Virol. J..

[92] Kimble M.M., Stafylis C., Treut P., Saab S., Klausner J.D. (2019). Clinical evaluation of a hepatitis C antibody rapid immunoassay on self-collected oral fluid specimens. Diagn. Microbiol. Infect. Dis..

[93] Vetter B.N., Reipold E.I., Ongarello S., Fajardo E., Tyshkovskiy A., Ben I., Vasylyev M. (2021). Prospective evaluation of hepatitis C virus antibody detection in whole blood collected on dried blood spots with the INNOTEST^®^ HCV Ab IV enzyme immunoassay. J. Clin. Virol..

[94] Padhi A., Gupta E., Singh G., Agarwal R., Sharma M.K., Sarin S.K. (2020). Evaluation of the Point of Care Molecular Diagnostic Genedrive HCV ID Kit for the detection of HCV RNA in clinical samples. Epidemiol. Infect..

[95] Marins E.G., Krey N., Becker A., Melzer S., Hoppler M. (2020). Evaluation of the cobas^®^ HCV test for quantifying HCV RNA in dried plasma spots collected using the cobas^®^ Plasma Separation Card. J. Virol. Methods.

[96] Prinsenberg T., Rebers S., Boyd A., Zuure F., Prins M., Van Der Valk M., Schinkel J. (2020). Dried blood spot self-sampling at home is a feasible technique for hepatitis C RNA detection. PLoS ONE.

[97] Van Tilborg M., Al Marzooqi S.H., Wong W.W., Maan R., Vermehren J., Maasoumy B., Mazzulli T., Bolotin S., Garber G., Guerra F. (2018). HCV core antigen as an alternative to HCV RNA testing in the era of direct-acting antivirals: Retrospective screening and diagnostic cohort studies. Lancet Gastroenterol. Hepatol..

[98] Biondi M.J., Van Tilborg M., Smookler D., Heymann G., Aquino A., Perusini S., Mandel E., Kozak R.A., Cherepanov V., Kowgier M. (2019). Hepatitis C Core-Antigen Testing from Dried Blood Spots. Viruses.

[99] Evaluation of Dried Blood Spot for HCV RNA Testing Evaluation of Dried Blood Spot for HCV RNA Testing—Full Text View. ClinicalTrials.gov.

[100] Chantratita W., Song K.-S., Nimse S.B., Pongthanapisith V., Thongbaiphet N., Wongtabtim G., Pasomsub E., Angkanavin K., Sonawane M.D., Warkad S.D. (2017). 6 HCV Genotyping 9G test for HCV 1a, 1b, 2, 3, 4 and 6 (6a, 6f, 6i and 6n) with high accuracy. J. Virol. Methods.

[101] Warkad S.D., Nimse S.B., Song K.-S., Chantratita W., Pongthanapisith V., Nawale L.U., Kim T. (2018). Performance of 6 HCV genotyping 9G test for HCV genotyping in clinical samples. Virol. J..

[102] Warkad S.D., Nimse S.B., Song K.-S., Kim T. (2020). Development of a Method for Screening and Genotyping of HCV 1a, 1b, 2, 3, 4, and 6 Genotypes. ACS Omega.

[103] Poljak M. (2020). Simplification of hepatitis C testing: A time to act. Acta Dermatovenerol. Alp. Pannonica Adriat..

[104] Saludes V., Antuori A., Lazarus J., Folch C., González-Gómez S., González N., Ibáñez N., Colom J., Matas L., Casabona J. (2020). Evaluation of the Xpert HCV VL Fingerstick point-of-care assay and dried blood spot HCV-RNA testing as simplified diagnostic strategies among people who inject drugs in Catalonia, Spain. Int. J. Drug Policy.

[105] Velásquez-Orozco F., Rando-Segura A., Martínez-Camprecios J., Salmeron P., Najarro-Centeno A., Esteban À., Quer J., Buti M., Pumarola-Suñe T., Rodríguez-Frías F. (2021). Utility of the Cobas^®^ Plasma Separation Card as a Sample Collection Device for Serological and Virological Diagnosis of Hepatitis C Virus Infection. Diagnostics.

[106] Antuori A., Montoya V., Piñeyro D., Sumoy L., Joy J., Krajden M., González-Gómez S., Folch C., Casabona J., Matas L. (2021). Characterization of acute HCV infection and transmission networks in people who currently inject drugs in Catalonia: Usefulness of dried blood spots. Hepatology.

[107] Saludes V., Antuori A., Folch C., González N., Ibáñez N., Majó X., Colom J., Matas L., Casabona J., Martró E. (2019). Utility of a one-step screening and diagnosis strategy for viremic HCV infection among people who inject drugs in Catalonia. Int. J. Drug Policy.

[108] Saludes V., Folch C., Morales-Carmona A., Ferrer L., Fernàndez-López L., Muñoz R., Jiménez M., Loureiro E., Fernández-Dávila P., Bascuñana E. (2017). Community-based screening of hepatitis C with a one-step RNA detection algorithm from dried-blood spots: Analysis of key populations in Barcelona, Spain. J. Viral Hepat..

[109] Valerio H., Alavi M., Silk D., Treloar C., Martinello M., Milat A., Dunlop A., Holden J., Henderson C., Amin J. (2021). Progress Towards Elimination of Hepatitis C Infection Among People Who Inject Drugs in Australia: The ETHOS Engage Study. Clin. Infect. Dis..

[110] Catlett B., Carrera A., Starr M., Applegate T.L., Lowe P., Grebely J., Cunningham H.P. (2019). Performance evaluation of the Hologic Aptima HCV Quant Dx assay for detection of HCV RNA from dried blood spots. J. Clin. Virol..

[111] Bregenzer A., Warmann N., Ottiger C., Fux C.A. (2019). Rapid point-of-care HCV RNA quantification in capillary whole blood for diagnosing chronic HCV infection, monitoring treatment and detecting reinfection. Swiss Med. Wkly..

[112] Prabdial-Sing N., Gaelejwe L., Makhathini L., Thaver J., Manamela M.J., Malfeld S., Spearman C.W., Sonderup M., Scheibe A., Young K. (2021). The performance of hepatitis C virus ( HCV ) antibody point-of-care tests on oral fluid or whole blood and dried blood spot testing for HCV serology and viral load among individuals at higher risk for HCV in South Africa. Health Sci. Rep..

[113] Pollock K.G., McDonald S.A., Gunson R., McLeod A., Went A., Goldberg D.J., Hutchinson S.J., Barclay S.T. (2020). Real-world utility of HCV core antigen as an alternative to HCV RNA testing: Implications for viral load and genotype. J. Viral Hepat..

[114] Lamoury F.M., Soker A., Martinez D., Hajarizadeh B., Cunningham E.B., Cunningham P., Bruggmann P., Foster G.R., Dalgard O., Backmund M. (2017). Hepatitis C virus core antigen: A simplified treatment monitoring tool, including for post-treatment relapse. J. Clin. Virol..

[115] Wong X.Z., Gan C.C., Mohamed R., Yahya R., Ganapathy S., Tan S.S., Lim S.K. (2020). Hepatitis C core antigen testing to diagnose active hepatitis C infection among haemodialysis patients. BMC Nephrol..

[116] Vetter B.N., Reipold E.I., Ongarello S., Audu R., Ige F.A., Alkhazashvili M., Chitadze N., Vanroye F., De Weggheleire A., An S. (2020). Sensitivity and Specificity of Rapid Diagnostic Tests for Hepatitis C Virus With or Without HIV Coinfection: A Multicentre Laboratory Evaluation Study. J. Infect. Dis..

[117] Tsertsvadze T., Gamkrelidze A., Chkhartishvili N., Abutidze A., Sharvadze L., Kerashvili V., Butsashvili M., Metreveli D., Gvinjilia L., Shadaker S. (2019). Three years of progress towards achieving hepatitis C elimination in the country of Georgia, April 2015–March 2018. Clin. Infect. Dis..

[118] Averhoff F., Shadaker S., Gamkrelidze A., Kuchuloria T., Gvinjilia L., Getia V., Sergeenko D., Butsashvili M., Tsertsvadze T., Sharvadze L. (2020). Progress and challenges of a pioneering hepatitis C elimination program in the country of Georgia. J. Hepatol..

[119] Shiha G., Soliman R., Mikhail N.N., Easterbrook P. (2020). An educate, test and treat model towards elimination of hepatitis C infection in Egypt: Feasibility and effectiveness in 73 villages. J. Hepatol..

[120] Waked I., Esmat G., Elsharkawy A., El-Serafy M., Abdel-Razek W., Ghalab R., Elshishiney G., Salah A., Abdel Megid S., Kabil K. (2020). Screening and Treatment Program to Eliminate Hepatitis C in Egypt. N. Engl. J. Med..

[121] Konerman M.A., Thomson M., Gray K., Moore M., Choxi H., Seif E., Lok A.S. (2017). Impact of an electronic health record alert in primary care on increasing hepatitis c screening and curative treatment for baby boomers. Hepatology.

[122] International Treatment Preparedness Coalition-Global (2018). Activist Toolkit on PrEP.

[123] Degenhardt L., Peacock A., College S., Leung J., Grebely J., Vickerman P., Stone J., Cunningham E.B., Trickey A., Dumchev K. (2017). Global prevalence of injecting drug use and sociodemographic characteristics and prevalence of HIV, HBV, and HCV in people who inject drugs: A multistage systematic review. Lancet Glob. Health.

[124] Annual Meeting of the Canadian Association for the Study of the Liver (CASL), the Canadian Network on Hepatitis C (CANHEPC) and the Canadian Association of Hepatology Nurses (CAHN) 2020 Abstracts. https://canlivj.utpjournals.press/doi/pdf/10.3138/canlivj.3.1.abst.

[125] Collaborative Registration Procedure (CRP) for In Vitro Diagnostics (IVDs)—Information Note. https://www.who.int/diagnostics_laboratory/191111_crp_ivd_information_note.pdf.

[126] Solomon S.S., Quinn T.C., Solomon S., McFall A.M., Srikrishnan A.K., Verma V., Kumar M.S., Laeyendecker O., Celentano D.D., Iqbal S.H. (2020). Integrating HCV testing with HIV programs improves hepatitis C outcomes in people who inject drugs: A cluster-randomized trial. J. Hepatol..

[127] Walker J.G., Mafirakureva N., Iwamoto M., Campbell L., Kim C.S., Hastings R., Doussett J., Le Paih M., Balkan S., Marquardt T. (2020). Cost and cost-effectiveness of a simplified treatment model with direct-acting antivirals for chronic hepatitis C in Cambodia. Liver Int..

[128] Wang Y., Liu S., Liu H., Li W., Lin F., Jiang L., Li X., Xu P., Zhang L., Zhao L. (2020). SARS-CoV-2 infection of the liver directly contributes to hepatic impairment in patients with COVID-19. J. Hepatol..

